# Comparative genomics of transport proteins in developmental bacteria: *Myxococcus xanthus* and *Streptomyces coelicolor*

**DOI:** 10.1186/1471-2180-13-279

**Published:** 2013-12-05

**Authors:** Ilya Getsin, Gina H Nalbandian, Daniel C Yee, Ake Vastermark, Philipp CG Paparoditis, Vamsee S Reddy, Milton H Saier

**Affiliations:** 1 Department of Molecular Biology, University of California at San Diego, La Jolla, CA 92093-0116, USA

**Keywords:** Transport proteins, *Streptomyces*, *Myxococcus*, Genome analyses

## Abstract

**Background:**

Two of the largest fully sequenced prokaryotic genomes are those of the actinobacterium, *Streptomyces coelicolor* (Sco), and the δ-proteobacterium, *Myxococcus xanthus* (Mxa), both differentiating, sporulating, antibiotic producing, soil microbes. Although the genomes of Sco and Mxa are the same size (~9 Mbp), Sco has 10% more genes that are on average 10% smaller than those in Mxa.

**Results:**

Surprisingly, Sco has 93% more identifiable transport proteins than Mxa. This is because Sco has amplified several specific types of its transport protein genes, while Mxa has done so to a much lesser extent. Amplification is substrate- and family-specific. For example, Sco but not Mxa has amplified its voltage-gated ion channels but not its aquaporins and mechano-sensitive channels. Sco but not Mxa has also amplified drug efflux pumps of the DHA2 Family of the Major Facilitator Superfamily (MFS) (49 versus 6), amino acid transporters of the APC Family (17 versus 2), ABC-type sugar transport proteins (85 versus 6), and organic anion transporters of several families. Sco has not amplified most other types of transporters. Mxa has selectively amplified one family of macrolid exporters relative to Sco (16 versus 1), consistent with the observation that Mxa makes more macrolids than does Sco.

**Conclusions:**

Except for electron transport carriers, there is a poor correlation between the types of transporters found in these two organisms, suggesting that their solutions to differentiative and metabolic needs evolved independently. A number of unexpected and surprising observations are presented, and predictions are made regarding the physiological functions of recognizable transporters as well as the existence of yet to be discovered transport systems in these two important model organisms and their relatives. The results provide insight into the evolutionary processes by which two dissimilar prokaryotes evolved complexity, particularly through selective chromosomal gene amplification.

## Background

Spore formation is common within the prokaryotic world. Endospores can be found in a variety of Gram-positive bacteria, including species of *Bacillus*, *Clostridium*, *Metabacterium* and *Thermoactinomyces*[1]. Aerial exospore formation is common among species of *Streptomyces*[2]. *Dermatophilus* form zoospores [3], while *Azotobacter* form resting cysts [4]. Myxospores are common among the Myxobacteria, including species of *Myxococcus* and *Stigmatella*[5]. Other resting cell types can be found in cyanobacteria such as *Anabaena*[6]. The best characterized of the sporulation processes is endospore formation in *Bacillus subtilis*[7]. However, aerial mycelial exospores in actinobacteria and fruiting body bearing myxospores in myxobacteria provide alternatives for understanding the molecular bases of complex multicellular prokaryotic differentiation.

The two organisms that serve as model systems to represent these two phyla are *Streptomyces coelicolor* (Sco) and *Myxococcus xanthus* (Mxa). Both organisms interact and produce antibiotics and a variety of other secondary metabolites, rendering them important for medical and biotechnological purposes [8-10]. Some gene families such as regulatory gene families are amplified; for example, Sco has 44 ser/thr protein kinases and Mxa has 97, although most bacteria have only 0–3. The genomes of these two organisms have been fully sequenced, and they prove to be among the largest prokaryotic genomes currently available for analysis, both being about 9 million base pairs (Mbp) in size [11,12]. Because of the unique features of these two organisms, we have conducted a thorough investigation of the transport proteins encoded within their genomes.

Transport proteins serve as important mediators of communication between the cell cytoplasm and the extracellular environment [13]. They frequently allow transmission of signals that determine transcription patterns and progression into programs of differentiation [14]. They also determine whether or not secondary metabolites such as antibiotics will be synthesized, exported, or imported [15]. We have therefore initiated a study to determine what transporters are likely to be important for these processes and whether or not these two complex organisms share these systems.

In this paper, we analyze the genomes of Sco and Mxa for all integral membrane transport proteins that correspond to currently recognized transporters included within the Transporter Classification Database TCDB; http://www.tcdb.org; [16-18]. These systems fall into several classes, including (1) channels/pores, (2) secondary carriers, (3) primary active transporters, (4) group translocators, (5) transmembrane electron flow carriers, (8) auxiliary transport proteins, and (9) transporters of unknown mechanism of action. The identified proteins are analyzed by class, topology and substrate specificity, and the results are compared.

Our analyses reveal that these two organisms use fundamentally different systems to transport various substrates, suggestive of independent evolution. While Sco has amplified the numbers of transporters in certain families specific for certain types of substrates (e.g. sugars, amino acids, organic anions), Mxa has not. Moreover, they use very different types of transporters for the purpose of extruding antimicrobial agents. The results lead to the conclusion that Sco and Mxa have used very different strategies to create programs of differentiation and solve metabolic problems created by the development of multiple cell types.

## Results

### ***Streptomyces coelicolor *** **(Sco) Transporters**

**Figure 1:**
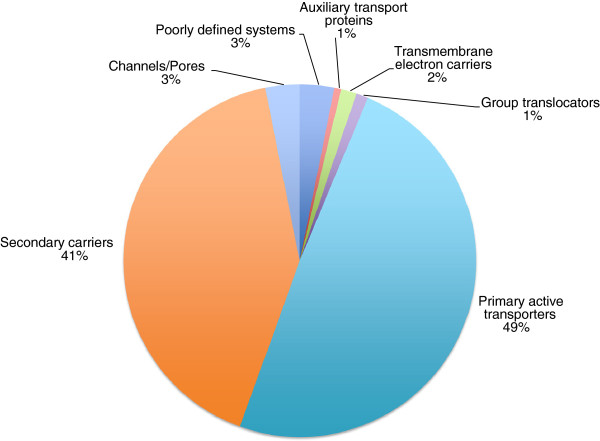
***Streptomyces coelicolor *transporter type percentages.** Transporter type percentages in *Streptomyces coelicolor*, based on the Transporter Classification (TC) system.

For the purpose of genome analyses, we classify transport systems according to the IUBMB-approved Transporter Classification (TC) System. Transporters fall into five well-defined categories (Classes 1 to 5) and two poorly defined categories (Classes 8 and 9) as mentioned above, (see TCDB; http://www.tcdb.org; [13,18-20]). Additional file 1: Table S1 and Figure 1 present an overall summary of the classes and subclasses of transporters found in *Streptomyces coelicolor* (Sco). Only integral membrane transport proteins, mostly those that provide the transmembrane pathway for solute translocation, are reported. We identified 658 such proteins encoded in the Sco genome. The entire genome is 9.05 million base pairs and is reported to encode 7825 proteins [11]. Thus, 8.1% of the proteins encoded within the genome of Sco are recognized integral membrane transport proteins. Functionally characterized and partially characterized transporters reported in the literature are tabulated and discussed below (see section entitled “Transporters of experimentally verified function in Sco and Mxa”).

#### ***Types of transporters in Sco***

Sco encodes representatives within the major classes of transport proteins included in TCDB, and their distributions are summarized here (see Table 1): 20 (3%) of these proteins are simple channels; 277 (41%) are secondary carriers; 321 (49%) are primary active transport proteins; 7 (1%) are group translocators; 9 (1%) are transmembrane electron flow carriers; 4 (0.6%) are auxiliary transport proteins, and 20 (3%) are of unknown mechanism of action. Thus, primary and secondary active transporters are of about equal importance in Sco while other defined types of transporters are much less important.

**Table 1:** Numbers of Sco transport proteins according to TC class and subclass

**TC class**^**a**^	**Class description**	**No. of proteins**	**TC subclass**	**Subclass description**	**No. of proteins**
1	Channel/Pore	20	1.A	α-type channel	19
			1.B	β-type porin	0
			1.C	Pore-forming toxin	1
2	Secondary carrier	277	2.A	Porter (uniporter, symporter, antiporter)	277
3	Primary active transporter	321	3.A	P-P-bond hydrolysis-driven transporter	286
			3.B	Decarboxylation-driven transporter	4
			3.D	Oxidoreduction-driven transporter	28
			3.E	Light absorption-driven transporter	3
4	Group translocator	7	4.A	Phosphotransfer-driven group translocator	5
			4.B	Nicotinamide ribonucleoside uptake transporter	1
			4.C	Acyl CoA ligase-coupled transporter	1
5	Transmembrane electron carrier	9	5.A	Transmembrane 2-electron transfer carrier	8
			5.B	Transmembrane 1-electron transfer carrier	1
8	Auxiliary transport protein^b^	4	8.A	Auxiliary transport protein	4
9	Poorly defined system	20	9.A	Recognized transporter of unknown biochemical mechanism	20
Total		658			

Detailed class and subclass descriptions can be found at http://www.tcdb.org.

^a^ Transporter classes 6 and 7 have not been assigned in the TC system yet and therefore are not listed here.

^b^ Auxiliary proteins facilitate transport via established transport systems and therefore are not counted as separate systems.

Of the channel type proteins, almost all are alpha-type channels (Subclass 1.A), presumably in the cytoplasmic membrane. No outer membrane porins (Subclass 1.B) were identified, probably because actinobacteria have porins that differ from those in Gram-negative bacteria, and few of these have been characterized [21-25]. Those known for Mycobacteria, Nocardia and Corynebacteria do not have homologues in *Streptomyces* that are sufficiently similar to be recognized. A single putative channel-forming toxin (Subclass 1.C) (belonging to the BAPA Family; TCID number 1.C.42.1.1) was detected.

Secondary carriers (Subclass 2.A) and primary active transporters (mostly ATP-dependent (Subclass 3.A)) represent the majority of the transporters, but a smaller percentage are decarboxylation driven (Subclass 3.B) or oxidoreduction driven (Subclass 3.D) primary active transporters. Among the seven group translocation proteins, five belong to the phosphotransferase system (Subclass 4.A), one may be a nicotinamide ribonucleoside uptake system (Subclass 4.B), and another may be an acyl CoA ligase-coupled transporter (Subclass 4.C). Nine proteins possibly function as transmembrane electron flow carriers with eight of them carrying electron pairs (Subclass 5.A), while one may be a single electron carrier (Subclass 5.B).

#### ***Substrates transported by Sco***

**Figure 2:**
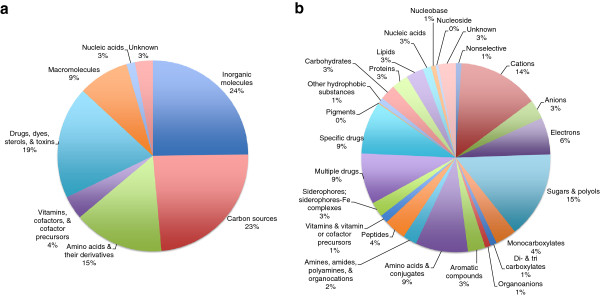
***Streptomyces coelicolor *transported substrate types.** Types of substrates transported in *Streptomyces coelicolor* by class **a)** and subclass **b)**.

Table 2 presents numbers of transport proteins in Sco categorized according to substrate. Transporters that function with inorganic molecules as substrates can be nonselective or can exhibit selectivity toward cations or anions. Almost all nonselective transporters are channels (see Additional file 1: Table S1 and Figure 2). A large majority of cation transporters (13.9% -- 89 total) are either primary active transporters (33 proteins) or secondary carriers (32 proteins). However, the remaining cation transporters are either channels (9 proteins) or poorly defined systems (15 proteins). Of the inorganic anion transporters (3.3% -- 21 total), 15 are secondary carriers and 6 are primary active transporters. Finally, for the electron transfer carriers (6.3% -- 40 total), a majority function as primary active ion pumps (29 proteins), while a smaller number of these systems are transmembrane electron flow carriers (9 proteins).

**Table 2:** Counts of Sco transport proteins according to substrate type

**Substrate**		**No. of proteins of indicated type acting on substrate type**							
		**Channels/Pores**	**Primary Carriers**	**Secondary Carriers**	**Group translocators**	**Transmembrane electron flow carriers**	**Auxiliary proteins**	**(Putative) Poorly characterized**	**Total no. of systems**
**I. Inorganic Molecules**									
A.	Nonselective	5		1					6
B.	Cations	9	33	32				15	89
C.	Anions		6	15					21
D.	Electrons		29	2		9			40
**II. Carbon sources**									
A.	Sugars & polyols	2	83	9	2				96
B.	Monocarboxylates		11	15					26
C.	Di- & tricarboxylates			7					7
D.	Organoanions (noncarboxylic)		2	6					8
E.	Aromatic Compounds			8					8
**III. Amino acids & their derivatives**									
A.	Amino acids & conjugates	1	16	39					56
B.	Amines, amides, polyamines, & organocations	1	5	7	2				15
C.	Peptides		20	1					21
**IV. Vitamins, cofactors & cofactor precursors**									
A.	Vitamins & vitamin or cofactor precursors		5	3	1				9
B.	Enzyme & redox cofactors								0
C.	Siderophores; siderophore-Fe complexes		21	8					29
**V. Drugs, dyes, sterols & toxins**									
A.	Multiple drugs		20	36					56
B.	Specific drugs		4	58					62
C.	Pigments		7	1					8
D.	Other hydrophobic substances		6						6
**VI. Macromolecules**									
A.	Carbohydrates	1	16				1		18
B.	Proteins	1	10				3	3	17
C.	Lipids		14	7	1				22
**VII. Nucleic acids**									
A.	Nucleic acids		10	8	1			2	21
**VIII. Unknown**									
A.	Unknown		3	14					17
**Total**		20	321	277	7	9	4	20	658

Substrate categories include: (I) inorganic molecules; (II) carbon sources; (III) amino acids & their derivatives; (IV) vitamins, cofactors & cofactor precursors; (V) drugs, dyes, sterols & toxins; (VI) macromolecules; (VII) nucleic acids; and (VIII) unknown.

Of the carbon sources taken up by Sco, we find that the types of transporters used correlate with the type of energy generated by metabolism of these compounds. Thus, sugars & polyols (14.8% -- 96 total), normally metabolized via glycolysis, are transported largely by primary active ABC-type transporters (83 proteins). Since these ATP-dependent porters usually exhibit higher affinities than secondary carriers, this suggests that sugars may be present in the soil environments of *Streptomyces* species at low concentrations. However, 9 secondary carriers, 2 channels, and 2 group translocators are specific for these molecules. PTS group translocators, like ABC transporters, are usually high affinity systems that recognize their sugar substrates with micromolar or sub-micromolar affinities. Since they use phosphoenolpyruvate to energize uptake, the same arguments presented for ABC transporters apply.

Monocarboxylates (3.6% - 23 total) are transported by 15 secondary carriers and 11 primary active transporters. Di- & tricarboxylates and aromatic compounds are transported solely by secondary carriers while noncarboxylic organoanions are mostly transported by secondary carriers. In summary, sugars are transported primarily by ATP-driven porters, while organic anionic compounds are transported primarily by pmf-driven carriers. This observation is in agreement with the primary energy source generated by the metabolism of these compounds (ATP from sugars; the pmf from organic acids).

Amino acids & their derivatives are transported primarily by secondary carriers although peptides are taken up almost exclusively by ABC systems. Transporters for amino acids and conjugates (9% - 56 total) include secondary carriers (39 proteins), primary active transporters (16 proteins), and a single channel. Amines, amides, polyamines & organocations (2.4% - 15 total) were found to be transported by both primary active transporters (5 proteins) and secondary carriers (7 proteins). They are also transported by two amino sugar uptake group translocators (both TC# 4.A.1.1.5) and a channel protein (TC# 1.A.11.1.3). With the exception of one secondary carrier (TC# 2.A.17.1.1), almost all peptides (3.8% - 21 total) are taken up or expelled by primary active transporters (20 proteins). Considered collectively, nitrogenous compounds are thus transported roughly equally by primary and secondary carriers.

Vitamins and especially iron siderophore complexes are primarily taken up via ABC-type active transporters. Specifically, vitamins & vitamin or cofactor precursors are taken up by primary active transporters (5 proteins), secondary carriers (3 proteins) and a single group translocator. Transporters for siderophores and siderophore-Fe complexes (29 total) are mostly primary active transporters (21 proteins), with fewer secondary carriers (8 proteins). This fact probably reflects the need for high affinity recognition due to the low concentrations of these substances in the external environment.

Transport of drugs and other hydrophobic substances occurs primarily by secondary pumps. Systems for multiple drugs (8.7% - 56 total) are exported via secondary carriers (36 proteins) and primary active transporters (20 proteins), but almost all of the specific drug exporters (62 total) are secondary carriers (58 proteins), with only four exceptional primary active transporters. By contrast, of the 8 pigment exporters identified [26,27], 7 proved to be primary carriers. All other systems specific for hydrophobic substances are primary active transporters.

Macromolecular transporters can be specific for complex carbohydrates, proteins, nucleic acids or lipids. Almost all systems specific for complex carbohydrates (2.7% - 18 total) are primary active transporters, and more than half of the protein and ligand secretion systems are primary active transporters. Nucleic acid precursor transporters fall into several classes and subclasses, with about equal numbers of primary and secondary carriers.

#### ***Superfamily representation in Sco***

Examination of the superfamilies represented in Sco revealed that of the transmembrane proteins, the largest proportion of these proteins falls into the ABC Functional Superfamily (39% - 249 proteins), which includes three independently evolving families of integral membrane proteins [28]. The Major Facilitator Superfamily (MFS) of secondary carriers (18% - 114 proteins) is the second most represented superfamily. The next largest superfamily is the APC Superfamily, which includes 6% of the transmembrane porters (32 proteins). The RND and DMT superfamilies (16 and 17 proteins respectively) both contain about 3% of the transporters, while the P-ATPase, CDF, and CPA superfamilies each encompass roughly 2%. Additional superfamilies that each encompass approximately 1% of the porters include the VIC, BART, IT, PTS-GFL, and COX Superfamilies (see TCDB for further explanation).

#### ***Topological analyses of Sco transporters***

**Figure 3:**
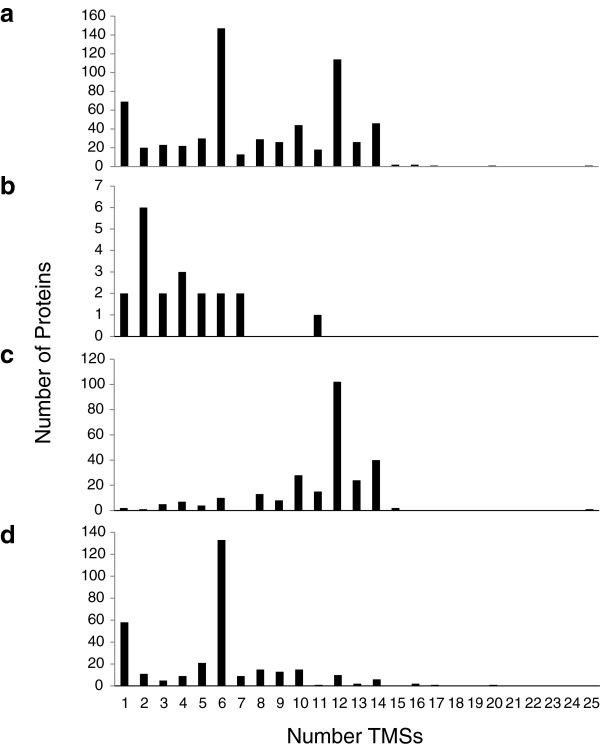
***Streptomyces coelicolor *transport protein topologies.** Transport protein topologies for all proteins **a)**, channels **b)**, secondary carriers **c)** and primary active transporters **d)** in *Streptomyces coelicolor*.

Sco transport proteins were examined according to predicted topology (Figure 3). The topologies of all proteins included in our study are presented in Figure 3a. Except for the 1 transmembrane segment (TMS) proteins (largely ABC-type extracytoplasmic solute receptors with a single N-terminal signal TMS), proteins with even numbers of TMSs outnumber proteins with odd numbers of TMSs, with the 6 and 12 TMS proteins predominating. For the few channel proteins (Class 1), 2 and 4 TMS proteins are most numerous, but for carriers (Class 2; primarily MFS carriers) and primary active transporters (Class 3; primarily ABC porters), 12 and 6 TMS proteins predominate, respectively. These are equivalent considering that MFS permeases are functionally monomeric while ABC systems are most frequently dimeric. The evolutionary explanations for these topological observations in transporters have been discussed previously [29].

#### ***Distribution of transport protein genes within the Sco genome***

Bentley *et al*. [11] reported that the *S. coelicolor* genome is divided into three parts: arm1 (~0 - 1.5 Mbp), arm2 (~6.4 - 8.67 Mbp), and the core (~1.5 - 6.4 Mbp). We therefore examined these three segments of the chromosome to see if the transport protein-encoding genes for any of the well represented (sub)families tended to localize to one of these regions. The results are presented in Additional file 2: Table S2. In general, the proteins of any one (sub)family are distributed fairly equally between these three segments with few exceptions.

Arm1 includes 17% of the total chromosome and encodes 16% of the transport proteins. The core includes 57% of the chromosome and encodes 54% of the transport proteins. Arm2 includes 26% of the chromosome and encodes 30% of the transport proteins. Thus, transporter genes exhibit nearly uniform density within the three chromosomal segments.

Three (sub)families (2.A.1.67, 2.A.39 and 3.A.1.3) have five members in *S. coelicolor*. The distributions of the encoding genes within arm1, arm2, and core are 0/1/4, 1/2/2 and 0/0/5. Subfamily 3.A.1.3 is concerned exclusively with the uptake of polar amino acids and therefore probably serves housekeeping functions. Five subfamilies have six proteins, and all but one are represented in all three chromosomal segments. Two subfamilies have seven proteins and two have eight. All four are also represented in all three segments. Two subfamilies (3.A.1.2 and 3.A.1.105) have ten members, and while the former has representation in all three segments, the latter has all ten genes in the core. These proteins catalyze drug export. Subfamily 2.A.1.2 has eleven members distributed throughout the chromosome. Two (sub)families have seventeen members. Family 2.A.3 amino acid uptake porters and subfamily 3.A.1.5 peptide and oligosaccharide uptake systems are distributed about equally on arm2 and the core with little or no representation on arm1. Finally, the 45 members of the MFS polar amino acid porters (subfamily 2.A.1.3) show equal representation in arm 2 and the core, but poor representation in arm1. Conversely, ABC sugar transporters of subfamily of 3.A.1.1 with 75 members have nearly equal distribution in the three chromosomal segments. In this case the gene density is somewhat highest on arm1.

These results show that while the transporters in general are distributed in accordance with expectation based on the sizes of these segments, some (sub)families are asymmetrically distributed. However, seldom are the members of a single (sub)family localized to a single segment.

#### ***Identification of distant transport proteins in Sco***

In the analyses reported above, the cutoff point for proteins retrieved using the GBLAST program was an e-value of 0.001. In order to determine if more distant transport protein homologues could be identified, all sequences brought up with e-values between 0.001 and 0.1 were examined. In Sco, over 300 sequences were retrieved, almost all of which proved to be false positives. However, careful examination revealed that a few true transport protein homologues were included in this list. The following 14 proteins, all of which have been included in TCDB, were obtained (see Table 3).

**Table 3:** Distant Sco transport proteins

**Assigned TC number**	**UniProt acc number**	**Size (number of aas)**	**Number of TMSs**	**Family assignment**
2.A.1.21.18	Q9KXM8	463	12	MFS Superfamily
2.A.1.21.19	Q9KYD4	411	12	MFS Superfamily
2.A.4.8.1	Q9X897	234	6	CDF Family
2.A.7.3.43	O86513	334	9	DMT Superfamily
2.A.16.4.6	Q9KY69	338	10	TDT Family
2.A.66.11.1	Q9RJJ1	429	12	MOP Superfamily
2.A.85.10.1	Q9K4J6	752	12	ArAE Family
2.A.85.10.2	Q9AJZ2	753	9	ArAE Family
8.A.3.4.1	Q9KYG0	239	2	MPA1-C Family
9.A.31.1.2	Q9XA27	436	10	SdpAB Family
9.B.36.1.2	Q9AK72	226	6	Hde Family
9.B.74.4.1	Q9K3K9	357	6	PIP Family
9.B.140.1.1	Q9K4J8	280	6	DUF1206 Family

Proteins were retrieved with GBLAST e-values between 0.1 and 0.001, individually verified and assigned TC numbers as indicated.

Two proteins (Q9KXM8 and Q9KYD4) were 12 TMS proteins that proved to be members of the Drug:H^+^ Antiporter-3 (DHA3) Family within the Major Facilitator Superfamily (MFS). These 2 proteins were assigned TC numbers 2.A.1.21.18 and 2.A.1.21.19. A third protein proved to belong to the Cation Diffusion Facilitator (CDF) Family. This protein (Q9X897; 234 aas; 6 TMSs) was assigned to a new CDF Subfamily, TC# 2.A.4.8.1. A homologue (Q9RD35; 238 aas; 6 TMSs) was so similar to its paralogue, Q9X897 (83 % identity and 90% similarity with 1 gap), that it was not entered into TCDB. A fifth protein (O86513; 334 aas; 9TMSs) proved to belong to the Drug Metabolite Exporter (DME) Family within the Drug Metabolite Transporter (DMT) Superfamily and was assigned TC# 2.A.7.3.43. A sixth protein (Q9KY69; 338 aas; 10 TMSs) was shown to belong to the Telurite-resistance/Dicarboxylate Transporter (TDT) Family and was assigned TC# 2.A.16.4.6. Finally, a seventh protein (Q9RJJ1; 429 aas; 12 TMSs) defined a new family within the Multi-drug Oligosaccharide-lipid/Polysaccharide (MOP) Flippase Superfamily, and this protein was assigned TC# 2.A.66.11.1.

A single protein (Q9KYG0; 239 aas; 2 TMSs) was found that showed low sequence similarity with an auxilary transport protein found within TC category 8. It belongs to the Membrane-Periplasmic Auxilary-1 (MPA1) Protein with Cytoplasmic (C) Domain (MPA1-C or MPA1+ C) Family of complex carbohydrate exporters [30,31]. Proteins of this family function in conjunction with members of the Polysaccharide Transport (PST) Family (TC# 2.A.66.2) within the MOP Superfamily. It is not known if this auxiliary protein functions together with the MOP Superfamily homologue, 2.A.66.11.1. However, it was encoded by a gene that is adjacent to a glycosyl transferase and a polysaccharide deacetylase, suggesting a role in polysaccharide export. Q9KYG0 was assigned TC# 8.A.3.4.1.

Five additional proteins were identified that are homologues of proteins currently listed in TC Class 9 (putative transporters of unknown mechanism of action). The first of these, a YvaB homologue (Q9XA27; assigned TC# 9.A.31.1.2; 10 TMSs and 436 aas), is a distantly related member of the SdpC Peptide Antibiotic-like Killing Factor exporter (SdpAB) Family [32]. Members of this family had been previously identified only in species closely related to bacilli. Although an SdpC homologue was not identified in *S. coelicolor*, homologues were identified in other *Streptomyces* species.

The second Class 9 protein identified in Sco was a 6 TMS homologue (Q9AK72; 6 TMSs; 226 aas), a member of the Acid Resistance Membrane Protein (HdeD) Family. It was assigned TC# 9.B.36.1.2, but no functional assignment was possible. The third Class 9 protein (Q9K3K9; 357 aas; 6 TMSs) was assigned TC# 9.B.74.4.1. It belongs to the Phage Infection Protein (PIP) Family. Homologues include putative transport proteins of the ABC-2 Superfamily. The fourth protein (Q9K4J8; 280 aas; 6 TMSs) was assigned TC # 9.B.140.1.1, a member of a novel TC family. This protein belongs to the DUF1206 Family. Finally, the fifth Class 9 protein (Q9X9U1; 513aas; 6 TMSs) was assigned TC# 9.B.141.1.1 and belongs to the YibE/F Family.

### ***Myxococcus xanthus *** **Transporters**

**Figure 4:**
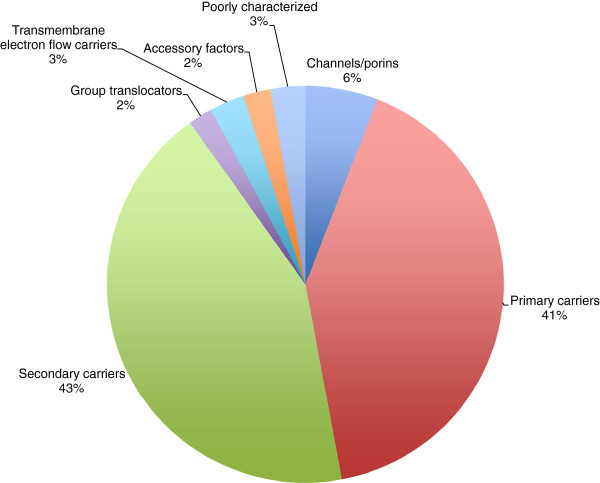
***Myxococcus xanthus *transporter type percentages.** Transporter type percentages in *Myxococcus xanthus*, based on the Transporter Classification (TC) system.

Additional file 3: Table S3 and Figure 4 present an overall summary of the classes and subclasses of transporters found in *Myxococcus xanthus* (Mxa) according to TC number. We identified 355 integral membrane transport proteins encoded in the Mxa genome. The entire genome is 9.14 million base pairs and encodes 7,316 proteins. Thus, 4.8% of the proteins encoded within the genome of Mxa are recognized transmembrane transport proteins. This value does not include transport accessory proteins such as cytoplasmic ATPases and extracytoplasmic receptors.

#### ***Types of transporters in Mxa***

Mxa encodes all of the major types of transport proteins represented in TCDB (see Table 4). 21 (5.9%) of these proteins are simple channels, 153 (43.1%) are secondary carriers, 146 (41.1%) are primary active transport proteins, 7 (2%) are likely to be group translocators, 10 (2.8%) are transmembrane electron flow carriers, 8 (2.3%) are auxiliary transport proteins, and 10 (2.8%) are of unknown mechanism of action. It therefore appears that in Mxa, similar to Sco, primary and secondary active transporters are of about equal importance, while other defined types of transporters are of much lesser importance.

**Table 4:** Numbers of Mxa transport proteins according to TC class and subclass

**TC class**^**a**^	**Class description**	**No. of proteins**	**TC subclass**	**Subclass description**	**No. of proteins**
1	Channel/Pore	21	1.A	α-type channel	18
			1.B	β-type porin	3
2	Secondary carrier	153	2.A	Porter (uniporter, symporter, antiporter)	153
3	Primary active transporter	146	3.A	P-P-bond hydrolysis-driven transporter	124
			3.B	Decarboxylation-driven transporter	4
			3.D	Oxidoreduction-driven transporter	18
4	Group translocator	7	4.A	Phosphotransfer-driven group translocator	2
			4.C	Acyl CoA ligase-coupled transporter	5
5	Transmembrane electron carrier	10	5.A	Transmembrane 2-electron transfer carrier	10
8	Auxiliary transport protein^b^	8	8.A	Auxiliary transport protein	8
9	Poorly defined system	10	9.A	Recognized transporter of unknown biochemical mechanism	10
Total		355			355

Detailed class and subclass descriptions can be found at http://www.tcdb.org.

^a^ Transporter classes 6 and 7 have not been assigned in the TC system yet and therefore are not listed here.

^b^ Auxiliary proteins facilitate transport via established transport systems and therefore are not counted as a separate system.

Of the channel proteins, almost all are alpha-type channels (Subclass 1.A). A few outer membrane porins (Subclass 1.B) were identified, but these were not examined more closely because of the recent extensive studies of Bhat et al. [33]. No potential channel-forming toxins (Subclass 1.C) were detected. The secondary carriers include mostly symporters (importers) and antiporters (exporters), while almost all primary active transporters are ATP-dependent (Subclass 3.A). However, a smaller percentage may be oxidoreduction driven (Subclass 3.D) or decarboxylation driven (Subclass 3.B). Among the seven group translocation proteins, two belong to the phosphotransferase system (Subclass 4.A), while five may be acyl CoA ligase-coupled transport systems (Subclass 4.C). Of the ten proteins possibly functioning as transmembrane electron flow carriers, all ten are likely to carry an electron pair (Subclass 5.A). None is likely to be a single electron carrier (Subclass 5.B). Eight auxiliary transport proteins (Subclass 8.A) and ten recognized transporters of unknown mechanism of action (Subclass 9.A) were also identified.

#### ***Substrates transported by Mxa***

**Figure 5:**
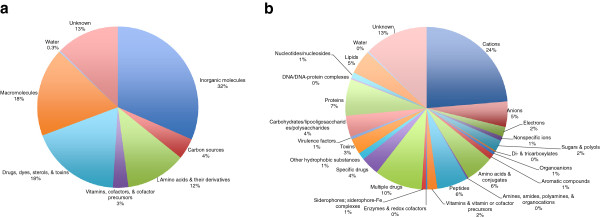
***Myxococcus xanthus *transported substrate types.** Types of substrates transported in *Myxococcus xanthus* by class **a)** and subclass **b)**.

Table 5 and Figure 5 show numbers of transport proteins in Sco organized according to substrate types. Transporters that utilize inorganic molecules as substrates make up a large portion of all transport proteins found in Mxa. Cation-specific transporters (23.7% -- 84 total) are split evenly between primary and secondary carrier systems (36 and 38 proteins, respectively) with only six recognized channels. There are markedly fewer inorganic anion transporters (5.1% -- 18 total), including 6 primary carriers and 10 secondary carriers. In comparison, there are relatively few electron transport systems in Mxa.

**Table 5:** Counts of Mxa transport proteins according to substrate type

**Substrate**		**No. of proteins of indicated type acting on substrate type**							
		**Channels/Pores**	**Primary carriers**	**Secondary carriers**	**Group translocators**	**Transmembrane electron flow carriers**	**Auxiliary proteins**	**(Putative) Poorly characterized**	**Total no. of systems**
**I. Inorganic molecules**									
A.	Nonselective	3							3
B.	Cations	6	36	38		1		3	84
C.	Anions		6	10		2			18
D.	Electrons		4			3			7
**II. Carbon sources**									
A.	Sugars & polyols		4	2	2				8
B.	Monocarboxylates								0
C.	Di- & tricarboxylates			1					1
D.	Organoanions (noncarboxylic)			2					2
E.	Aromatic Compounds		4						4
**III. Amino acids & their derivatives**									
A.	Amino acids & conjugates		6	14					20
B.	Amines, amides, polyamines, & organocations	1							1
C.	Peptides		12	9				1	22
**IV. Vitamins, cofactors & cofactor precursors**									
A.	Vitamins & vitamin or cofactor precursors		5	2					7
B.	Enzyme & redox cofactors		1						1
C.	Siderophores; siderophore-Fe complexes		2	1					3
D.	Nucleosides/nucleotides	1	2	1					4
**V. Drugs, dyes, sterols & toxins**									
A.	Multiple drugs		1	32			1		34
B.	Specific drugs		11	2					13
C.	Pigments								
D.	Other hydrophobic substances			5					5
E.	Toxins	6	4	1					11
F.	Virulence factors							2	2
**VI. Macromolecules**									
A.	Carbohydrates	1	2	9			3		15
B.	Proteins	2	19	1			4		26
C.	Lipids		9	3	5				17
**VII. Nucleic acids**									
A.	Nucleic acids		1						1
								
**VIII. Water**									
A.	Water	1							1
**IX. Unknown**									
A.	Unknown		17	20		4		4	45
**Total**		21	146	153	7	10	8	10	355

Substrate categories include: (I) inorganic molecules; (II) carbon sources; (III) amino acids & their derivatives; (IV) vitamins, cofactors & cofactor precursors; (V) drugs, dyes, sterols & toxins; (VI) macromolecules; (VII) nucleic acids; and (VIII) unknown.

Carbon compounds are transported by relatively few systems in Mxa. Sugars and polyols (2.3% -- eight total) are taken up by a combination of primary carriers (four proteins), secondary carriers (two proteins), and group transolcators (two proteins). A single secondary carrier is responsible for di- and tricarboxylate transport, while two secondary carriers are involved in organoanion transport. Aromatic compounds are transported by four primary carriers. As a predatory bacterium, the lack of a wide variety of transporters with carbon based substrates in Mxa can possibly be due to a greater reliance on amine-based derivatives for sustenance; Bretscher and Kaiser showed that many mono- and disaccharides were not among the minimal medium requirements for vegetative growth of Mxa colonies [34].

Amino acids and their derivatives are transported by a much greater variety of transporters. Amino acids and their conjugates (5.6% -- 20 total) are transported primarily by secondary carriers (14 proteins), with approximately half as many primary carriers (six proteins). A single channel functions in amine, amide, polyamine and organocation transport. Peptides (5.9% -- 21 total) are taken up or expelled via 12 primary carriers and nine secondary carriers. Thus, relative to transporters specific for saccharide-based substrates, the high number of transporters for amine-based substrates indicates that Mxa uses amino acids and their derivatives as its main sources of carbon, an observation that has also been suggested in other studies [12].

Vitamins and other cofactor precursors (2.0% -- seven total) are taken up more by primary active transporters than by secondary carriers. Two primary carriers and a single secondary carrier may be involved in siderophore/siderophore-iron complex transport. Nucleosides/nucleotides are transported by one channel, one secondary carrier, and two primary active transporters.

Transporters for drugs, toxins and other hydrophobic substances are primarily secondary carriers. Systems capable of exporting multiple drugs (9.6% -- 34 total) are almost exclusively secondary carriers (32 proteins). No Mxa transporter specific for pigments was identified, but transporters specific for toxins and other hydrophobic substances proved also to be secondary carriers.

Macromolecular exporters transporting complex carbohydrates, proteins and lipids were identified. Of the carbohydrate transporters, two are primary active transporters and nine are secondary carriers. Almost all protein exporters are primary carriers. A total of 17 systems (4.8%) were found to transport lipids, mostly by primary carriers, although a few secondary carriers and potential group translocators were also identified. The expanded diversity of protein transport systems is probably a reflection of the tracking and microbial killing mechanisms used by Mxa, which secretes hydrolytic enzymes and secondary metabolites with antimicrobial activities [35].

#### **Topological analyses of Mxa *transporters***

**Figure 6:**
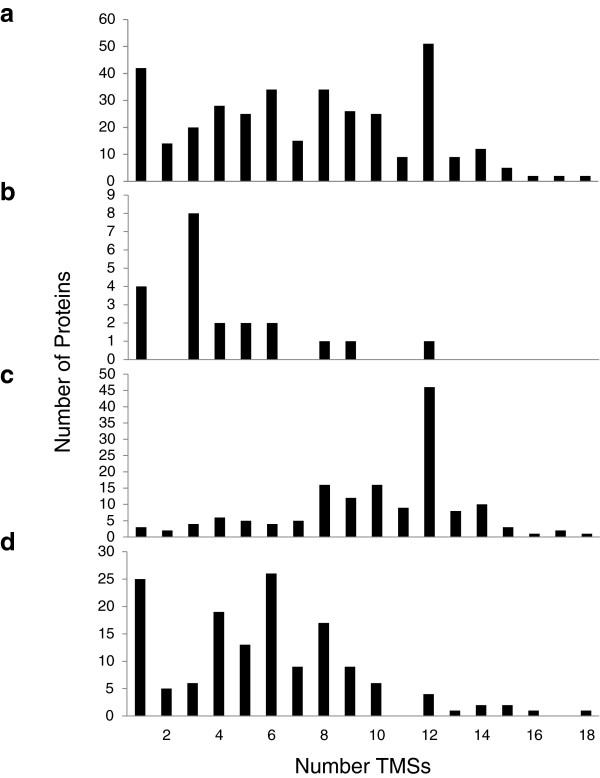
***Myxococcus xanthus *transport protein topologies.** Transport protein topologies for all **a)** proteins, **b)** channels, **c)** secondary carriers, and **d)** primary active transporters in *Myxococcus xanthus.*

We analyzed the predicted topologies of all retrieved Mxa transport proteins (Figure 6a). For the most part, proteins with even numbers of TMSs outnumber proteins with odd numbers of TMSs, with notable discrepancies in channel proteins (Subclasses 1.A and 1.B) and active transporters. Single TMS primary active transport proteins are mostly ABC extracytoplasmic solute receptors with one N-terminal signal TMS, while the high number of 3 TMS proteins in 1.B is due to eight members of the Mot-Exb Superfamily, involved in motility as well as outer membrane transport. Among transporters with even numbered TMSs, 6 and 12 TMS proteins are most numerous, encompassing members of the ABC Superfamily and the MFS, respectively.

#### ***Identification of distant transport proteins in Mxa***

To identify distant transport protein homologues in Mxa, the same procedure was used as for Sco. In Mxa, over 130 sequences were retrieved with values between 0.001 and 0.1. Similarly to Sco, most proved to be false positives with only 8 proving to be true homologues of existing TC entries; all 8 have been entered into TCDB (see Table 6).

**Table 6:** Distant Mxa transport proteins

**Assigned TC #**	**UniProt acc #**	**Size (# aas)**	**# TMSs**	**Family assignment**
2.A.1.15.16	Q1DA07	731	13	MFS Superfamily
2.A.7.31.1	Q1DCP3	290	10	DMT Superfamily
2.A.37.6.1	Q1D5P4	432	14	CPA2 Family
2.A.66.12.1	Q1D7B4	506	14	MOP Superfamily
3.A.1.144.3	Q1D0V1	266	6	ABC Superfamily
3.A.1.145.1	Q1D520	1200	13	ABC Superfamily
9.B.139.2.1	Q1CXZ2	211	3	SdpC Family
9.B.104.6.1	Q1D006	242	7	Rhomboid Family

Proteins were retrieved with GBLAST e-values between 0.1 and 0.001, individually verified and assigned TC numbers as indicated.

A single protein (Q1D5P4; 432 aas; 14 TMSs) proved to be a member of the Monovalent Cation:Proton Antiporter-2 (CPA2) Family, and it was assigned TC# 2.A.37.6.1 in a novel subfamily. It could be a K^+^:H^+^ or Na^+^:H^+^ antiporter. A second protein (Q1DCP3; 290 aas; 10 TMSs) was shown to be a member of the Drug/Metabolite Transporter (DMT) Superfamily, distantly related to members of the Drug Metabolite Exporter (DME) Family. It was assigned TC # 2.A.7.31.1, also in a novel subfamily. A third protein (Q1D7B4; 506 aas; 14 TMSs) was assigned TC# 2.A.66.12.1 as a member of the Multidrug/Oligosaccharidyl-lipid/Polysaccharide (MOP) Flippase Superfamily. It belongs to a family within this superfamily for which no functional data are available. A fourth protein (Q1DA07; 731 aas; 13 TMSs) belongs to the Major Facilitator Superfamily (MFS) and was assigned TC# 2.A.1.15.16. The gene of this protein is adjacent to a putative S-adenosyl methionine (SAM)-dependent methyltransferase whose homologues include puromycin methyltransferases. The substrate of this protein is potentially a drug that undergoes modification by methylation for detoxification purposes.

Two proteins proved to be members of the ABC-2 Superfamily within the ATP-binding Cassette (ABC) Functional Superfamily [28]. One protein (Q1D520; 1200 aas; 13 TMSs) was assigned to a new ABC family with TC# 3.A.1.145.1. Notably, this exporter proved to be a fusion between an N-terminal ABC-2 domain with 13 putative TMSs and a hydrophilic C-terminal zinc dependent amino peptidase domain (Peptidase M1 Family), suggesting that the transporter domain could be involved in the export of an amino acid, amino acid derivative, or product of amino acid metabolism. In addition, Q1D520 resembles (35.4% identity and 54.6% similarity with 4 gaps) 3.A.1.145.3, another ABC-2 export permease fusion protein annotated as being involved in multi-copper enzyme maturation. The other ABC protein (Q1D0V1; 266 aas; 6 TMSs) was assigned TC# 3.A.1.144.3 and is functionally uncharacterized.

Two proteins were shown to be homologous to proteins in TC Category 9. The first protein (Q1CXZ2; 211 aas; 3 TMSs) was found to be a member of the Cannabalism Toxin SdpC (SdpC) Family and was assigned TC# 9.B.139.2.1. The second protein (Q1D006; 242 aas; 7 TMSs) was assigned TC# 9.B.104.6.1. It belongs to the Rhomboid Protease Family and shows sequence similarity to members of the MFS; this result provides preliminary evidence that the MFS and Rhomboid Protease Family may in fact be homologous and warrants future investigation.

### **Comparison of *Streptomyces coelicolor *** **(Sco) with *Myxococcus xanthus *** **(Mxa)**

As noted above, the genomes of Sco and Mxa are nearly the same size (~9 Mbps), but the numbers of reported proteins in the proteomes differ substantially (8153 versus 7316 proteins, respectively; about 10% less for Mxa) [11,12,36]; See Discussion for an explanation. Moreover, using the same setting (cut-off of 0.001 representing values giving fairly reliably related homologues) for G-BLAST searches of the two genomes, the numbers of integral membrane transport protein hits were dramatically different (658 for Sco versus 355 for Mxa). It is possible that some of these differences reflect the criteria used for protein identification used by the annotators of the genome sequences of these two organisms. However, as noted below, these differences, particularly with respect to the numbers of transporters reported in Tables 1 and 4, are likely to reflect fundamental differences between the two organisms. It is also possible, although unlikely, that these differences, in part, represent greater sequence divergence of Mxa transporters compared to Sco transporters relative to the existing proteins in TCDB at the time when these analyses were conducted. As a result, we could have missed transporters too divergent in sequence to be detected with the selected cut-off value. Because analyses of distant transport homologues of Sco and Mxa were performed, this possibility seems unlikely. Instead, Sco appears to have greatly amplified the numbers of certain types of transporters. The following comparisons and descriptions are pertinent to homologues obtained with scores smaller than (better than) the 0.001 threshold.

### Channel proteins

The largest superfamily of channel proteins found in nature is the Voltage-gated Ion Channel (VIC) Superfamily (TC# 1.A.1-5 and 10) [37,38]. While Sco has six VIC family (1.A.1) members, Mxa has only one, and neither organism shows representation in the other families of the VIC Superfamily see superfamily hyperlink in TCDB; [39].

All of the hits in both organisms gave values sufficient to establish homology, but no two VIC family homologues in these two dissimilar organisms proved most similar to the same TC entry. Thus, in Sco, one protein most resembles the well-characterized 2 TMS KcsA K^+^ channel of *S. lividans*[40], but no such homologue was identified in Mxa. Instead, the one VIC family member in Mxa is a 6 TMS K^+^ channel resembling bacterial 6 TMS homologues (TC 1.A.1.24). Other VIC family members in Sco include 2 and 4 TMS VIC family homologues, sometimes with extra C-terminal TrkA-N Rossman NAD-binding domains that presumably function in regulation of channel activity. These novel proteins have been entered into TCDB.

Both Sco and Mxa have two MIP family aquaporins/glycerol facilitators [41]. These four proteins hit different TC entries with good scores (≤e^-34^), demonstrating that they are indeed members of the MIP family. They probably allow the passive flow of water and small neutral molecules such as glycerol across the bacterial plasma membranes. Sco also has a simple anion channel of the CLC Family (1.A.11) that is lacking in Mxa.

Mechanosensitive channels include MscL and MscS proteins, both involved in osmotic adaptation, acting as emergency release valves [42]. Only Sco has an MscL channel (1.A.22), but both organisms have four MscS proteins, some of which are similar between the two organisms. For example, Sco Q9S2Y1 and Mxa Q1D0J8 are 39% identical throughout most of their lengths and have therefore been assigned TC#s 1.A.23.7.1 and 1.A.23.7.2, respectively. Moreover, both Sco Q86576 and Sco Q9L1X9 show >33% identity throughout major portions of their sequences with Mxa Q1DEP9.

Mxa has eight proteins belonging to the multicomponent Mot-Exb Family (1.A.30) of H^+^ or Na^+^ channel chemiosmotic energizers used for motility and/or outer membrane transport. Sco, being a Gram-positive organism, lacks these homologues. Since it lacks flagellar motility, Mxa lacks MotA/MotB as expected, but it has several TolQ/TolR energizers for transport across the outer membrane [43]. In most cases, both TolQ and TolR were identified, although only TolQ homologues are listed in Table 2. These protein pairs have been entered into TCDB under TC#s 1.A.30.2.3 - 1.A.30.2.7.

Two other systems specific to Gram-negative bacteria but lacking in Gram-positive bacteria are the Outer Membrane Protein Insertion Porin (Bam or OmpIP) Family (1.B.33) [44,45] and the Outer Membrane Lipopolysaccharide Export Porin (LPS-EP) Family (1.B.42) [46,47]. As expected, constituents of these two systems were identified in Mxa, but not Sco. Although only some of these constituents are listed in Table 4, homologues of the *E. coli* constituents were identified, sometimes in multiple copies. Outer membrane porins of Mxa have been examined by Bhat *et al*., [33] and were therefore not considered further here. Several of these sequence divergent proteins have been included in TCDB.

### Secondary carriers (TC Sub-class 2.A)

#### ***The major facilitator superfamily (MFS)***

The largest superfamily of secondary carriers found in nature is the MFS [48,49]. Within the MFS (2.A.1), Sco has 114 recognizable homologues, while Mxa has only 32. This huge difference accounts for a significant fraction of the total number of transporters Sco has in excess of those that Mxa has (82 of 203, or 41%). Those proteins with low scores to preexisting entries in TCDB (E-values of > e^-10^) were entered into this database, thus allowing recognition of more distantly related family members in future studies.

A summary of MFS members in Sco and Mxa is presented in Table 7. Almost no sugar transporters of the MFS are found in either Sco or Mxa. Thus, while Sco has two members of the sugar porter (SP) family (2.A.1.1), Mxa has none, and sugar transporters of the OHS (2.A.1.5), FHS (2.A.1.7), NHS (2.A.1.10), SHS (2.A.1.12), PP (2.A.1.18), SET (2.A.1.20), and GPH (2.A.2) families are not represented in either organism. As will be demonstrated below, sugar transporters in Sco belong primarily to the ABC and PTS functional superfamilies.

**Table 7:** MFS members in Sco and Mxa

**TC Number**	**Family name**	**Known substrate range**	**Sco**	**Mxa**
2.A.1.1	The Sugar Porter (SP) Family	sugar and sugar derivative (uniport; symport); urate (antiport)	2	
2.A.1.2	The Drug:H^+^ Antiporter-1 (12 Spanner) (DHA1) Family	drug, polyamine, neurotransmitter, sugar, nucleobase/side, siderophore, lipid (antiport); vitamin (symport)	12	9
2.A.1.3	The Drug:H^+^ Antiporter-2 (14 Spanner) (DHA2) Family	drug, boron, bile acid, parquot, fatty acid, siderophore, amino acid (antiport); pyrimidine (symport)	49	6
2.A.1.4	The Organophosphate:Pi Antiporter (OPA) Family	carbohydrate phosphate (antiport)		1
2.A.1.6	The Metabolite:H^+^ Symporter (MHS) Family	organic acid/base, sugar acid (symport)	6	1
2.A.1.8	The Nitrate/Nitrite Porter (NNP) family	nitrate/nitrite (symport/antiport)	2	1
2.A.1.11	The Oxalate:Formate Antiporter (OFA) Family	oxalate/formate (antiport)	3	
2.A.1.14	The Anion:Cation Symporter (ACS) Family	organic and inorganic anion, peptide, vitamin, amino acid, nucleotide (uniport; symport)	3	
2.A.1.15	The Aromatic Acid:H^+^ Symporter (AAHS) Family	aromatic acid, vitamin (symport)	3	1
2.A.1.17	The Cyanate Porter (CP) Family	cyanate, glucose (symport)	3	
2.A.1.21	The Drug:H^+^ Antiporter-3 (12 Spanner) (DHA3) Family	drug, siderophore (antiport)	6	7
2.A.1.24	The Unknown Major Facilitator-1 (UMF1) Family	unknown	1	1
2.A.1.25	The Peptide-Acetyl-Coenzyme A Transporter (PAT) Family	peptide, glycopeptide, acyl-CoA (symport)		3
2.A.1.30	The Putative Abietane Diterpenoid Transporter (ADT) Family	diterpenoid (symport)	4	
2.A.1.34	The Sensor Kinase-MFS Fusion (SK-MFS) Family	unknown	1	
2.A.1.35	The Fosmidomycin Resistance (Fsr) Family	drug (antiport)	1	
2.A.1.36	The Acriflavin-sensitivity (YnfM) Family	drug (symport)	2	1
2.A.1.40	The Purine Transporter, AzgA (AzgA) Family	purine (symport)	2	
2.A.1.49	The Endosomal Spinster (Spinster) Family	unknown		1
2.A.1.54	The Unknown (Archaeal/Bacterial) Major Facilitator-9 (UMF9) Family	unknown	1	
2.A.1.60	The Rhizopine-related MocC (MocC) Family	rhizopine	7	1
2.A.1.67	The Unidentified Major Facilitator-16 (UMF16) Family	unknown	5	
2.A.17	The Proton-dependent Oligopeptide Transporter (POT) Family	peptide, histidine, nitrate (symport; occasionally antiport)	1	2

Representation of transporters belonging to known families within the Major Facilitator Superfamily (MFS) listed according to TC number with their substrate ranges and modes of active transport indicated.

Drug exporters are prevalent in both organisms. The DHA1 Family (2.A.1.2) has 12 members in Sco and nine in Mxa, the DHA2 Family (2.A.1.3) has 49 members in Sco and six in Mxa, and the DHA3 Family (2.A.1.21) has six and seven members in these two organisms, respectively. It is clear that Sco, but not Mxa, has greatly increased its numbers of DHA2 family members, although neither did for DHA1 or DHA3 family members. The order of representation is therefore DHA2 >DHA1>DHA3 in Sco, with huge representation of DHA2 members, but DHA1 > DHA3 > DHA2 in Mxa, with much lower representation overall. These systems presumably reflect the abilities of Sco and Mxa to produce and export antimicrobial agents and to protect themselves against toxic substances produced by other soil microorganisms. However, we do not know why Sco amplified its membership in the DHA2 family but not the DHA1 or DHA3 family.

The MHS Family (2.A.1.6) includes members that transport a wide range of metabolites, particularly organic acids such as Krebs cycle intermediates. While Mxa has one such member, Sco has six. Other MFS families that may take up organic acids that are represented in Sco to a greater extent than in Mxa include the OFA (3; 0), ACS (3; 0), AAHS (3; 1) and CP (3; 0) Families. It therefore appears that Sco uses organic acids to a much greater extent than does Mxa.

Other interesting observations are: (1) Sco has four members of the poorly characterized ADT (Adietane) Family while Mxa has none; (2) Mxa has three peptide uptake systems of the AAT Family while Sco has none; (3) both organisms have nitrate:nitrite porters of the NNP Family; (4) both have members of the YnfM (acriflavin sensitivity) Family (of unknown physiological function); (5) Sco has seven members of the MocC (Rhizopine) Family while Mxa has only one, and (6) Sco has representation in the functionally uncharacterized UMF1 (one), UMF9 (one) and UMF16 (five members), while Mxa has representation (a single protein) only in the UMF1 family. Perhaps of greatest surprise is the fact that Mxa has a member of the AAA Family, members of which are usually restricted to obligatory intracellular parasites that utilize the cytoplasmic nucleotides of their hosts as energy sources [50]. The Mxa protein is a homologue (e^-41^) of a characterized NAD^+^:ADP antiporter (2.A.12.4.1) [51]. Possibly, Mxa can take up nucleotides such as NAD^+^, ATP and ADP from the medium. Since it is a “micropredator” which lyses other bacteria, the presence of nucleotides in its growth medium would not be unexpected [52] (see Discussion). Another surprise was the discovery that Mxa and other bacteria have homologues of Spinster (Spns1 and 2), intracellular organellar sphingosine-1-phosphate or sphingolipid transporters involved in immune development, lymphocyte trafficking, and necrotic and antiphagic cell death in animals [53-56]. NCBI-BLAST searches revealed that many bacteria encode these homologues in their genomes. Two of these bacterial proteins have been entered into TCDB under TC#s 2.A.1.49.7 and 8. It will be interesting to learn if the substrates of these prokaryotic transporters are the same as in eukaryotes. Sphingolipids represent a major outer membrane lipid class in some myxobacteria [57].

#### **The amino acid/polyamine/organocation (APC) *superfamily***

Eleven families currently comprise the APC Superfamily (see TCDB), and most of them (seven) are concerned with the uptake of amino acids and their derivatives [58,59]. Sco has 32 APC superfamily members while Mxa has only six. Table 8 lists the numbers of representatives of these families in Sco and Mxa. The largest family within the APC Superfamily is the APC Family, and Sco has 17 such proteins while Mxa has only two.

**Table 8:** APC family member representation in Sco and Mxa

**TC #**	**Family**	**Sco**	**Mxa**
2.A.3	Amino Acid-Polyamine-Organocation (APC) Superfamily	17	2
2.A.15	Betaine/Carnitine/Choline Transporter (BCCT) Family	1	
2.A.18	Amino Acid/Auxin Permease (AAAP) Family		
2.A.21	Solute:Sodium Symporter (SSS) Family	8	4
2.A.22	Neurotransmitter:Sodium Symporter (NSS) Family		
2.A.25	Alanine or Glycine:Cation Symporter (AGCS) Family	1	
2.A.30	Cation-Chloride Cotransporter (CCC) Family		
2.A.39	Nucleobase:Cation Symporter-1 (NCS1) Family	5	
2.A.42	Hydroxy/Aromatic Amino Acid Permease (HAAAP) Family		

Numbers of APC Superfamily proteins in Sco and Mxa are arranged by family.

The SSS Family of solute:Na^+^ symporters, a constituent member of the APC Superfamily [59], transports a wide variety of solutes. Of the eight SSS family members in Sco, five probably transport short monocarboxylic acids (acetate, lactate, pyruvate, etc.), while three probably transport sugars. Of the four hits in Mxa, two may be monocarboxylate transporters while the other two are probably non-transporting signal transduction proteins with C-terminal sensor kinase domains. Only one of them is homologous to SSS transporters in its transmembrane domain.

#### ***Heavy metal carriers***

Both Sco and Mxa have members (five and three members, respectively) of the heavy metal efflux Cation Diffusion Facilitator (CDF) Family 2.A.4; [60], but only Mxa has members (two) of the metal uptake Zinc-Iron Permease (ZIP) Family 2.A.5; [61]. Only Sco has a member of the Nramp Family of divalent cation transporters. These proteins exhibit varying specificities for heavy metals and are involved in metal ion homeostasis. Heavy metal transporters are also found in other families such as the RND Superfamily.

#### **The RND *superfamily***

The RND Superfamily 2.A.6; [62,63] is well represented in both Sco and Mxa with 16 members in Sco and 20 in Mxa. Family 1 (Heavy Metal Efflux (HME)) is prevalent in Mxa with six members (see TCDB; 2.A.6.1.7-11 and 2.A.6.3.2), but absent in Sco. Based on induction properties, one may export Zn^2+^, two may export heavy metals (one of these is induced under starvation conditions), and three may export copper [64]. Similarly, the (largely Gram-negative bacterial) Hydrophobe/Amphiphile Efflux-1 (HAE1) Family (Family 2), usually considered to be concerned with drug export, is found in Mxa (four members) but not Sco. Surprisingly, the lipooligosaccharide Nodulation Factor Exporter (NFE) Family (Family 3) is represented in both organisms, but with six members in Mxa and only one in Sco. These proteins may transport substrates resembling rhizobial nodulation factor lipooligosaccharides, which are the substrates of the only characterized member of the NFE Family [65]. Such substrates are not known to be present in myxobacteria or actinobacteria. The reason for the presence of six such homologues in Mxa is unexplained, but it suggests that this δ-proteobacterium, other myxobacteria, and possible actinobacteria may similarly use extracellular lipooligosaccharides for purposes of communication.

Both organisms have a single member of the SecDF Family (RND Family 4) as expected for large genome bacteria. This protein pair facilitates protein secretion via the general secretory system (Sec translocase; 3.A.5), by a mechanism that involves ATP-independent *pmf*-driven substrate protein translocation where SecDF transports protons down their electrochemical gradient to drive protein export [66]. Also as expected, Sco, but not Mxa, has representation (14 members) of the largely Gram-positive bacterial HAE2 Family (RND Family 5) [63]. HAE2 family homologues function to export complex lipids to the outer actinobacterial membrane [67], although some of them may catalyze the export of antimicrobial agents (see TCDB). Finally, Mxa, but not Sco, has four members of the HAE3 Family (Family 7); functional data for members of this family are available for only one member which proved to be an exporter of hopanoids, fused pentacyclic ring cholesterol-like compounds [68].

#### **The drug/*metabolite *** **transporter (DMT) superfamily**

The DMT Superfamily 2.A.7; [69] is well represented with 17 members in Sco and 13 in Mxa. These proteins fall within several DMT families. Both organisms have members of the 4 TMS Small Multidrug Resistance (SMR) Family (Family 1), but only Mxa has a member of the functionally uncharacterized 5 TMS BAT Family (Family 2). Sco and Mxa have eight and five members, respectively, of the DME Family (Family 3) that may primarily export metabolites such as amino acids. Other families within this superfamily are primarily concerned with transport of activated sugars for glycolipid and polysaccharide synthesis, but they are not represented in either Mxa or Sco.

#### ***Other secondary carriers***

Two members of the GntP Family (2.A.8) of uptake porters for gluconate and other organic acids are found in Sco but not Mxa, in agreement with a greater dependency of metabolism of the former on carbohydrates and organic acids. Sco also has single members of each of the CitMHS, LctP, BCCT and TDT families of carboxylate uptake transporters, all of which are lacking in Mxa. This observation also points to a greater dependency of Sco on organic acids as sources of nutrition.

While Sco has two YidC homologues, involved in integral membrane protein insertion in many bacteria [70], only one such homologue was found in Mxa. Interestingly, while *E. coli* has only one YidC, *Bacillus subtilis* has two, one for vegetative growth (OxaA2) and one for sporulation (SpoIIIJ) [71]. It is possible that Sco uses its two YidC homologues for these two distinct purposes, but Mxa, with a single homologue, evidently lacks such a need. It must use the same protein for integral membrane protein insertion during both vegetative growth and spore development.

Sco but not Mxa has a Ca^2+^:Cation Antiporter (CaCA Family), while Mxa, but not Sco, has a P-type Ca^2+^-ATPase. These two organisms therefore use different mechanisms to extrude Ca^2+^ from the cell cytoplasm. These differences may be important since Ca^2+^ plays roles in development and antibiotic production in both organisms [72-75]. Sco also has two phosphate transporters of the Pit family although Mxa has only one.

Both organisms have two or three members of the Na^+^:H^+^ Antiporter (NhaA) Family. Both also have multiple members of the functionally related Cation:Proton Antiporter (CPA1 and CPA2) Families (6 and 9 for Mxa and Sco, respectively). Both bacteria have five members of the CPA2 Family, but they have one and four members of the CPA1 family, respectively. Although members of these two families are within the same superfamily, they are only distantly related. The general reactions catalyzed by members of these families are similar, but most CPA1 family members transport Na^+^ while many CPA2 family members transport K^+^ (see TCDB). They are involved in pH and inorganic cation homeostasis [76]. A single multicomponent cation:H^+^ antiporter of the CPA3 Family is present in both organisms.

Both organisms have a single ArsB arsenite exporter, but only Sco has two arsenite exporters of the Arsenical Resistance-3 (ACR3) Family. Mxa and Sco have 3 and 1 members of the DASS Family, respectively. Members of this family take up both inorganic and organic anions, depending on the system. Both organisms have three paralogues of the SulP Family, which exclusively transport inorganic anions such as sulfate and bicarbonate. They also have two or three members of the Dicarboxylate/Amino Acid:Cation (Na^+^ or H^+^) Symporter (DAACS) and Bile Acid:Na^+^ Symporter (BASS) families which exclusively transport organic anions including amino acids. The two nucleobase:cation symporter families, NCS1 and NCS2, are prevalent in Sco (8 members), but appear to be lacking in Mxa.

Both Sco and Mxa have TatA and TatC homologues, the essential constituents of the Sec-independent twin arginine translocase protein secretion system [77]. However, while Sco has a 3-component system with TatA, B and C, Mxa appears to have a 2-component system with just one TatA/B homologue [78]. Many prokaryotes have either 2 or 3 component systems, but the advantages of the greater complexity of the 3-component systems are not well understood, although distinct but overlapping functions for the *E. coli* TatA and TatB paralogues are recognized [77,78].

The MOP Superfamily of multidrug/oligosaccharidyl lipid/polysaccharide exporters [79] is present in both organisms with Mxa having 7 members and Sco having 3. In Sco, one is probably a multidrug resistance pump while the other two may catalyze export of lipid-peptidoglycan precursors to the periplasm for cell wall assembly, as suggested by Ruiz [80]. *B. subtilis* has four such homologues, one of which, SpoVB, is required for spore cortex polymerization [81]. However, Fay and Dworkin [82] have conducted experiments indicating that a strain deleted for all four genes grows normally, causing some doubt about the proposed function in lipid peptidoglycan precursor export. Possibly these porters export these substrates, but the presence of functionally redundant transporters might provide the explanation for this apparent contradiction. This possibility is reinforced by the fact that members of the bacterial specific MPE Family (2.A.103), present in almost all bacteria, are known to serve this function [83]; C.C. Zhang & M.H. Saier, unpublished results. Mxa only has one such homologue, but Sco has two. Sco could use these two paralogues during vegetative growth and spore formation, respectively, although direct evidence for this proposal is not available. Mxa has two putative polysaccharide exporters of the MOP Superfamily that could be involved in polysaccharide export for social motility, fruiting body formation, stress survival, and/or biofilm formation [84].

Peptide signaling is known to be essential for normal fruiting body development in Mxa [85]. This organism has five peptide uptake porters of the OPT Family that could function both in this capacity and in nutrition. Surprisingly, Sco lacks such systems. Because Sco also uses peptide signaling [2,86], it must use alternative mechanisms of peptide communication. It is likely that it uses ABC porters and transmembrane sensor kinases for signaling since in Gram-positive bacteria, signaling peptides are usually present in very low (sub-nanomolar) concentrations [2,87].

Several families of small molecule (especially amino acid) efflux pumps are found in these sporulating bacteria. Thus, both have single AEC, RhtB, LIV-E and ThrE exporters, although only Sco has a LysE family member. Both organisms have multiple representation in the ArAE and AI-2E families: 4 and 4 members for Sco; 2 and 7 members for Mxa. While the former systems export aromatic acids, the latter transport interspecies signaling molecules such as autoinducer-2 as well as other metabolites [88].

Several other secondary carrier families are represented in Sco and Mxa. Each bacterium has a single member of the VUT/ECF, UBS1 and NAAT families, but only Sco has a member of the VIT and UIT1 families while only Mxa has a PSE family member. While these systems are all expected to catalyze uptake, their substrates are diverse and in several cases, uncertain (see TCDB). The TSUP family is well represented with 3 members in Sco and 6 in Mxa. Several of these systems probably take up sulfur-containing compounds [89]. Finally, the last of the secondary carrier families represented, the Bacterial Murein Precursor Exporter (MPE) Family [83], involved in cell wall biosynthesis, is present in both bacteria as expected. Mxa, however, has only one such member, while Sco has 4. It can be proposed that these distinct paralogues function at different stages of development in different cell types.

### Primary active transporters

#### ***ABC porters***

ABC transporters consist of one or two multispanning integral membrane protein(s) (homo- or heterodimers), one or two cytoplasmic ATPase(s) (also homo- or heterodimers), and for most uptake (but not efflux) systems, one or more extracytoplasmic receptor(s), each with a single N-terminal signal sequence. An examination of the integral membrane constituents of ABC transporters revealed that Sco has nearly three times as many ABC membrane proteins as does Mxa (202 versus 72). This difference, as well as the nearly four-fold greater number of MFS carriers in Sco, provides the majority of differences in the numbers of membrane transport proteins found within these two organisms.

Table 9 lists the families, numbers per family, and probable substrates of the ABC uptake proteins found in these two organisms. ABC porters include 3 independently evolving protein types, ABC1, ABC2 and ABC3, and all three types are represented in both Sco and Mxa [28]. The most striking difference between Sco and Mxa is the large number of sugar porters in Sco (85) as compared with Mxa (6). However, Sco has 12 amino acid and 17 peptide ABC transport proteins while Mxa has only 4 and 3, respectively. It seems that while Mxa primarily uses secondary carriers of the OPT family for peptide uptake, Sco primarily uses transporters of the ABC superfamily.

**Table 9:** ABC uptake porters in Sco and Mxa

**ABC Family**			**Sco**	**Mxa**
1	Carbohydrate Uptake Transporter-1 (CUT1) Family	Carbohydrates	75	4
2	Carbohydrate Uptake Transporter-2 (CUT2) Family	Carbohydrates	10	2
3	Polar Amino Acid Uptake Transporter (PAAT) Family	Polar amino acids	5	1
4	Hydrophobic Amino Acid Uptake Transporter (HAAT) Family	Non-polar amino acids	6	2
5	Peptide/Opine/Nickel Uptake Transporter (PepT) Family	Peptides, oligosaccharides	17	3
6	Sulfate/Tungstate Uptake Transporter (SulT) Family	Sulfate	1	1
7	Phosphate Uptake Transporter (PhoT) Family	Phosphate	3	2
8	Molybdate Uptake Transporter (MolT) Family	Molybdate	1	1
10	Ferric Iron Uptake Transporter (FeT) Family	Iron		2
11	Polyamine/Opine/Phosphonate Uptake Transporter (POPT) Family	Polyamines/opines/phosphonates	3	
12	Quaternary Amine Uptake Transporter (QAT) Family	Quaternary/amines	6	2
14	Iron Chelate Uptake Transporter (FeCT) Family	Iron chelates	8	4
15	Manganese/Zinc/Iron Chelate Uptake Transporter (MZT) Family	Mn^2+^/Zn^2+^/Fe^2+^ chelates	2	1
17	Taurine Uptake Transporter (TauT) Family	Taurine	2	2
18	Cobalt Uptake Transporter (CoT) Family	Cobalt (Co^2+^)	2	
20	*Brachyspira* Iron Transporter (BIT) Family	Iron	1	
21	Siderophore-Fe^3+^ Uptake Transporter (SIUT) Family	Siderophore-iron	2	2
23	Nickel/Cobalt Uptake Transporter (NiCoT) Family	Nickel; cobalt	2	
24	Methionine Uptake Transporter (MUT) Family	Methionine	1	1
27	γ-Hexachlorocyclohexane (HCH) Family	γ-hexachlorohexane/cholesterol	2	4
32	Cobalamin Precursor (B_12_-P) Family	Vitamin B12 precursors	2	

Numbers of integral membrane ABC uptake proteins in Sco and Mxa arranged by family.

These two organisms have similar low numbers of systems for inorganic anions, sulfate, phosphate, molybdate and phosphonates, but neither has an ABC nitrate/nitrite uptake system. Instead, they both have MFS-type nitrate/nitrite transporters (see above). Sco has about 4 times as many ABC amine transport proteins as does Mxa. These two organisms have similar numbers of ABC iron uptake proteins (11 and 8, respectively). ABC uptake systems for inorganic cations are rare in both bacteria. Vitamin transporters are also scarce.

ABC-type export systems are less numerous than uptake systems in both organisms. However, some families are well represented in one or the other organism. Both have at least one putative LPS precursor export system (Family 103), several lipid exporters (Family 106), and several lipoprotein exporters (Family 125) (Table 10). ABC-type drug exporters are prevalent but with striking differences between the two organisms. Sco has ten DrugE1 export proteins (Family 105) while Mxa has only one. Both have a single DrugE2 exporter (Family 117), but while Sco has only one DrugE3 export protein (Family 119), Mxa has six. Most strikingly, while Sco has only one macrolid export protein (Family 122), Mxa has 16. They both have MDR pumps belonging to other ABC export families, including eukaryotic-type systems. In Mxa, two of these belong to the MDR Family (Family 201), while in Sco, 1 belongs to the EPP Family (Family 204). Protein and peptide exporters can also be found, but no family predominates in either organism, and representation of one family in one of these bacteria does not correlate with representation in the other (Table 10). It seems clear that these two organisms have solved the problems of macromolecular and drug export using very different transport systems and mechanisms. This fact probably reflects the independent evolution of the two sporulating organisms’ lifestyles, as well as the production and secretion of different types of molecules. Thus, in spite of their striking physiological similarities (see Discussion), Sco and Mxa have used very different types of transport systems to satisfy their metabolic and developmental needs.

**Table 10:** ABC export porters in Sco and Mxa

**TC #**	**Family name**	**Known substrate range**	**ABC Type**	**Sco**	**Mxa**
3.A.1.103	Lipopolysaccharide Exporter (LPSE)	LPS	2	2	1
3.A.1.105	Drug Exporter-1 (DrugE1)	Drugs	2	10	1
3.A.1.106	Lipid Exporter (LipidE)	PL, LPS, Lipid A, Drugs, Peptides	1	6	3
3.A.1.107	Putative Heme Exporter (HemeE)	Heme, Cytochrome c	2		1
3.A.1.109	Protein-1 Exporter (Prot1E)	Proteins	1		1
3.A.1.110	Protein-2 Exporter (Prot2E)	Proteins	1		1
3.A.1.111	Peptide-1 Exporter (Pep1E)	Bacteriocin, Peptides	1	2	1
3.A.1.112	Peptide-2 Exporter (Pep2E)	Other Peptides	1	1	
3.A.1.115	Na^+^ Exporter (NatE)	Sodium	2		1
3.A.1.117	Drug Exporter-2 (DrugE2)	Drugs, Lipids, Dyes	1	1	
3.A.1.119	Drug/Siderophore Exporter-3 (DrugE3)	Drugs, Siderophores	1	6	
3.A.1.122	Macrolide Exporter (MacB)	Macrolides, Heme	3	1	16
3.A.1.123	Peptide-4 Exporter (Pep4E)	Drugs, Peptides	1	1	
3.A.1.125	Lipoprotein Translocase (LPT)	O.M. Lipoproteins	3	7	3
3.A.1.127	AmfS Peptide Exporter (AmfS-E)	Peptides, Morphogens	2	2	
3.A.1.129	CydDC Cysteine Exporter (CydDC-E)	Cysteine	1	1	
3.A.1.132	Gliding Motility ABC Transporter (Gld)	Polysaccharides, Copper Ions	2		2
3.A.1.134	Peptide-7 Exporter (Pep7E)	Peptides, Bacteriocins	3	1	
3.A.1.135	Drug Exporter-4 (DrugE4)	Drugs	1	2	
3.A.1.140	FtsX/FtsE Septation (FtsX/FtsE)	Septation		1	1
3.A.1.141	Ethyl Viologen Exporter (EVE)	Ethylviologen		2	2
3.A.1.201	Multidrug Resistance Exporter (MDR)	Drugs, Fatty Acids, Lipids	1		2
3.A.1.204	Eye Pigment Precursor Transporter (EPP)	Pigments, Drugs, Hemes	2	1	
3.A.1.210	Heavy Metal Transporter (HMT)	Drugs, Metal Conjugates, Heme	1	1	1

Numbers of integral membrane ABC export proteins in Sco and Mxa arranged by family.

#### ***ATPases in Sco and Mxa***

Both Sco and Mxa have a single F-type ATPase as indicated by the 3 integral membrane constituents listed in Additional file 1: Table S1 and Additional file 2: Table S2. These enzymes function to interconvert chemiosmotic energy (the proton motive force, pmf) with chemical energy (ATP). They both also have an H^+^-translocating pyrophosphatase complex. P-type ATPases in general appear to function in mediating stress responses in prokaryotes, and their occurrence by family in numerous organismal types has been defined [90,91]. Sco has eight such enzymes while Mxa has seven. While only Mxa has a Ca^2+^-ATPase (Family 2) and only Sco has a heavy metal ATPase (Family 6), both have the three components of Kdp-type K^+^ uptake ATPases as well as three distinct copper ATPases. Remaining P-type ATPases in these organisms are functionally uncharacterized. Sco has two members of Family 23 and one member of Family 25 while Mxa has one member each of Families 27 and 32. While Family 23 members are of the type 2 ATPases with 10 TMSs, Families 25, 27 and 32 have the basic type 1 topology of 6 TMSs plus or minus one or two extra N-terminal TMSs [91]. One member of Family 27 has been shown to function in the insertion of copper into copper-dependent oxidases, such as cytochrome oxidase, but not in copper tolerance [92]. This is probably the function of the enzyme in Mxa. Since both organisms have complete cytochrome oxidase systems, it may be that Sco uses an alternative mechanism to insert copper during the biogenesis of this enzyme complex. Possibly, it uses one of its three copper ATPases.

#### ***Protein secretion***

As expected, both organisms have the general secretory pathway for protein export (TC# 3.A.5) as well as the Twin arginine targeting (Tat) protein secretion system (TC# 2.A.64) and the DNA translocase (DNA-T). Sco, but not Mxa, appears to have a type IV protein/DNA secretion system (found in both Gram-negative and Gram-positive bacteria). However, only Mxa has components of type II (MTB) and type III protein secretion systems, both present in certain Gram-negative bacteria but lacking in Gram-positive bacteria [93,94].

### Group translocation via the phosphoenolpyruvate-dependent sugar-transporting phosphotransferase system (PTS)

Both Sco and Mxa have proteins of the PTS. However, while Mxa has only one sugar transporting system of the mannose family, Sco has five systems, one probably specific for glucose and maltose, two specific for N-acetyl glucosamine and related sugars, a fourth specific for fructose, and a fifth that may transport L-ascorbate [95-98]. A link between N-acetyl glucosamine metabolism and the control of development in Sco has been reported [99,100], possibly explaining why two such systems are present. Thus, in agreement with observations previously discussed in this article, Sco apparently relies more heavily on sugars for carbon and energy than does Mxa, and the published data implies that it uses availability of these sugars (or at least N-acetyl glucosamine) to control development.

### Oxidative metabolism

Both organisms have homologues of the putative fatty acid transporters of the FAT Family, DsbD homologues for the transfer of electrons across the cytoplasmic membrane for periplasmic sulfhydryl oxidoreduction, members of the Prokaryotic Molybdopterin-containing Oxidoreductase (PMO) Family, and a succinate dehydrogenase. The striking similarities between the proton-pumping electron transfer complexes of the TC 3.D subclass are particularly noteworthy. Apparently, Sco and Mxa have quantitatively similar complements of electron transfer carriers of all types, the most striking parallels we have observed for these two organisms.

### Transporters of unknown mechanisms of action

It is interesting that both Sco and Mxa have members of the TerC and HCC families although in different numbers. While Mxa has two of each, Sco has 5 TerC homologues and 9 HCC proteins. Although one TerC protein has been implicated in tellurium resistance, functions of its many homologues are probably diverse. HCC homologues, some or all of which are likely to be Mg^2+^ transporters, consist of three domains, an N-terminal 4 TMS DUF21 domain, a central nucleotide-binding CBS domain, and a C-terminal HlyC/CorC domain. Only proteins within this family that possess the DUF21 domain are likely to be divalent cation transporters. All of the homologues in Sco and Mxa have the DUF21 domain, suggesting that they serve this function. Why Sco would need nine such proteins is a mystery, as most bacteria have only one or two, or lack them altogether. It can be proposed that they function in the regulation of differentiation where Mg^2+^ may play crucial roles in regulating the many ATP-dependent kinases, including, but not limited to, the 44 ser/thr kinases (see Discussion).

### Observed differences in gene size and number

We downloaded Sco A3(2) and Mxa DK 1622 from Ensembl Bacteria (http://bacteria.ensembl.org/index.html). In Sco, there were 8,154 sequences and in Mxa 7,331. The average protein size was 326 in Sco and 379 in Mxa. The genome size of Sco is 8.7 million bps and of Mxa, 9.1 million bps. We used Glimmer 3 (microbial Genscan) [101] against Sco (NC_003888) and Mxa (CP000113) and found 8,213 gene predictions for Sco. The average length (nt) was 939. For Mxa, there were 7,656 gene predictions, with an average length (nt) of 1075. These data are consistent with the concept that Sco has more and smaller genes, than Mxa.

### Transporters of experimentally verified function in Sco and Mxa

We have screened the published literature for articles that provide experimental information about transporters in Sco and Mxa. A summary of the findings are presented in Table 11 which gives the protein designations, the Sco or Mxan genome numbers and the references in column 1, the UniProt accession numbers in column 2, the TC#s of the transport systems in column 3, and the probable functions plus additional information if available in column 4. Of these proteins, only one system (AreABCD) of Sco was not included in our initial G-blast screen. It was missed because these sequences were too distant to anything then in TCDB to give a score better than our cutoff value of 0.001. The AreABCD export system has been assigned TC# 3.A.1.146.1 and represents a new family within the ABC superfamily.

**Table 11:** Functionally characterized Sco and Mxa proteins

**Protein designation; Sco# or Mxan#, and reference**^**1**^	**UniProt Acc#**	**TC#**	**Probable or established function**
***S. coelicolor***			
MscL; Sco3190 [102]	Q9KYV5	1.A.22.1.10	MscL, osmotic adaptation channel that influences sporulation and secondary metabolite production.
GlcP1/2; Sco7153; Sco5578 [103]	Q7BEC4	2.A.1.1.35	MFS major glucose uptake porters (two identical sequences at the AA level, and having a single substitution on the NT level).
MdrA; Sco4007 [104]	Q9ADP8	2.A.1.36.4	Putative MDR transporter; may export hydrophobic cationic compounds.
PitH1 and 2; Sco4138 and Sco1845 [105]	Q9KZW3, Q9RJ23	2.A.20.1.5 and 6	Two putative low-affinity inorganic phosphate (P_i_) uptake porters.
DasABC: Sco5232-4 (R, M, M). MsiK: Sco4240 (C) [106]	Q9K489-91,Q9L0Q1	3.A.1.1.33	DasABC/MsiK; system for the uptake of chitin-degradation products.
Agl3EFG porter (R, M, M; Sco7167-Sco7165 [107]; Agl3K (C; unknown)	Q9FBS7-5	3.A.1.1.43	Sugar uptake porter; induced by trehalose and melibiose using a GntR transcription factor. May use the MsiK ATPase [106].
MalEFG; Sco2231-Sco2229 (R, M, M) [108]; MalK (C) unknown.	Q7AKP1, Q9KZ07-8	3.A.1.1.44	Sugar uptake porter; involved in maltose and maltodextrin uptake. May use the MsiK ATPase [106].
XylFGH.	O50503-5	3.A.1.2.24	Xylose uptake porter; transcriptionally regulated by a GntR-type protein, ROK7B7.
XylF, Sco6009 (R; 1 N-terminal TMS);			
XylG, Sco6010 (C; ATP-binding, no TMSs);			
XylH, Sco6011 (M; 12 TMSs); [109]			
Probable ABC peptide uptake porter; Sco5476-80 (M, R, M, C, C) [110]	O86571-5	3.A.1.5.34	Probably takes up a peptide involved in the regulation of sporulation and secondary metabolite production.
Sco5117-Sco5121 (R, M, M, C, C) [111]	Q9F353-49	3.A.1.5.35	Probable oligopeptide uptake porter.
BldKA-D and Sco5116; Sco5112-6 (M, R, M, C, C) [112]	Q93IU3-0; Q8CJS2	3.A.1.5.36	BldKA-D and Sco5116; peptide uptake porter induced by S-adenosylmethionine.
DesABC; Sco7499-8, Sco7400 (R, M-M, C) [113]	Q9L177-9	3.A.1.14.12	Desferrioxamine B uptake porter.
CchCDEF; Sco0497-4 (M, M, C, R) [113]	Q9RK09-12	3.A.1.14.13	Ferric iron-coelichelin uptake porter.
DesEFGH; Sco2780 (R), Sco1785-7 (C, M, M) [113]	Q9L07; Q9S215-3	3.A.1.14.22	Putative ferric iron-desferrioxamine E uptake porter.
SclAB; Sco4359-60 (C, M) [114]	Q9F2Y8-7	3.A.1.105.13	SclAB transporter; confers acyl depsipeptide (ADEP) resistance. ADEP has antibiotic activity.
RagAB; Sco4075-4 (C, M) [115]	Q7AKK4-5	3.A.1.105.14	RagAB exporter; involved in both aerial hyphae formation and sporulation.
SoxR regulon ABC exporter; Sco7008 (M, C) [116]	Q9KZE5	3.A.1.106.9	Putative SoxR-regulated drug exporter; SoxR responds to extracellular redox-active compounds such as actinorhodin.
AreABCD; Sco3956-9 (C, M, C^’^, M^’^) [117]	Q9ZBX6-3	3.A.1.146.1	Putative drug exporter; possibly specific for actinorhodin (ACT) and undecylprodigiosin (RED).
H^+^-PPase; Sco3547 [118]	Q6BCL0	3.A.10.2.2	H^+^-translocating inorganic pyrophosphatase.
***M. xanthus***			
MmrA; MXAN_5906 [119]	Q1CZY0	2.A.1.2.83	Homologous to drug exporter; possibly involved in amino acid uptake and antimicrobial export.
TatABC; MXAN_2960, MXAN_5905-4, [120]	Q1D854, Q1CZY1-2	2.A.64.1.2	Twin arginine targeting protein translocase.
RfbAB; MXAN_4623-2 (M, C) [121]	Q1D3I2-3	3.A.1.103.4	Putative lipopolysaccharide exporter.
AbcA; MXAN_1286 (M-C) [122]	Q1DCT0	3.A.1.106.10	AbcA; involved in molecular export; required for the autochemotactic process.
PilGHI; MXAN_5782-0 (R, C, M) [123]	O30384-6	3.A.1.144.5	Necessary for social motility, pilus assembly and pilus subunit (PilA) export.

^1^ M: Membrane component; C: cytoplasmic ATPase energizer; R: Extracytoplasmic solute receptor of an ABC transporter.

The systems listed in Table 11 will not be discussed individually as the information provided in the table is self-explanatory. However, some entries are worthy of elaboration. For example, MdrA (Sco4007, [104]), is a putative MFS multi-drug exporter, based on the specificity of the regulatory protein that controls expression of its structural gene.

Three systems (DasABC, AglEFG and MalEFG; TC#s 3.A.1.1.33, 3.A.1.1.43 and 3.A.1.1.44) were each encoded within operons that encoded a receptor (R) and two membrane (M) proteins but no cytoplasmic ATPase (C). In the case of the DasABC system, the separately encoded MsiK (multiple sugar import-K) ATPase protein has been shown to serve as the energy-coupling constituent of the system [106]. We infer that the same is true for the AglEFG and MalEFG systems because: (1) each of these sets of proteins are encoded in an operon that lacks a cytoplasmic ATPase, and (2) all three systems belong to the same TC family (CUT1; TC#3.A.1.1) in which interchangeability of ATPases has been documented [106], and (3) an *msiK* null mutant has been shown to be unable to utilize several disaccharides including maltose [106].

Two ABC ferric iron-hydroxamate uptake porters of Sco have been characterized [113]. The CchCDEF system has been assigned TC# 3.A.1.14.13 while the DesABC system has been assigned TC# 3.A.1.14.12. Additionally, a putative ABC receptor, DesE, has been characterized, but its cognate transport proteins have not been identified [113]. Because the complete transport system was not recognized, this receptor was not entered into TCDB, and because it gave a poor score with its closest homologue, it was not recognized by G-BLAST. We have previously shown that the three constituents (receptor protein, R; membrane protein, M; and cytoplasmic ATPase, C) of ABC uptake porters coevolved almost without exception, therefore forming analogous phylogenetic trees [124]. However, while the genes encoding a complete ABC porter often cluster together, the receptor and/or ATPase may cluster separately. Based on these facts, we attempted to identify the most probable set of ABC proteins that function with DesE.

In order to predict which membrane (M) and cytoplasmic (C) ATPase proteins function with DesE, DesE was blasted against TCDB and brought up FhuD (3.A.1.14.7) as the best hit, the receptor for the ferric iron-hydroxamate porters of *Staphylococcus aureus*, FhuBCD,D2. FhuB, the membrane constituent, was then blasted against the Sco database and brought up Sco1785 and Sco0497 (CchC) as top hits. FhuC, the ATPase of the *S. aureus* porter, brought up Sco1787 and Sco0495 (CchE) as the top hits. Examination of the gene cluster containing Sco1785 and Sco1787 revealed that Sco1786 is a second membrane protein encoded in the same operon. However, no receptor was encoded in this operon or the surrounding gene cluster. We therefore propose that the characterized receptor, DesE, functions with Sco1785/Sco1786/Sco1787. We have designated this system DesEFGH, and it has been assigned TC# 3.A.1.14.22 (see Table 11).

## Discussion

*Streptomyces coelicolor* (Sco) and *Myxococcus xanthus* (Mxa) have genomes of about the same size, each present on a single chromosome. They have expanded genomes relative to almost all other prokaryotes with fully sequenced genomes. However, the numbers of integral membrane transport proteins encoded in these two genomes differ dramatically. We identified 658 in Sco, but only 355 in Mxa, a 93% difference. Part of this difference reflects the total number of proteins encoded; Mxa has been reported to have 10% fewer protein-encoding genes than Sco. However, the primary explanation for the difference in numbers of transport proteins appears to come from studies aimed at determining the nature of the “expanded” gene sets. As reported by Goldman et al. [12], for Mxa, the increased genome size evidently resulted from extensive gene duplication and divergence relative to other bacteria of normal genome size, but of only certain functional types. More than 1500 duplications specific to the myxobacterial lineage were identified relative to other δ-proteobacteria, and these represented 15.6% of the total genes. The amplified genes they identified dealt primarily with cell-cell signaling, small molecule sensing, and integrative transcriptional regulation [11]. For example, 97 serine/threonine protein kinases were identified in Mxa (44 were found in Sco), although other δ-proteobacteria with “normal” sized genomes exhibit 0–3 such enzymes. Corresponding increases in some proteins (e.g., chaperones), but not other types of genes (e.g., transport systems), were generally observed in Mxa [12,36] and this study]. By contrast, in Sco, certain types of transporters were extensively amplified as shown here.

As for Mxa, there has been very considerable expansion of regulatory genes in Sco relative to other actinobacteria such as *Mycobacterium tuberculosis* and *Corynebacterium diptheriae*[11,16]. The total number of regulatory genes identified in Sco was 965 or 12.3%, about the same as reported for Mxa [11,12]. However, in Sco, the numbers of transport and secreted proteins expanded relative to *M. tuberculosis* and *C. diptheriae*, although such extensive expansion was not observed for Mxa. These observations help to explain the differences in transport protein numbers in these two bacteria.

Mxa has a large repertoire of polyketide synthases, about twice that in Sco [12]. Since these enzymes are often in excess of 2,000 amino acyl residues in size, this fact may help to explain why the Mxa genome encodes fewer polypeptide chains than the Sco genome. In fact, the average protein size in Mxa is reported to be 376 aas/polypeptide chain with approximately 90% of the genome coding for proteins [12]. In Sco, it is 330 aas/polypeptide chain with approximately 89% of the genome coding for proteins [11]. Thus, the increased number of proteins in Sco is compensated for by their decreased average size. It would be interesting to do a comparative study of protein sizes for the different functional types of proteins in a range of organisms to determine if this difference is specific or general.

Species of *Streptomyces* and Myxobacteria belong to two different bacterial phyla—the actinobacteria (high G + C Gram-positive bacteria) and proteobacteria (Gram-negative δ-proteobacteria)—and are therefore only very distantly related. However, (a) both are saprophytic microorganisms, (b) both encode multiple complex programs of differentiation, (c) both produce spores within multicellular structures (aerial mycelia and fruiting bodies, respectively), (d) both produce wide ranges of secondary metabolites including many pigments and macrolid antibiotics, (e) both communicate using numerous secreted small molecules, and (f) both degrade a wide range of extracellular macromolecules [2,5,14,86,125-129]. These two organisms have the most complex lifestyles of any bacteria currently under careful experimental scrutiny, and both have genomes that are larger than almost any other prokaryote whose genomes have been sequenced, thus accounting for their expanded genetic repertoire.

In view of these similarities, we compared the range of transport mechanisms and substrates used by these two developmental organisms. Such knowledge, we reasoned, would allow us to determine if they introduce developmental complexity along similar lines at the molecular level. Our studies led to the general conclusion that these two organisms have solved their metabolic needs and created programs of differentiation by entirely different means. For example, while Sco has a plethora of sugar, organic anion, and amino acid uptake systems of very specific types, Mxa has relatively few. In retrospect, this may be explained since myxobacteria are “micropredators,” lysing other microorganisms which they use as food sources, while *Streptomyces* species may have evolved as beneficial, growth-promoting symbionts of other organisms [126,128,129]. It seems likely that the programs of development exhibited by these two organisms evolved independently, and the similarities reflect the limited numbers of options available. Other physiological similarities noted above possibly reflect a convergent evolutionary process, resulting from similarities in the habitats in which these organisms live.

Several surprises resulted from the analyses reported here. For example, Mxa has a member of the AAA family of nucleotide (ATP, ADP, NAD^+^, etc.) transporters, normally found only in obligatory intracellular parasites. It also has more (9) CorC-type putative Mg^2+^ transporters than we have encountered in any other organism. Mxa additionally has a Ca^2+^-ATPase, although such an enzyme was lacking in Sco where a Ca:H^+^ antiporter, lacking in Mxa, could be identified. It is known that both organisms rely on Ca^2+^ for developmental regulation [72-75]. We also discovered homologues of Spinster proteins, believed to be sphingosine-1-phosphate transporters in animals [53-55]. BLAST searches revealed that many bacteria have these proteins. Their substrates and functions may prove to be similar to those in animals since myxobacteria have been shown to have outer membrane sphingolipids [57].

Gram-negative bacteria have a number of transport systems that allow biogenesis, maintenance and function of the outer membranes of these organisms. These include the TolQ/R energizers of outer membrane receptor-mediated uptake of large molecules such as iron-siderophores and large vitamins, and they are known to function as energizers of gliding motility in Mxa [130]. They also include an outer membrane protein insertion porin apparatus (Bam or OmpIP systems; TC#1.B.33) and the outer membrane lipopolysaccharide export porin complex 3 (LPS-EP systems; TC#1.B.42). All of these systems were found in Mxa but could not be detected in Sco. Although Sco has an outer membrane of a very different composition [131], this observation implies that entirely different types of systems, serving the same functions, must exist in actinobacteria. It therefore seems clear that the comparative analyses reported here will open up new fields of microbial inquiry.

## Conclusions

Analyses of transport proteins in two of the largest genome bacteria, both capable of sporulation and antibiotic production, one an actinobacterium and one a myxobacterium, revealed that these two organisms have evolved complexity via entirely different pathways. While both have amplified certain sets of transport protein-encoding genes, they differ in the degrees of amplification and the nature of the transporters amplified. The results provide insight into the evolution of prokaryotic complexity.

## Methods

The proteomes of *S. coelicolor* strain A3(2) (Sco) and *M. xanthus* strain DK1622 (Mxa) were screened for homologues of all proteins contained in the Transporter Classification Database (TCDB; http://www.tcdb.org) as of September, 2011 using G-BLAST [132]. FASTA-formatted protein sequences of the completed genomes of Sco and Mxa were used. Each putative open-reading frame (ORF) was used as a query in the BLASTP software to search for homologous proteins in TCDB. The SEG low complexity filter was not used. In addition, each ORF was scanned with the HMMTOP 2.0 program [133] to predict the number of putative transmembrane segments (TMSs). The WHAT program [134] was used to resolve the differences in the numbers of TMSs between Sco proteins, Mxa proteins, and their TCDB homologues. A cut-off value of 0.001 was used with the G-BLAST program so proteins retrieved with larger values (greater sequence divergence) were not recorded. After analysis of these proteins was conducted, proteins with e-values between 0.1 and 0.001 were retrieved, and the more distant homologues to TC entries were identified. Proteins with 0 predicted TMSs were eliminated so that only integral membrane proteins (primarily multi-spanning membrane proteins) were retrieved. Some single TMS proteins, including many extracytoplasmic solute binding receptors of ABC transport systems, were often predicted to lack a TMS and therefore were not included in our study.

Candidate proteins were subsequently examined in greater detail to estimate their substrate specificities. On the basis of the numbers and locations of TMSs, as well as degrees of sequence similarities with entries of known function in TCDB, transport proteins were classified into families and subfamilies of homologous transporters according to the classification system presented in TCDB [17,18]. Regions of sequence similarity were examined to ensure that homology was in transmembrane regions and not in hydrophilic domains. Proteins encoded within single operons were often identified in order to gain evidence for multicomponent systems and to help deduce probable functions. Operon analyses were performed for candidate proteins with assigned or unassigned transport functions.

The substrate specificities of particular homologues identified in the sequenced genomes were sometimes predicted based on homology to functionally characterized genes and from their genomic context. Assignment to a family or subfamily within the TC system often allows prediction of substrate type with confidence [13,20,135-137]. When an expected transport protein constituent of a multi-component transport system could not be identified with BLASTP, tBLASTn was performed because such expected proteins are sometimes undetectable by BLASTP due to sequencing errors, sequence divergence, or pseudogene formation.

Transport proteins thus obtained were systematically analyzed for unusual properties using published [132] and unpublished in-house software. Unusual properties can result from events such as genetic deletion and fusion, sometimes resulting in the gain or loss of extra domains or the generation of multifunctional proteins. Such results can be reflective of the actual protein sequence, but can also be artifactual, due to sequencing errors or incorrect initiation codon assignment. In the latter cases, but not the former, the protein sequences were either corrected when possible or eliminated from our study.

This theoretical bioinformatics study does not contain any experimental research that requires the approval of an ethics committee.

## Competing interests

The authors are not aware of any affiliations, memberships, funding, or financial holdings that might be perceived as affecting the objectivity of this review.

## Authors’ contributions

Conceived and designed the experiments MHS; Performed the experiments IG, GHN, DCY, PCGP; Analyzed the data: IG, GHN, DCY, AV; Contributed reagents/materials/analysis tools VSR; Wrote the paper IG, GHN, DCY, MHS. All authors read and approved the final manuscript.

## Supplementary Material

Additional file 1: Table S1 Sco transport proteins. Detailed description of Sco transport proteins and their homologues in TCDB, including comparison scores obtained via G-Blast and GSAT, substrate, substrate class, organism, phylum, and organismal domain. Proteins are organized from lowest to highest TC#. Click here for file

Additional file 2: Table S2 Mxa transport proteins. Detailed description of Mxa transport proteins and their homologues in TCDB, including comparison scores obtained via G-Blast and GSAT, substrate, substrate class, organism, phylum, and organismal domain. Proteins are organized from lowest to highest TC#. Click here for file

Additional file 3: Table S3 Chromosomal distribution of Sco transporters. Sco transport proteins distributed by chromosomal arms and core. Click here for file
