# Supplementary material for: Comparative genomics of transport proteins in developmental bacteria: Myxococcus xanthus and Streptomyces coelicolor
Source: BMC Microbiol. 2013 Dec 5;13:279. doi: 10.1186/1471-2180-13-279 (PMC3924187; doi:10.1186/1471-2180-13-279)
Supplement: Additional file 1: Table S1 — Sco transport proteins. Detailed description of Sco transport proteins and their homologues in TCDB, including comparison scores obtained via G-Blast and GSAT, substrate, substrate class, organism, phylum, and organismal domain. Proteins are organized from lowest to highest TC#. [file 1471-2180-13-279-S1.docx]

**Table S1.** **Sco Transport proteins.** Detailed description of Sco transport proteins and their homologues in TCDB, including comparison scores obtained via G-Blast and NCBI, substrate, substrate class, organism, phylum, and domain. Proteins are organized from lowest to highest TC#.

| **Hit TCID** | **Family Abrv.** | **Query Acc #** | SCO# | **Hit Acc #** | **e-Value** | **Query TMS#** | **Hit TMS#** | **TM-Overlap Score** | **Substrate** | **Substrate Class** | **Organism** | **Phylum** | **Domain** |
| --- | --- | --- | --- | --- | --- | --- | --- | --- | --- | --- | --- | --- | --- |
| 1.A.1.1.1 | VIC | P0A333 | SCO7660 | P0A334 | 3.09E-087 | 2 | 2 | 2.3 | K^+^ | Cations | *Streptomyces lividans* | Actino-bacteria | Bacteria |
| 1.A.1.7.2 | VIC | O54192 | SCO5963 | Q6X308 | 3.99E-008 | 4 | 4 | 1.65 | K^+^ | Cations | *Arabidopsis thaliana* | Angiosperms | Eukarya |
| 1.A.1.13.2 | VIC | Q9XA52 | SCO3826 | O27564 | 1.10E-015 | 2 | 2 | 1.15 | K^+^ | Cations | *Methanobacterium thermoautotrophicum* | Euryarchaeota | Archaea |
| 1.A.1.13.3 | VIC | O86498 | SCO6330 | Q979Z2 | 1.72E-007 | 4 | 3 | 1.6 | K^+^ | Cations | *Thermoplasma volcanium* | Euryarchaeota | Archaea |
| 1.A.1.17.2 | VIC | Q9FBY6 | SCO7196 | Q8Y5K1 | 5.47E-025 | 4 | 6 | 3.05 | K^+^ | Cations | *Listeria monocytogenes* | Firmicutes | Bacteria |
| 1.A.1.23.2 | VIC | Q9FBZ5 | SCO7187 | Q5H8A6 | 3.29E-051 | 2 | 4 | 1.75 | K^+^ | Cations | *Lotus japonicus* | Angiosperms | Eukarya |
| 1.A.8.2.1 | MIP | P19255 | SCO1659 | P18156 | 1.13E-059 | 6 | 7 | 4.3 | Glycerol | Sugars & polyols | *Bacillus subtilis* | Firmicutes | Bacteria |
| 1.A.8.9.1 | MIP | Q9AK60 | SCO4057 | P47862 | 3.76E-043 | 6 | 6 | 3.2 | Water and glycerol | Sugars & polyols | *Rattus norvegicus* | Chordata | Eukarya |
| 1.A.11.1.3 | ClC | Q9ZBP6 | SCO5583 | Q79VF1 | 1.74E-100 | 11 | 11 | 7.9 | NH_3_ | Amines, amides, polyamines, & organocations | *Corynebacterium glutamicum* | Actino-bacteria | Bacteria |
| 1.A.22.1.4 | MscL | Q9KYV5 | SCO3190 | Q2KCQ1 | 1.03E-017 | 2 | 2 | 2.25 | N/A | Nonspecific ion | *Rhizobium etli* | Proteobacteria | Bacteria |
| 1.A.23.1.2 | MscS | Q9S2Y1 | SCO2070 | Q9EV03 | 1.09E-007 | 5 | 12 | 2.75 | Osmolytes/nonspecific ion | Nonspecific ion | *Erwinia chrysanthemi* | Proteobacteria | Bacteria |
| 1.A.23.2.1 | MscS | Q9KZF2 | SCO7001 | P0C0S1 | 1.02E-026 | 5 | 4 | 2.55 | N/A | Nonspecific ion | *Escherichia coli* | Proteobacteria | Bacteria |
| 1.A.23.3.1 | MscS | Q9XA89 | SCO0836 | O34897 | 1.19E-031 | 3 | 2 | 0.95 | Osmolytes/nonspecific ion | Nonspecific ion | *Bacillus subtilis* | Firmicutes | Bacteria |
| 1.A.23.3.1 | MscS | Q9L1X9 | SCO2656 | O34897 | 1.19E-028 | 3 | 2 | 1.15 | Osmolytes/nonspecific ion | Nonspecific ion | *Bacillus subtilis* | Firmicutes | Bacteria |
| 1.A.34.1.1 | 1.A.34 | Q9X8M5 | SCO3368 | P71044 | 2.37E-011 | 1 | 1 | 0.55 | Nonspecific | Proteins | *Bacillus subtilis* | Firmicutes | Bacteria |
| 1.A.35.1.1 | MIT | Q9ADA0 | SCO1667 | P0ABI4 | 3.22E-015 | 2 | 2 | 1.1 | Mg^2+^ | Cations | *Escherichia coli* | Proteobacteria | Bacteria |
| 1.A.35.3.1 | MIT | Q9FBK1 | SCO5157 | Q58439 | 1.40E-015 | 2 | 3 | 0.3 | Divalent metal ions (Mg^2+^, Co^2+^, Ni ^2+^) | Cations | *Methanococcus jannaschii* | Euryarchaeota | Archaea |
| 1.A.62.3.1 | 1.A.62 | O86576 | SCO5481 | Q981D4 | 5.10E-026 | 7 | 7 | 5.75 | Ca^2+^ | Cations | *Sulfolobus solfataricus* | Crenarchaeota | Archaea |
| 1.A.62.3.1 | 1.A.62 | Q9RKM3 | SCO4104 | Q981D4 | 1.97E-024 | 7 | 7 | 5.85 | Ca^2+^ | Cations | *Sulfolobus solfataricus* | Crenarchaeota | Archaea |
| 1.C.42.1.1 | BAPA | Q9RIU6 | SCO0960 | P13423 | 3.14E-005 | 1 | 1 | 0.55 | Antigen/nonspecific | Proteins | *Bacillus anthracis* | Firmicutes | Bacteria |
| 2.A.1.1.15 | MFS | Q9KZR2 | SCO2889 | P95908 | 4.99E-023 | 12 | 12 | 9.3 | Glucose/nonspecific | Sugars & polyols | *Sulfolobus solfataricus* | Crenarchaeota | Archaea |
| 2.A.1.1.35 | MFS | Q9ZBQ1 | SCO7153 | Q7BEC4 | 0 | 12 | 12 | 12.4 | Glucose ( 2-deoxyglucose) | Sugars & polyols | *Streptomyces lividans* | Actino-bacteria | Bacteria |
| 2.A.1.2.7 | MFS | Q9RKP4 | SCO2284 | P28246 | 3.35E-051 | 12 | 12 | 11.2 | Bicyclomycin, sulfathiazole, tetracycline, fosfomycin, arcriflavin, L-cysteine, etc. | Multiple Drugs | *Escherichia coli* | Proteobacteria | Bacteria |
| 2.A.1.2.8 | MFS | Q9KYE9 | SCO4121 | P39843 | 2.17E-008 | 12 | 12 | 10.05 | Spermicide, fluoroquinolones, acriflavin, chloramphenicol, ethidum bromide, etc. | Multiple Drugs | *Bacillus subtilis* | Firmicutes | Bacteria |
| 2.A.1.2.14 | MFS | Q9RDR3 | SCO2254 | P23910 | 4.19E-064 | 12 | 12 | 10.5 | Arabinose | Sugars & polyols | *Escherichia coli* | Proteobacteria | Bacteria |
| 2.A.1.2.14 | MFS | Q9X8V1 | SCO3915 | P23910 | 8.92E-061 | 12 | 12 | 10.85 | Arabinose | Sugars & polyols | *Escherichia coli* | Proteobacteria | Bacteria |
| 2.A.1.2.14 | MFS | Q9L223 | SCO6809 | P23910 | 0.000572752 | 12 | 12 | 9.65 | Arabinose | Sugars & polyols | *Escherichia coli* | Proteobacteria | Bacteria |
| 2.A.1.2.21 | MFS | O86692 | SCO6646 | P69367 | 1.52E-040 | 12 | 12 | 8.75 | Norfloxacin/enoxacin | Multiple Drugs | *Escherichia coli* | Proteobacteria | Bacteria |
| 2.A.1.2.21 | MFS | Q9EX44 | SCO0994 | P69367 | 3.07E-012 | 12 | 12 | 8.85 | Norfloxacin/enoxacin | Multiple Drugs | *Escherichia coli* | Proteobacteria | Bacteria |
| 2.A.1.2.21 | MFS | Q9FCG1 | SCO4208 | P69367 | 7.83E-010 | 12 | 12 | 9.35 | Norfloxacin/enoxacin | Multiple Drugs | *Escherichia coli* | Proteobacteria | Bacteria |
| 2.A.1.2.26 | MFS | Q9RI96 | SCO0079 | P0ADL1 | 3.77E-042 | 12 | 12 | 12 | Purine (ribonucleoside) | Nucleic acids | *Escherichia coli* | Proteobacteria | Bacteria |
| 2.A.1.2.32 | MFS | Q9F3M6 | SCO7662 | P31141 | 0 | 12 | 12 | 13.2 | Chloramphenicol | Specific Drugs | *Streptomyces lividans* | Actino-bacteria | Bacteria |
| 2.A.1.2.32 | MFS | Q9KZ03 | SCO7526 | P31141 | 1.42E-054 | 12 | 12 | 10.35 | Chloramphenicol | Specific Drugs | *Streptomyces lividans* | Actino-bacteria | Bacteria |
| 2.A.1.2.38 | MFS | Q93J85 | SCO2812 | Q56RY7 | 2.95E-007 | 12 | 12 | 7.65 | Tetracycline | Specific Drugs | *Acinetobacter sp.* | Proteobacteria | Bacteria |
| 2.A.1.3.3 | MFS | Q9RD97 | SCO2854 | Q50392 | 1.69E-032 | 14 | 16 | 11.55 | Multiple drugs (Acriflavin, ethidium bromide, fluoroquinolones, etc.) | Multiple Drugs | *Mycobacterium smegmatis* | Actino-bacteria | Bacteria |
| 2.A.1.3.5 | MFS | Q9F0Y3 | SCO4024 | Q54806 | 0 | 12 | 14 | 10.7 | Multiple drugs (Pristinamycin I & II, rifamycin, etc.) | Multiple Drugs | *Streptomyces pristinaespiralis* | Actino-bacteria | Bacteria |
| 2.A.1.3.5 | MFS | Q9L210 | SCO6822 | Q54806 | 1.12E-083 | 14 | 14 | 12.1 | Multiple drugs (Pristinamycin I & II, rifamycin, etc.) | Multiple Drugs | *Streptomyces pristinaespiralis* | Actino-bacteria | Bacteria |
| 2.A.1.3.5 | MFS | O54186 | SCO5957 | Q54806 | 1.21E-077 | 12 | 14 | 11.3 | Multiple drugs (Pristinamycin I & II, rifamycin, etc.) | Multiple Drugs | *Streptomyces pristinaespiralis* | Actino-bacteria | Bacteria |
| 2.A.1.3.5 | MFS | Q99QG4 | SCO5957 | Q54806 | 5.47E-074 | 13 | 14 | 10.75 | Multiple drugs (Pristinamycin I & II, rifamycin, etc.) | Multiple Drugs | *Streptomyces pristinaespiralis* | Actino-bacteria | Bacteria |
| 2.A.1.3.5 | MFS | Q8CJZ3 | SCO2498 | Q54806 | 5.18E-069 | 14 | 14 | 12.05 | Multiple drugs (Pristinamycin I & II, rifamycin, etc.) | Multiple Drugs | *Streptomyces pristinaespiralis* | Actino-bacteria | Bacteria |
| 2.A.1.3.5 | MFS | Q9RL22 | SCO0295 | Q54806 | 2.03E-068 | 14 | 14 | 12.25 | Multiple drugs (Pristinamycin I & II, rifamycin, etc.) | Multiple Drugs | *Streptomyces pristinaespiralis* | Actino-bacteria | Bacteria |
| 2.A.1.3.5 | MFS | Q9ZBW5 | SCO6553 | Q54806 | 1.44E-058 | 15 | 14 | 12.3 | Multiple drugs (Pristinamycin I & II, rifamycin, etc.) | Multiple Drugs | *Streptomyces pristinaespiralis* | Actino-bacteria | Bacteria |
| 2.A.1.3.5 | MFS | Q8CK59 | SCO0375 | Q54806 | 2.12E-052 | 14 | 14 | 9.2 | Multiple drugs (Pristinamycin I & II, rifamycin, etc.) | Multiple Drugs | *Streptomyces pristinaespiralis* | Actino-bacteria | Bacteria |
| 2.A.1.3.5 | MFS | Q9KXZ7 | SCO7346 | Q54806 | 2.08E-029 | 14 | 14 | 11.7 | Multiple drugs (Pristinamycin I & II, rifamycin, etc.) | Multiple Drugs | *Streptomyces pristinaespiralis* | Actino-bacteria | Bacteria |
| 2.A.1.3.5 | MFS | Q9FBJ9 | SCO5159 | Q54806 | 1.35E-014 | 12 | 14 | 9.1 | Multiple drugs (Pristinamycin I & II, rifamycin, etc.) | Multiple Drugs | *Streptomyces pristinaespiralis* | Actino-bacteria | Bacteria |
| 2.A.1.3.5 | MFS | Q9L134 | SCO7425 | Q54806 | 2.49E-011 | 12 | 14 | 10.3 | Multiple drugs (Pristinamycin I & II, rifamycin, etc.) | Multiple Drugs | *Streptomyces pristinaespiralis* | Actino-bacteria | Bacteria |
| 2.A.1.3.7 | MFS | Q53903 | SCO5076 | Q53903 | 0 | 12 | 12 | 12.6 | Actinorhodin | Specific Drugs | *Streptomyces coelicolor* | Actino-bacteria | Bacteria |
| 2.A.1.3.8 | MFS | Q9L004 | SCO2309 | Q04733 | 1.07E-087 | 14 | 14 | 12.65 | Cephamycin | Specific Drugs | *Nocardia lactamdurans* | Actino-bacteria | Bacteria |
| 2.A.1.3.8 | MFS | O54179 | SCO5950 | Q04733 | 2.51E-054 | 14 | 14 | 12.6 | Cephamycin | Specific Drugs | *Nocardia lactamdurans* | Actino-bacteria | Bacteria |
| 2.A.1.3.8 | MFS | Q9RDF5 | SCO2536 | Q04733 | 4.66E-047 | 14 | 14 | 9.9 | Cephamycin | Specific Drugs | *Nocardia lactamdurans* | Actino-bacteria | Bacteria |
| 2.A.1.3.10 | MFS | P11545 | SCO0295 | Q00538 | 2.43E-110 | 14 | 14 | 13.1 | Methylenomycin | Specific Drugs | *Bacillus subtilis* | Firmicutes | Bacteria |
| 2.A.1.3.11 | MFS | Q9FBQ5 | SCO4265 | P42670 | 3.16E-123 | 14 | 14 | 12.8 | Puromycin | Specific Drugs | *Streptomyces lipmanii* | Actino-bacteria | Bacteria |
| 2.A.1.3.11 | MFS | Q9KYU0 | SCO3206 | P42670 | 9.01E-113 | 14 | 14 | 12.4 | Puromycin | Specific Drugs | *Streptomyces lipmanii* | Actino-bacteria | Bacteria |
| 2.A.1.3.11 | MFS | O69986 | SCO5810 | P42670 | 1.38E-071 | 14 | 14 | 11.8 | Puromycin | Specific Drugs | *Streptomyces lipmanii* | Actino-bacteria | Bacteria |
| 2.A.1.3.11 | MFS | Q9RKU2 | SCO6250 | P42670 | 3.10E-005 | 4 | 14 | 3.35 | Puromycin | Specific Drugs | *Streptomyces lipmanii* | Actino-bacteria | Bacteria |
| 2.A.1.3.12 | MFS | Q8CJZ8 | SCO2373 | P39886 | 2.94E-178 | 13 | 13 | 12.65 | Tetracenomycin | Specific Drugs | *Streptomyces glaucescens* | Actino-bacteria | Bacteria |
| 2.A.1.3.12 | MFS | Q9RJY0 | SCO1190 | P39886 | 8.67E-112 | 12 | 13 | 9.25 | Tetracenomycin | Specific Drugs | *Streptomyces glaucescens* | Actino-bacteria | Bacteria |
| 2.A.1.3.12 | MFS | Q9L0L9 | SCO4641 | P39886 | 1.74E-063 | 14 | 13 | 9.9 | Tetracenomycin | Specific Drugs | *Streptomyces glaucescens* | Actino-bacteria | Bacteria |
| 2.A.1.3.12 | MFS | Q9X8R5 | SCO3648 | P39886 | 1.58E-053 | 14 | 13 | 10.5 | Tetracenomycin | Specific Drugs | *Streptomyces glaucescens* | Actino-bacteria | Bacteria |
| 2.A.1.3.12 | MFS | Q9KZX5 | SCO4451 | P39886 | 5.34E-041 | 14 | 13 | 10.8 | Tetracenomycin | Specific Drugs | *Streptomyces glaucescens* | Actino-bacteria | Bacteria |
| 2.A.1.3.12 | MFS | Q8CJZ9 | SCO2310 | P39886 | 5.56E-024 | 14 | 13 | 6.9 | Tetracenomycin | Specific Drugs | *Streptomyces glaucescens* | Actino-bacteria | Bacteria |
| 2.A.1.3.15 | MFS | Q9FC58 | SCO7128 | O52557 | 7.28E-045 | 14 | 14 | 10.15 | Rifamycin | Specific Drugs | *Amycolatopsis mediterranei* | Actino-bacteria | Bacteria |
| 2.A.1.3.19 | MFS | Q9L1B4 | SCO1567 | Q9L1B4 | 0 | 14 | 14 | 14.4 | Paraquot | Specific Drugs | *Streptomyces coelicolor* | Actino-bacteria | Bacteria |
| 2.A.1.3.19 | MFS | Q9EWX5 | SCO1751 | Q9L1B4 | 8.60E-088 | 14 | 14 | 13 | Paraquot | Specific Drugs | *Streptomyces coelicolor* | Actino-bacteria | Bacteria |
| 2.A.1.3.25 | MFS | P46105 | SCO5083 | P46105 | 0 | 14 | 14 | 14 | Actinorhodin | Specific Drugs | *Streptomyces coelicolor* | Actino-bacteria | Bacteria |
| 2.A.1.3.25 | MFS | Q9KYU6 | SCO3199 | P46105 | 8.28E-100 | 14 | 14 | 10.85 | Actinorhodin | Specific Drugs | *Streptomyces coelicolor* | Actino-bacteria | Bacteria |
| 2.A.1.3.25 | MFS | Q8CK49 | SCO0680 | P46105 | 5.24E-056 | 14 | 14 | 12.1 | Actinorhodin | Specific Drugs | *Streptomyces coelicolor* | Actino-bacteria | Bacteria |
| 2.A.1.3.28 | MFS | Q9Z563 | SCO5516 | Q939A4 | 0 | 12 | 12 | 12.05 | Multiple drugs | Multiple Drugs | *Streptomyces lividans* | Actino-bacteria | Bacteria |
| 2.A.1.3.29 | MFS | Q8CJY2 | SCO2937 | O69070 | 9.10E-017 | 14 | 14 | 9.4 | Tetracycline/oxytetracycline | Specific Drugs | *Streptomyces rimosus* | Actino-bacteria | Bacteria |
| 2.A.1.3.30 | MFS | Q9RJX6 | SCO1194 | O35018 | 3.26E-041 | 14 | 14 | 10.5 | Lincomycin | Specific Drugs | *Bacillus subtilis* | Firmicutes | Bacteria |
| 2.A.1.3.33 | MFS | Q93S11 | SCO6278 | A5H8A5 | 9.64E-092 | 14 | 16 | 11.75 | Hydrophilic quinolones, Norfloxacin, and ciprofloxacin | Multiple Drugs | *Escherichia coli* | Proteobacteria | Bacteria |
| 2.A.1.3.34 | MFS | Q9X8M3 | SCO3366 | Q9ZGB6 | 2.87E-136 | 14 | 14 | 12.9 | Landomycin | Specific Drugs | *Streptomyces cyanogenus* | Actino-bacteria | Bacteria |
| 2.A.1.3.34 | MFS | Q9KY46 | SCO2344 | Q9ZGB6 | 1.11E-101 | 14 | 14 | 12.75 | Landomycin | Specific Drugs | *Streptomyces cyanogenus* | Actino-bacteria | Bacteria |
| 2.A.1.3.34 | MFS | Q9ZBJ1 | SCO6483 | Q9ZGB6 | 1.61E-092 | 14 | 14 | 11.95 | Landomycin | Specific Drugs | *Streptomyces cyanogenus* | Actino-bacteria | Bacteria |
| 2.A.1.3.34 | MFS | Q9RD59 | SCO0623 | Q9ZGB6 | 2.35E-065 | 14 | 14 | 9.35 | Landomycin | Specific Drugs | *Streptomyces cyanogenus* | Actino-bacteria | Bacteria |
| 2.A.1.3.35 | MFS | Q9KZB9 | SCO6970 | O32182 | 4.78E-087 | 14 | 14 | 11.55 | Multiple drugs (novobiocin, streptomycin, and actinomycin D) | Multiple Drugs | *Bacillus subtilis* | Firmicutes | Bacteria |
| 2.A.1.3.35 | MFS | Q9ADM7 | SCO4031 | O32182 | 1.40E-066 | 14 | 14 | 11.25 | Multiple drugs (novobiocin, streptomycin, and actinomycin D) | Multiple Drugs | *Bacillus subtilis* | Firmicutes | Bacteria |
| 2.A.1.3.37 | MFS | O69916 | SCO6384 | D1QCY9 | 8.04E-019 | 6 | 15 | 3.65 | Methecillin | Specific Drugs | *Staphylococcus aureus* | Firmicutes | Bacteria |
| 2.A.1.3.37 | MFS | Q9X8H7 | SCO3397 | D1QCY9 | 9.11E-018 | 6 | 15 | 1.05 | Methecillin | Specific Drugs | *Staphylococcus aureus* | Firmicutes | Bacteria |
| 2.A.1.3.39 | MFS | O54176 | SCO5947 | A2RJJ9 | 8.72E-016 | 14 | 14 | 11.4 | Uridine/deoxyuridine/5-fluorouridine | Nucleic acids | *Lactococcus lactis subsp. cremoris* | Firmicutes | Bacteria |
| 2.A.1.3.40 | MFS | Q9L2I1 | SCO2502 | D9X7X8 | 0 | 14 | 14 | 13.65 | Unknown | Unknown | *Streptomyces viridochromogenes* | Actino-bacteria | Bacteria |
| 2.A.1.3.43 | MFS | Q9FCK7 | SCO5181 | O53781 | 3.34E-033 | 14 | 15 | 7.3 | Multiple drugs | Multiple Drugs | *Mycobacterium tuberculosis* | Actino-bacteria | Bacteria |
| 2.A.1.3.44 | MFS | Q9KZE6 | SCO7007 | O53781 | 1.63E-010 | 9 | 15 | 6.5 | Multiple drugs | Multiple Drugs | *Mycobacterium tuberculosis* | Actino-bacteria | Bacteria |
| 2.A.1.6.3 | MFS | Q9XAH4 | SCO6690 | Q52000 | 5.97E-082 | 12 | 12 | 10.75 | Dicarboxylate | Di- & tricarboxylates | *Pseudomonas putida* | Proteobacteria | Bacteria |
| 2.A.1.6.4 | MFS | Q9KXI4 | SCO2336 | P0C0L7 | 5.60E-137 | 12 | 12 | 11.5 | Proline/Glycine-betaine | Amino acids & conjugates | *Escherichia coli* | Proteobacteria | Bacteria |
| 2.A.1.6.4 | MFS | Q9L182 | SCO7395 | P0C0L7 | 2.65E-131 | 12 | 12 | 11.65 | Proline/Glycine-betaine | Amino acids & conjugates | *Escherichia coli* | Proteobacteria | Bacteria |
| 2.A.1.6.4 | MFS | Q9FC88 | SCO1225 | P0C0L7 | 2.89E-129 | 12 | 12 | 12.35 | Proline/Glycine-betaine | Amino acids & conjugates | *Escherichia coli* | Proteobacteria | Bacteria |
| 2.A.1.6.6 | MFS | Q9EX30 | SCO1010 | P76350 | 3.82E-086 | 12 | 12 | 10.7 | Shikimate | Monocarboxylates | *Escherichia coli* | Proteobacteria | Bacteria |
| 2.A.1.6.6 | MFS | O86563 | SCO5468 | P76350 | 2.27E-061 | 12 | 12 | 10 | Shikimate | Monocarboxylates | *Escherichia coli* | Proteobacteria | Bacteria |
| 2.A.1.8.11 | MFS | Q9L1T6 | SCO2959 | Q93PW1 | 2.31E-078 | 12 | 24 | 7.3 | Nitrate | Anions | *Paracoccus pantotrophus* | Proteobacteria | Bacteria |
| 2.A.1.8.11 | MFS | Q9RI35 | SCO0213 | Q93PW1 | 2.03E-038 | 12 | 24 | 10.65 | Nitrate | Anions | *Paracoccus pantotrophus* | Proteobacteria | Bacteria |
| 2.A.1.11.1 | MFS | O87836 | SCO6581 | Q51330 | 2.86E-019 | 12 | 11 | 7.5 | Oxylate | Anions | *Oxalobacter formigenes* | Proteobacteria | Bacteria |
| 2.A.1.11.1 | MFS | Q9ZBV5 | SCO6563 | Q51330 | 1.63E-017 | 12 | 11 | 6.45 | Oxylate | Anions | *Oxalobacter formigenes* | Proteobacteria | Bacteria |
| 2.A.1.11.2 | MFS | Q9F374 | SCO4414 | Q9I458 | 8.33E-030 | 12 | 12 | 9.6 | N/A | Nonspecific ion | *Pseudomonas aeruginosa* | Proteobacteria | Bacteria |
| 2.A.1.14.3 | MFS | Q9RJQ9 | SCO0581 | P70786 | 3.00E-094 | 12 | 12 | 8.25 | Tartrate | Di- & tricarboxylates | *Agrobacterium vitis* | Proteobacteria | Bacteria |
| 2.A.1.14.3 | MFS | Q9RDF0 | SCO2541 | P70786 | 9.53E-087 | 12 | 12 | 10.15 | Tartrate | Di- & tricarboxylates | *Agrobacterium vitis* | Proteobacteria | Bacteria |
| 2.A.1.14.7 | MFS | Q9RL05 | SCO0313 | P0AA76 | 3.80E-023 | 12 | 11 | 8.5 | D-Galactonate | Monocarboxylates | *Escherichia coli* | Proteobacteria | Bacteria |
| 2.A.1.15.5 | MFS | Q9L125 | SCO7435 | O30513 | 1.39E-046 | 12 | 12 | 9.9 | Benzoate | Monocarboxylates | *Acinetobacter calcoaceticus* | Proteobacteria | Bacteria |
| 2.A.1.15.9 | MFS | Q9RKX0 | SCO1411 | O51798 | 7.67E-076 | 12 | 12 | 11.05 | 4-methylmuconolactone | Monocarboxylates | *Ralstonia eutropha* | Proteobacteria | Bacteria |
| 2.A.1.15.9 | MFS | Q9RL01 | SCO0317 | O51798 | 5.32E-005 | 12 | 12 | 10.05 | 4-methylmuconolactone | Monocarboxylates | *Ralstonia eutropha* | Proteobacteria | Bacteria |
| 2.A.1.17.1 | MFS | Q9S277 | SCO1812 | P17583 | 1.68E-020 | 12 | 11 | 7.8 | Cyanate | Anions | *Escherichia coli* | Proteobacteria | Bacteria |
| 2.A.1.17.1 | MFS | Q93JG9 | SCO7703 | P17583 | 1.24E-017 | 12 | 11 | 10.7 | Cyanate | Anions | *Escherichia coli* | Proteobacteria | Bacteria |
| 2.A.1.17.1 | MFS | Q9FBT5 | SCO7157 | P17583 | 6.23E-007 | 6 | 11 | 5.55 | Cyanate | Anions | *Escherichia coli* | Proteobacteria | Bacteria |
| 2.A.1.21.3 | MFS | Q9L227 | SCO6805 | O31137 | 1.27E-024 | 12 | 12 | 11.3 | Tetracycline | Specific Drugs | *Mycobacterium smegmatis* | Actino-bacteria | Bacteria |
| 2.A.1.21.3 | MFS | Q9S2T9 | SCO2046 | O31137 | 6.86E-019 | 11 | 12 | 11.3 | Tetracycline | Specific Drugs | *Mycobacterium smegmatis* | Actino-bacteria | Bacteria |
| 2.A.1.21.3 | MFS | Q9X8T4 | SCO3898 | O31137 | 4.80E-006 | 12 | 12 | 10.15 | Tetracycline | Specific Drugs | *Mycobacterium smegmatis* | Actino-bacteria | Bacteria |
| 2.A.1.21.6 | MFS | Q9L102 | SCO1457 | Q46305 | 3.40E-008 | 12 | 12 | 8.25 | Tetracycline | Specific Drugs | *Clostridium perfringens* | Firmicutes | Bacteria |
| 2.A.1.21.8 | MFS | Q9KZY0 | SCO4446 | Q5WAS7 | 9.72E-005 | 12 | 12 | 10.2 | Multiple drugs (Macrolide) | Multiple Drugs | *Bacillus clausii* | Firmicutes | Bacteria |
| 2.A.1.21.9 | MFS | Q9X9Y0 | SCO1892 | Q9HLP1 | 4.85E-007 | 12 | 12 | 11 | Unknown | Unknown | *Thermoplasma acidophilum* | Euryarchaeota | Archaea |
| 2.A.1.24.1 | MFS | Q9RKX9 | SCO1420 | P25568 | 1.53E-027 | 13 | 11 | 6.35 | Unknown | Unknown | *Saccharomyces cerevisiae* | Ascomycota | Eukarya |
| 2.A.1.30.1 | MFS | Q9KXM0 | SCO4337 | Q9X4X4 | 7.17E-033 | 12 | 12 | 10.3 | Abietane | Specific Drugs | *Pseudomonas abietaniphila* | Proteobacteria | Bacteria |
| 2.A.1.30.1 | MFS | Q9X863 | SCO3507 | Q9X4X4 | 5.74E-027 | 12 | 12 | 9.55 | Abietane | Specific Drugs | *Pseudomonas abietaniphila* | Proteobacteria | Bacteria |
| 2.A.1.30.1 | MFS | Q9KYL8 | SCO7369 | Q9X4X4 | 3.45E-013 | 12 | 12 | 11.3 | Abietane | Specific Drugs | *Pseudomonas abietaniphila* | Proteobacteria | Bacteria |
| 2.A.1.30.1 | MFS | O69824 | SCO6430 | Q9X4X4 | 3.59E-013 | 8 | 12 | 6.5 | Abietane | Specific Drugs | *Pseudomonas abietaniphila* | Proteobacteria | Bacteria |
| 2.A.1.34.1 | MFS | Q9S243 | SCO1709 | Q7VWI9 | 1.34E-005 | 10 | 17 | 9.3 | Unknown | Unknown | *Bordetella pertussis* | Proteobacteria | Bacteria |
| 2.A.1.35.1 | MFS | Q9KYN5 | SCO7350 | P52067 | 5.59E-024 | 12 | 12 | 9.65 | Multiple drugs | Multiple Drugs | *Escherichia coli* | Proteobacteria | Bacteria |
| 2.A.1.36.1 | MFS | Q9AK30 | SCO4896 | P43531 | 4.23E-074 | 12 | 12 | 11 | Acriflavin | Specific Drugs | *Escherichia coli* | Proteobacteria | Bacteria |
| 2.A.1.36.2 | MFS | Q9ADP8 | SCO4007 | A8GHT9 | 1.02E-017 | 12 | 12 | 10.1 | Unknown | Unknown | *Serratia proteamaculans* | Proteobacteria | Bacteria |
| 2.A.1.38.2 | MFS | Q9RKR4 | SCO2264 | Q0E7C5 | 4.81E-039 | 12 | 12 | 10.3 | Siderophore | Siderophores; siderophores-Fe complexes | *Listonella anguillarum* | Proteobacteria | Bacteria |
| 2.A.1.38.2 | MFS | O69821 | SCO6427 | Q0E7C5 | 4.63E-030 | 12 | 12 | 10.35 | Siderophore | Siderophores; siderophores-Fe complexes | *Listonella anguillarum* | Proteobacteria | Bacteria |
| 2.A.1.38.2 | MFS | Q9K4K5 | SCO7309 | Q0E7C5 | 1.15E-023 | 12 | 12 | 11.15 | Siderophore | Siderophores; siderophores-Fe complexes | *Listonella anguillarum* | Proteobacteria | Bacteria |
| 2.A.1.40.2 | MFS | Q9KXM3 | SCO4334 | O34987 | 8.42E-069 | 13 | 12 | 8.05 | Hypoxanthine/guanosine | Nucleic acids | *Bacillus subtilis* | Firmicutes | Bacteria |
| 2.A.1.40.2 | MFS | Q9ZBN6 | SCO6173 | O34987 | 1.50E-063 | 13 | 12 | 8.5 | Hypoxanthine/guanosine | Nucleic acids | *Bacillus subtilis* | Firmicutes | Bacteria |
| 2.A.1.54.2 | MFS | Q9XA68 | SCO3809 | Q747F2 | 2.74E-023 | 12 | 12 | 9.85 | Unknown | Unknown | *Geobacter sulfurreducens* | Proteobacteria | Bacteria |
| 2.A.1.60.1 | MFS | Q9K432 | SCO1067 | Q07609 | 9.03E-032 | 12 | 12 | 8.9 | Rhizopine | Amines, amides, polyamines, & organocations | *Rhizobium meliloti* | Proteobacteria | Bacteria |
| 2.A.1.60.1 | MFS | Q9L111 | SCO1448 | Q07609 | 3.58E-031 | 12 | 12 | 9.2 | Rhizopine | Amines, amides, polyamines, & organocations | *Rhizobium meliloti* | Proteobacteria | Bacteria |
| 2.A.1.60.1 | MFS | O69867 | SCO6037 | Q07609 | 5.16E-030 | 12 | 12 | 7.8 | Rhizopine | Amines, amides, polyamines, & organocations | *Rhizobium meliloti* | Proteobacteria | Bacteria |
| 2.A.1.60.1 | MFS | Q93J00 | SCO7520 | Q07609 | 1.43E-026 | 12 | 12 | 7.55 | Rhizopine | Amines, amides, polyamines, & organocations | *Rhizobium meliloti* | Proteobacteria | Bacteria |
| 2.A.1.60.1 | MFS | Q9KYD1 | SCO6899 | Q07609 | 8.34E-023 | 12 | 12 | 9.65 | Rhizopine | Amines, amides, polyamines, & organocations | *Rhizobium meliloti* | Proteobacteria | Bacteria |
| 2.A.1.60.1 | MFS | Q9F2S7 | SCO4462 | Q07609 | 8.81E-022 | 12 | 12 | 7.85 | Rhizopine | Amines, amides, polyamines, & organocations | *Rhizobium meliloti* | Proteobacteria | Bacteria |
| 2.A.1.60.1 | MFS | Q9WX03 | SCO3331 | Q07609 | 1.20E-017 | 12 | 12 | 7.7 | Rhizopine | Amines, amides, polyamines, & organocations | *Rhizobium meliloti* | Proteobacteria | Bacteria |
| 2.A.1.67.1 | MFS | Q9KYV9 | SCO3186 | D2PP09 | 9.36E-049 | 12 | 12 | 10.65 | Unknown | Unknown | *Kribbella flavida* | Actino-bacteria | Bacteria |
| 2.A.1.67.1 | MFS | O50532 | SCO5858 | D2PP09 | 4.25E-048 | 11 | 12 | 9.7 | Unknown | Unknown | *Kribbella flavida* | Actino-bacteria | Bacteria |
| 2.A.1.67.1 | MFS | Q9L1T0 | SCO2965 | D2PP09 | 2.89E-039 | 11 | 12 | 10 | Unknown | Unknown | *Kribbella flavida* | Actino-bacteria | Bacteria |
| 2.A.1.67.1 | MFS | Q9RJ60 | SCO1641 | D2PP09 | 1.09E-027 | 12 | 12 | 11.05 | Unknown | Unknown | *Kribbella flavida* | Actino-bacteria | Bacteria |
| 2.A.1.67.1 | MFS | Q9L2B9 | SCO5401 | D2PP09 | 1.15E-020 | 12 | 12 | 8.8 | Unknown | Unknown | *Kribbella flavida* | Actino-bacteria | Bacteria |
| 2.A.3.1.5 | APC | Q9L205 | SCO2628 | P46349 | 2.01E-124 | 9 | 12 | 7.7 | B-alanine/ y-aminobutyrate | Amino acids & conjugates | *Bacillus subtilis* | Firmicutes | Bacteria |
| 2.A.3.1.5 | APC | Q9L202 | SCO2631 | P46349 | 1.34E-109 | 12 | 12 | 10.35 | B-alanine/ y-aminobutyrate | Amino acids & conjugates | *Bacillus subtilis* | Firmicutes | Bacteria |
| 2.A.3.1.7 | APC | Q9S2G7 | SCO2914 | P0AAE0 | 3.63E-113 | 12 | 12 | 10.4 | D-serine/D-alanine/glycine | Amino acids & conjugates | *Escherichia coli* | Proteobacteria | Bacteria |
| 2.A.3.1.8 | APC | Q9X7P0 | SCO6734 | P40812 | 1.20E-153 | 12 | 12 | 10.75 | Asparagine | Amino acids & conjugates | *Salmonella typhimurium* | Proteobacteria | Bacteria |
| 2.A.3.1.13 | APC | Q9FBM2 | SCO5057 | P76037 | 2.25E-046 | 12 | 12 | 9.2 | Putrescine | Amino acids & conjugates | *Escherichia coli* | Proteobacteria | Bacteria |
| 2.A.3.2.5 | APC | O86710 | SCO6528 | P60061 | 1.98E-061 | 12 | 12 | 10.55 | Arginine/agmatine | Amino acids & conjugates | *Escherichia coli* | Proteobacteria | Bacteria |
| 2.A.3.3.1 | APC | Q9RCX2 | SCO0938 | Q09143 | 2.72E-059 | 12 | 14 | 6.1 | Arginine, lysine and ornithine | Amino acids & conjugates | *Mus musculus* | Chordata | Eukarya |
| 2.A.3.3.3 | APC | O69844 | SCO6014 | Q96241 | 2.10E-070 | 12 | 14 | 7.5 | Nonspecific | Amino acids & conjugates | *Arabidopsis thaliana* | Angiosperms | Eukarya |
| 2.A.3.3.3 | APC | Q9L100 | SCO1459 | Q96241 | 6.67E-049 | 12 | 14 | 10.25 | Nonspecific | Amino acids & conjugates | *Arabidopsis thaliana* | Angiosperms | Eukarya |
| 2.A.3.4.3 | APC | Q8CJU9 | SCO4097 | P32837 | 2.43E-058 | 12 | 12 | 9.25 | Polyamines/y-aminobutyric acid (GABA) | Amino acids & conjugates | *Saccharomyces cerevisiae* | Ascomycota | Eukarya |
| 2.A.3.4.3 | APC | Q9KZF1 | SCO7002 | P32837 | 3.05E-050 | 12 | 12 | 7.95 | Polyamines/y-aminobutyric acid (GABA) | Amino acids & conjugates | *Saccharomyces cerevisiae* | Ascomycota | Eukarya |
| 2.A.3.6.1 | APC | Q93JF0 | SCO5977 | O28661 | 7.68E-048 | 12 | 13 | 10.25 | Nonspecific Cationic AA | Amino acids & conjugates | *Archaeoglobus fulgidus* | Euryarchaeota | Archaea |
| 2.A.3.6.1 | APC | Q9F2U9 | SCO4612 | O28661 | 5.13E-034 | 10 | 13 | 6.5 | Nonspecific Cationic AA | Amino acids & conjugates | *Archaeoglobus fulgidus* | Euryarchaeota | Archaea |
| 2.A.3.8.7 | APC | Q9L1J2 | SCO2724 | Q9R0S5 | 3.56E-014 | 12 | 12 | 10.75 | Neutral amino acids/basic amino acids/neutrotoxicant/methylmercury-L-cysteine | Amino acids & conjugates | *Not Stated* |  |  |
| 2.A.3.14.1 | APC | O54098 | SCO5900 | A4X503 | 0 | 12 | 12 | 10.9 | Unknown | Unknown | *Salinispora tropica* | Actino-bacteria | Bacteria |
| 2.A.3.14.1 | APC | O69917 | SCO6385 | A4X503 | 1.06E-081 | 12 | 12 | 8.25 | Unknown | Unknown | *Salinispora tropica* | Actino-bacteria | Bacteria |
| 2.A.3.15.4 | APC | Q9S269 | SCO1683 | P76037 | 4.19E-007 | 12 | 12 | 8.4 | Putrescine | Amino acids & conjugates | *Escherichia coli* | Proteobacteria | Bacteria |
| 2.A.4.1.1 | CDF | Q9X7Q7 | SCO6751 | P13512 | 5.05E-062 | 5 | 5 | 1.75 | Cd^2+^, Zn^2+^, Co^2+^ | Cations | *Alcaligenes eutrophus* | Proteobacteria | Bacteria |
| 2.A.4.6.1 | CDF | Q9KZM0 | SCO3024 | Q6PML9 | 7.01E-035 | 5 | 5 | 4.6 | Zn^2+^ | Cations | *Homo sapiens* | Chordata | Eukarya |
| 2.A.4.6.1 | CDF | Q9L081 | SCO2772 | Q6PML9 | 1.58E-031 | 5 | 5 | 4.3 | Zn^2+^ | Cations | *Homo sapiens* | Chordata | Eukarya |
| 2.A.4.6.1 | CDF | Q9EWS3 | SCO0776 | Q6PML9 | 1.10E-030 | 6 | 5 | 4.25 | Zn^2+^ | Cations | *Homo sapiens* | Chordata | Eukarya |
| 2.A.4.7.1 | CDF | Q93IW0 | SCO1310 | P69380 | 5.25E-016 | 6 | 6 | 5 | Fe^2+^ | Cations | *Escherichia coli* | Proteobacteria | Bacteria |
| 2.A.6.3.1 | RND | Q8CK05 | SCO2164 | P25197 | 5.93E-108 | 11 | 12 | 8.65 | Lipooligosaccharide | Lipids | *Rhizobium meliloti* | Proteobacteria | Bacteria |
| 2.A.6.4.1 | RND | Q9ZBS8 | SCO6160 | P0AG90 | 1.49E-057 | 10 | 6 | 3.45 | Unknown | Unknown | *Escherichia coli* | Proteobacteria | Bacteria |
| 2.A.6.5.1 | RND | Q53902 | SCO5084 | Q53902 | 0 | 9 | 9 | 10.6 | Actinorhodin | Specific Drugs | *Streptomyces coelicolor* | Actino-bacteria | Bacteria |
| 2.A.6.5.1 | RND | Q9XA86 | SCO0839 | Q53902 | 3.46E-142 | 12 | 9 | 7.7 | Actinorhodin | Specific Drugs | *Streptomyces coelicolor* | Actino-bacteria | Bacteria |
| 2.A.6.5.1 | RND | Q9KZ54 | SCO7077 | Q53902 | 3.53E-134 | 11 | 9 | 8.5 | Actinorhodin | Specific Drugs | *Streptomyces coelicolor* | Actino-bacteria | Bacteria |
| 2.A.6.5.1 | RND | Q9L2G4 | SCO2519 | Q53902 | 1.12E-129 | 11 | 9 | 7 | Actinorhodin | Specific Drugs | *Streptomyces coelicolor* | Actino-bacteria | Bacteria |
| 2.A.6.5.1 | RND | O54101 | SCO5905 | Q53902 | 6.35E-085 | 14 | 9 | 6.9 | Actinorhodin | Specific Drugs | *Streptomyces coelicolor* | Actino-bacteria | Bacteria |
| 2.A.6.5.1 | RND | Q9RD06 | SCO0905 | Q53902 | 1.91E-013 | 2 | 9 | 2.1 | Actinorhodin | Specific Drugs | *Streptomyces coelicolor* | Actino-bacteria | Bacteria |
| 2.A.6.5.5 | RND | Q9L092 | SCO2454 | O53657 | 3.06E-085 | 12 | 12 | 6.6 | Heme | Aromatic compounds | *Mycobacterium tuberculosis* | Actino-bacteria | Bacteria |
| 2.A.6.5.5 | RND | O88022 | SCO6666 | O53657 | 3.31E-070 | 11 | 12 | 4.8 | Heme | Aromatic compounds | *Mycobacterium tuberculosis* | Actino-bacteria | Bacteria |
| 2.A.6.5.5 | RND | Q9RKC1 | SCO3166 | O53657 | 1.22E-054 | 12 | 12 | 6.4 | Heme | Aromatic compounds | *Mycobacterium tuberculosis* | Actino-bacteria | Bacteria |
| 2.A.6.5.5 | RND | Q9KYZ3 | SCO7536 | O53657 | 1.32E-054 | 12 | 12 | 6.25 | Heme | Aromatic compounds | *Mycobacterium tuberculosis* | Actino-bacteria | Bacteria |
| 2.A.6.5.5 | RND | Q9ADH2 | SCO6091 | O53657 | 2.08E-051 | 11 | 12 | 6.8 | Heme | Aromatic compounds | *Mycobacterium tuberculosis* | Actino-bacteria | Bacteria |
| 2.A.6.5.5 | RND | Q9RL63 | SCO0423 | O53657 | 6.61E-051 | 12 | 12 | 10.55 | Heme | Aromatic compounds | *Mycobacterium tuberculosis* | Actino-bacteria | Bacteria |
| 2.A.6.5.5 | RND | Q9KZ42 | SCO7090 | O53657 | 2.21E-050 | 12 | 12 | 6.5 | Heme | Aromatic compounds | *Mycobacterium tuberculosis* | Actino-bacteria | Bacteria |
| 2.A.6.5.5 | RND | Q9Z577 | SCO5502 | O53657 | 1.57E-048 | 12 | 12 | 7.5 | Heme | Aromatic compounds | *Mycobacterium tuberculosis* | Actino-bacteria | Bacteria |
| 2.A.7.1.3 | DMT | Q9X9V4 | SCO1918 | P23895 | 9.67E-022 | 3 | 4 | 2.7 | Multiple drugs | Multiple Drugs | *Escherichia coli* | Proteobacteria | Bacteria |
| 2.A.7.1.4 | DMT | Q9K4A9 | SCO5210 | P69937 | 7.72E-022 | 4 | 4 | 3.55 | Quaternary Ammonium | Multiple Drugs | *Escherichia coli* | Proteobacteria | Bacteria |
| 2.A.7.1.10 | DMT | Q9KZQ5 | SCO2898 | D5CES3 | 4.71E-028 | 4 | 4 | 3.65 | Multiple drugs | Multiple Drugs | *Enterobacter cloacae* | Proteobacteria | Bacteria |
| 2.A.7.3.2 | DMT | Q9RDH1 | SCO2756 | P31125 | 1.58E-048 | 10 | 10 | 8.6 | Amino acid metabolite | Amino acids & conjugates | *Escherichia coli* | Proteobacteria | Bacteria |
| 2.A.7.3.3 | DMT | Q9L1Y9 | SCO2646 | P42194 | 1.08E-053 | 10 | 9 | 7.55 | Indigoidine (blue pigment) | Pigments | *Erwinia chrysanthemi* | Proteobacteria | Bacteria |
| 2.A.7.3.4 | DMT | Q9AKA0 | SCO1362 | P39649 | 1.42E-009 | 10 | 10 | 5.5 | Drug/metabolite | Multiple Drugs | *Bacillus subtilis* | Firmicutes | Bacteria |
| 2.A.7.3.6 | DMT | Q9K3X0 | SCO4374 | P0AA67 | 7.34E-048 | 10 | 10 | 8.55 | Thronine/Homoserine (proline, serine, histidine, and other amino acids analogues) | Amino acids & conjugates | *Escherichia coli* | Proteobacteria | Bacteria |
| 2.A.7.3.11 | DMT | Q8CK31 | SCO1359 | A4FP84 | 1.21E-039 | 10 | 10 | 8.5 | Drug/metabolite | Multiple Drugs | *Saccharopolyspora erythraea* | Actino-bacteria | Bacteria |
| 2.A.7.3.11 | DMT | Q9L020 | SCO2293 | A4FP84 | 1.33E-014 | 10 | 10 | 7.8 | Drug/metabolite | Multiple Drugs | *Saccharopolyspora erythraea* | Actino-bacteria | Bacteria |
| 2.A.7.3.11 | DMT | Q9S248 | SCO1704 | A4FP84 | 1.98E-014 | 10 | 10 | 7.5 | Drug/metabolite | Multiple Drugs | *Saccharopolyspora erythraea* | Actino-bacteria | Bacteria |
| 2.A.7.3.14 | DMT | Q9L183 | SCO7394 | D8D9B1 | 0.000139755 | 10 | 10 | 3.7 | Drug/metabolite | Multiple Drugs | *Comamonas testosteroni* | Proteobacteria | Bacteria |
| 2.A.7.7.1 | DMT | Q9F2W9 | SCO4592 | O68827 | 8.97E-025 | 10 | 10 | 8.75 | Chloramphenicol | Specific Drugs | *Pseudomonas aeruginosa* | Proteobacteria | Bacteria |
| 2.A.7.18.1 | DMT | O50525 | SCO5851 | P14182 | 0.00087613 | 10 | 10 | 8.3 | Choline | Vitamins & vitamin or cofactor precursors | *Haemophilus influenzae* | Proteobacteria | Bacteria |
| 2.A.7.23.2 | DMT | Q9AK99 | SCO1363 | Q8YUK6 | 6.03E-009 | 10 | 10 | 7.75 | Tryptophan | Amino acids & conjugates | *Anabaena sp.* | Cyanobacteria | Bacteria |
| 2.A.7.24.5 | DMT | Q9L1Z0 | SCO2645 | Q4FKW8 | 8.30E-023 | 10 | 10 | 7.15 | Thiamine Pyrophosphate | Vitamins & vitamin or cofactor precursors | *Pelagibacter ubique* | Proteobacteria | Bacteria |
| 2.A.7.25.5 | DMT | Q9RIT4 | SCO0973 | Q9LIR9 | 6.01E-005 | 9 | 9 | 5.55 | Mg^2+^ | Cations | *Arabidopsis thaliana* | Angiosperms | Eukarya |
| 2.A.7.26.4 | DMT | Q9X889 | SCO3297 | A9T501 | 1.23E-018 | 4 | 4 | 3.6 | Multiple drugs | Multiple Drugs | *Physcomitrella patens* | Bryophyta | Eukarya |
| 2.A.8.1.1 | GntP | Q93JK3 | SCO4991 | P12012 | 1.39E-079 | 13 | 11 | 7.35 | D-Gluconate | Monocarboxylates | *Bacillus subtilis* | Firmicutes | Bacteria |
| 2.A.8.1.4 | GntP | Q8CK22 | SCO1680 | P39835 | 5.17E-093 | 12 | 11 | 9.65 | D-Gluconate | Monocarboxylates | *Escherichia coli* | Proteobacteria | Bacteria |
| 2.A.9.2.1 | Oxa1 | O54569 | SCO3883 | Q8LBP4 | 1.18E-026 | 3 | 6 | 2.5 | Electrons | Electrons | *Not Stated* |  |  |
| 2.A.9.2.1 | Oxa1 | Q9RDA0 | SCO2851 | Q8LBP4 | 6.69E-013 | 4 | 6 | 3.5 | Electrons | Electrons | *Not Stated* |  |  |
| 2.A.11.1.4 | CitMHS | Q9S242 | SCO1710 | Q9S242 | 0 | 9 | 9 | 8.5 | Fe^2+^, Ca^2+^, Pb^2+^, Ba^2+^, Mn^2+^ | Cations | *Streptomyces coelicolor* | Actino-bacteria | Bacteria |
| 2.A.14.1.2 | LctP | Q9KZL5 | SCO3029 | Q46839 | 4.52E-104 | 14 | 18 | 12.05 | L-lactate, D-lactate, glycolate | Monocarboxylates | *Escherichia coli* | Proteobacteria | Bacteria |
| 2.A.15.1.7 | BCCT | Q9X7P5 | SCO6739 | Q8NN75 | 4.89E-130 | 12 | 12 | 11.45 | Choline-glycine betaine | Amino acids & conjugates | *Corynebacterium glutamicum* | Actino-bacteria | Bacteria |
| 2.A.16.4.2 | TDT | O88074 | SCO1617 | A3R044 | 6.57E-023 | 10 | 10 | 8.65 | Sulphite | Anions | *Arthroderma benhamiae* | Ascomycota | Eukarya |
| 2.A.17.1.1 | POT | Q9KZ81 | SCO3064 | P0C2U2 | 3.34E-097 | 14 | 13 | 9.1 | Di-/tripeptide | Peptides | *Streptococcus lactis* | Firmicutes | Bacteria |
| 2.A.19.1.1 | CaCA | Q9Z504 | SCO1962 | P31801 | 3.09E-045 | 11 | 11 | 8.9 | Ca^2+^ | Cations | *Escherichia coli* | Proteobacteria | Bacteria |
| 2.A.20.1.1 | PiT | Q9KZW3 | SCO4138 | P0AFJ7 | 1.30E-027 | 8 | 10 | 4.8 | Inorganic phosphate | Anions | *Escherichia coli* | Proteobacteria | Bacteria |
| 2.A.20.1.2 | PiT | Q9RJ23 | SCO1845 | P43676 | 8.66E-022 | 11 | 10 | 4.5 | Inorganic phosphate | Anions | *Escherichia coli* | Proteobacteria | Bacteria |
| 2.A.21.3.2 | SSS | Q9K3S5 | SCO3139 | P96169 | 4.14E-046 | 14 | 14 | 10.2 | Glucose/galactose | Sugars & polyols | *Vibrio parahaemolyticus* | Proteobacteria | Bacteria |
| 2.A.21.3.7 | SSS | Q9EWI8 | SCO7596 | Q5E733 | 4.29E-037 | 13 | 13 | 10.25 | Sialic acid | Anions | *Vibrio fischeri* | Proteobacteria | Bacteria |
| 2.A.21.3.7 | SSS | Q9KZY9 | SCO4437 | Q5E733 | 4.58E-019 | 13 | 13 | 10.1 | Sialic acid | Anions | *Vibrio fischeri* | Proteobacteria | Bacteria |
| 2.A.21.4.1 | SSS | Q9K494 | SCO5229 | Q8VM88 | 1.27E-132 | 13 | 13 | 9.2 | Lactate, pyruvate, propionate, butyrate, a-hydroxybutylate, L- and D- alanine (possibly cysteine and histidine) | Monocarboxylates | *Rhizobium leguminosarum* | Proteobacteria | Bacteria |
| 2.A.21.7.3 | SSS | Q9RJ46 | SCO1822 | Q8NS49 | 8.43E-160 | 13 | 13 | 11.45 | Pyruvate/Acetate/Propionate | Monocarboxylates | *Corynebacterium glutamicum* | Actino-bacteria | Bacteria |
| 2.A.21.7.3 | SSS | O70003 | SCO5827 | Q8NS49 | 2.11E-134 | 13 | 13 | 11.95 | Pyruvate/Acetate/Propionate | Monocarboxylates | *Corynebacterium glutamicum* | Actino-bacteria | Bacteria |
| 2.A.21.7.3 | SSS | Q8CK67 | SCO0152 | Q8NS49 | 8.70E-036 | 13 | 13 | 11.95 | Pyruvate/Acetate/Propionate | Monocarboxylates | *Corynebacterium glutamicum* | Actino-bacteria | Bacteria |
| 2.A.21.7.3 | SSS | Q9FC95 | SCO1218 | Q8NS49 | 1.02E-031 | 13 | 13 | 9.15 | Pyruvate/Acetate/Propionate | Monocarboxylates | *Corynebacterium glutamicum* | Actino-bacteria | Bacteria |
| 2.A.23.1.3 | DAACS | Q9L1K8 | SCO5436 | Q01857 | 7.17E-099 | 8 | 11 | 7.85 | Fumarate, D- and L- malate, succinate, succinamide, orotate, iticonate, mesaconate (C4-dicarboxylate) | Di- & tricarboxylates | *Rhizobium leguminosarum* | Proteobacteria | Bacteria |
| 2.A.23.1.5 | DAACS | Q9L0U6 | SCO4498 | O59010 | 1.36E-052 | 8 | 9 | 6.25 | Glutamate | Amino acids & conjugates | *Pyrococcus horikoshii* | Euryarchaeota | Archaea |
| 2.A.25.1.3 | AGCS | Q9FBY5 | SCO7197 | Q6LX42 | 2.80E-073 | 11 | 11 | 6.6 | Alanine/ Na^+^ | Amino acids & conjugates | *Methanococcus maripaludis* | Euryarchaeota | Archaea |
| 2.A.28.1.2 | BASS | O86662 | SCO6370 | Q12908 | 0.000481866 | 9 | 9 | 5.85 | Bile acid | Organoanions (noncarboxylic) | *Homo sapiens* | Chordata | Eukarya |
| 2.A.28.2.1 | BASS | Q9KZG2 | SCO6990 | Q3EA49 | 2.32E-036 | 10 | 10 | 8.4 | Glucosinolate | Amino acids & conjugates | *Arabidopsis thaliana* | Angiosperms | Eukarya |
| 2.A.33.1.2 | NhaA | Q9X929 | SCO3564 | Q56725 | 3.89E-067 | 10 | 10 | 6.75 | Cation (Na^+^, H^+^) | Cations | *Vibrio parahaemolyticus* | Proteobacteria | Bacteria |
| 2.A.33.1.2 | NhaA | Q9S1X8 | SCO7832 | Q56725 | 1.55E-055 | 10 | 10 | 7.3 | Cation (Na^+^, H^+^) | Cations | *Vibrio parahaemolyticus* | Proteobacteria | Bacteria |
| 2.A.33.1.2 | NhaA | Q9S2C8 | SCO0285 | Q56725 | 5.33E-048 | 10 | 10 | 6.8 | Cation (Na^+^, H^+^) | Cations | *Vibrio parahaemolyticus* | Proteobacteria | Bacteria |
| 2.A.36.3.1 | CPA1 | Q9F3L8 | SCO5246 | P32703 | 1.32E-046 | 12 | 13 | 8.5 | Cation | Cations | *Escherichia coli* | Proteobacteria | Bacteria |
| 2.A.36.3.2 | CPA1 | Q9S2Y0 | SCO2071 | O32212 | 1.70E-047 | 13 | 12 | 10.55 | Na^+^, K^+^, Li^+^, Rb^+^, H^+^ | Cations | *Bacillus subtilis* | Firmicutes | Bacteria |
| 2.A.36.6.4 | CPA1 | Q9KYW0 | SCO3185 | Q0ZAH6 | 1.07E-076 | 13 | 13 | 10.6 | K^+^, NH_4_^+^ | Cations | *Alkalimonaamylolytica* | Proteobacteria | Bacteria |
| 2.A.36.6.4 | CPA1 | Q9XAJ9 | SCO3603 | Q0ZAH6 | 3.73E-076 | 13 | 13 | 10.4 | K^+^, NH_4_^+^ | Cations | *Alkalimonaamylolytica* | Proteobacteria | Bacteria |
| 2.A.37.2.3 | CPA2 | Q9X8F2 | SCO3279 | Q45308 | 2.46E-013 | 13 | 13 | 10.25 | Cation? (Na^+^, K^+^, H^+^) | Cations | *Bacillus megaterium* | Firmicutes | Bacteria |
| 2.A.37.4.2 | CPA2 | Q93JF3 | SCO5974 | Q9SUQ7 | 6.02E-042 | 11 | 13 | 9.3 | Cation, H^+^ | Cations | *Arabidopsis thaliana* | Angiosperms | Eukarya |
| 2.A.37.5.2 | CPA2 | Q9L191 | SCO7384 | O07536 | 3.18E-066 | 13 | 13 | 9.9 | K^+^, H^+^ | Cations | *Bacillus subtilis* | Firmicutes | Bacteria |
| 2.A.37.5.2 | CPA2 | O69958 | SCO5782 | O07536 | 1.46E-043 | 13 | 13 | 9.6 | K^+^, H^+^ | Cations | *Bacillus subtilis* | Firmicutes | Bacteria |
| 2.A.37.5.2 | CPA2 | Q9XAK0 | SCO3602 | O07536 | 4.19E-040 | 13 | 13 | 9.7 | K^+^, H^+^ | Cations | *Bacillus subtilis* | Firmicutes | Bacteria |
| 2.A.39.1.1 | NCS1 | Q93RZ8 | SCO0572 | P0AA82 | 1.06E-026 | 12 | 12 | 9.1 | Cytosine | Nucleic acids | *Escherichia coli* | Proteobacteria | Bacteria |
| 2.A.39.2.4 | NCS1 | Q9ZBQ0 | SCO5579 | B1PXD0 | 1.89E-006 | 12 | 12 | 9.15 | Cytosine-purine | Nucleic acids | *Emericella nidulans* | Ascomycota | Eukarya |
| 2.A.39.2.4 | NCS1 | O86506 | SCO5524 | B1PXD0 | 4.98E-005 | 12 | 12 | 8.85 | Cytosine-purine | Nucleic acids | *Emericella nidulans* | Ascomycota | Eukarya |
| 2.A.39.3.4 | NCS1 | O69811 | SCO6417 | P94575 | 5.57E-111 | 12 | 12 | 9.95 | Allantoin | Aromatic compounds | *Bacillus subtilis* | Firmicutes | Bacteria |
| 2.A.39.3.4 | NCS1 | Q93J97 | SCO7500 | P94575 | 4.32E-071 | 12 | 12 | 8.7 | Allantoin | Aromatic compounds | *Bacillus subtilis* | Firmicutes | Bacteria |
| 2.A.40.1.3 | NCS2 | Q9L109 | SCO1450 | P75892 | 8.58E-087 | 12 | 14 | 8.65 | Pyrimidine | Aromatic compounds | *Escherichia coli* | Proteobacteria | Bacteria |
| 2.A.40.3.1 | NCS2 | Q9RKW2 | SCO6214 | P42086 | 2.29E-084 | 12 | 13 | 8.8 | Xanthine | Aromatic compounds | *Bacillus subtilis* | Firmicutes | Bacteria |
| 2.A.40.3.1 | NCS2 | Q9RKW4 | SCO6212 | P42086 | 2.43E-081 | 12 | 13 | 8.45 | Xanthine | Aromatic compounds | *Bacillus subtilis* | Firmicutes | Bacteria |
| 2.A.45.1.1 | ArsB | Q9KYM0 | SCO7367 | P30329 | 1.03E-024 | 11 | 11 | 6.75 | Arsenical | Anions | *Staphylococcus aureus* | Firmicutes | Bacteria |
| 2.A.47.1.6 | DASS | Q9X803 | SCO2144 | Q8LG88 | 7.79E-011 | 13 | 12 | 6.8 | Malate (dicarboxylate) | Di- & tricarboxylates | *Not Stated* |  |  |
| 2.A.49.6.1 | Amt | Q93RS4 | SCO6320 | P74477 | 8.92E-036 | 10 | 11 | 9 | Cl^-^ | Anions | *Synechocystis sp.* | Cyanobacteria | Bacteria |
| 2.A.53.3.1 | SulP | Q9X8E9 | SCO3276 | O07488 | 3.60E-132 | 13 | 13 | 7.95 | Sulfate | Anions | *Yersinia enterocolitica* | Proteobacteria | Bacteria |
| 2.A.53.3.8 | SulP | Q8CJP5 | SCO6054 | Q8F8H7 | 3.97E-053 | 8 | 10 | 4.3 | Bicarbonate | Anions | *Leptospira interrogans* | Spirochaetes | Bacteria |
| 2.A.53.3.8 | SulP | Q9X927 | SCO3562 | Q8F8H7 | 1.32E-048 | 9 | 10 | 5 | Bicarbonate | Anions | *Leptospira interrogans* | Spirochaetes | Bacteria |
| 2.A.55.1.3 | Nramp | Q93JK1 | SCO4993 | Q12078 | 2.98E-005 | 11 | 11 | 5.75 | Fe^2+^/ Mn^2+^ | Cations | *Saccharomyces cerevisiae* | Ascomycota | Eukarya |
| 2.A.59.1.2 | ACR3 | Q9X8Y0 | SCO3698 | P45946 | 4.39E-102 | 10 | 10 | 8.9 | Arsenite/antimonite | Cations | *Bacillus subtilis* | Firmicutes | Bacteria |
| 2.A.59.1.2 | ACR3 | Q9L1X4 | SCO6837 | P45946 | 6.16E-102 | 10 | 10 | 8.9 | Arsenite/antimonite | Cations | *Bacillus subtilis* | Firmicutes | Bacteria |
| 2.A.63.1.1 | CPA3 | Q9KZD5 | SCO6954 | Q52978 | 1.13E-103 | 25 | 25 | 17.35 | K^+^/Na^+^/H^+^ | Cations | *Rhizobium meliloti* | Proteobacteria | Bacteria |
| 2.A.63.1.1 | CPA3 | Q9KZD1 | SCO6958 | Q52983 | 1.27E-005 | 3 | 3 | 2.6 | K^+^/Na^+^/H^+^ | Cations | *Rhizobium meliloti* | Proteobacteria | Bacteria |
| 2.A.63.1.4 | CPA3 | Q9KZD3 | SCO6956 | O05229 | 1.77E-068 | 15 | 14 | 10.65 | Na^+^/H^+^ | Cations | *Bacillus subtilis* | Firmicutes | Bacteria |
| 2.A.63.1.4 | CPA3 | Q9KZD0 | SCO6959 | O05227 | 8.73E-016 | 3 | 3 | 2.15 | Na^+^/H^+^ | Cations | *Bacillus subtilis* | Firmicutes | Bacteria |
| 2.A.63.1.4 | CPA3 | Q9KZD4 | SCO6955 | O05260 | 2.11E-015 | 3 | 3 | 2.85 | Na^+^/H^+^ | Cations | *Bacillus subtilis* | Firmicutes | Bacteria |
| 2.A.64.1.1 | Tat | Q9FBK8 | SCO5150 | P69425 | 1.96E-006 | 1 | 1 | 0.85 | Arginine | Amino acids & conjugates | *Escherichia coli* | Proteobacteria | Bacteria |
| 2.A.64.2.1 | Tat | Q9RJ69 | SCO1632 | Q9SJV5 | 2.02E-026 | 6 | 6 | 3.2 | Arginine | Amino acids & conjugates | *Arabidopsis thaliana* | Angiosperms | Eukarya |
| 2.A.64.2.1 | Tat | Q9RJ68 | SCO1633 | Q9LKU2 | 5.32E-008 | 1 | 1 | 0.85 | Arginine | Amino acids & conjugates | *Arabidopsis thaliana* | Angiosperms | Eukarya |
| 2.A.66.1.4 | MOP | Q9X8U6 | SCO3910 | P28303 | 2.74E-023 | 12 | 13 | 10.25 | DNA | Nucleic acids | *Escherichia coli* | Proteobacteria | Bacteria |
| 2.A.66.4.1 | MOP | Q9KYG3 | SCO2709 | P37169 | 5.22E-027 | 13 | 14 | 10.6 | Lipid II peptidoglycan | Lipids | *Salmonella typhimurium* | Proteobacteria | Bacteria |
| 2.A.66.4.1 | MOP | Q9X8T0 | SCO3894 | P37169 | 1.53E-022 | 14 | 14 | 7.25 | Lipid II peptidoglycan | Lipids | *Salmonella typhimurium* | Proteobacteria | Bacteria |
| 2.A.69.2.1 | AEC | Q9AK33 | SCO4893 | O32715 | 0.000435673 | 10 | 10 | 7.55 | Malonate | Amino acids & conjugates | *Klebsiella pneumoniae* | Proteobacteria | Bacteria |
| 2.A.75.1.2 | LysE | Q9K4K6 | SCO7308 | P11667 | 2.25E-025 | 6 | 6 | 5.55 | L-arginine | Amino acids & conjugates | *Escherichia coli* | Proteobacteria | Bacteria |
| 2.A.76.1.1 | RhtB | Q9X8L9 | SCO3362 | P0AG34 | 4.18E-011 | 6 | 6 | 5.85 | Homoserine lactone | Amino acids & conjugates | *Escherichia coli* | Proteobacteria | Bacteria |
| 2.A.78.2.1 | LIV-E | O50481 | SCO5763 | A4EZB2 | 1.42E-009 | 4 | 7 | 3.85 | L-methionine, isoleucine, leucine, valine | Amino acids & conjugates | *Roseobacter sp* | Proteobacteria | Bacteria |
| 2.A.79.1.1 | ThrE | Q9X8J0 | SCO3410 | Q79VD1 | 7.40E-026 | 10 | 11 | 7.65 | Threonine | Amino acids & conjugates | *Not Stated* |  |  |
| 2.A.80.1.1 | TTT | Q9EWY6 | SCO1140 | Q9FA44 | 5.61E-097 | 12 | 13 | 10.15 | Tricarboxylate | Di- & tricarboxylates | *Salmonella enterica* | Proteobacteria | Bacteria |
| 2.A.85.1.1 | 2.A.85 | Q9FBT3 | SCO7159 | P75870 | 3.04E-015 | 8 | 12 | 5.5 | Aromatic acid | Aromatic compounds | *Escherichia coli* | Proteobacteria | Bacteria |
| 2.A.85.1.1 | 2.A.85 | Q93JH7 | SCO1985 | P75870 | 1.71E-010 | 11 | 12 | 5.45 | Aromatic acid | Aromatic compounds | *Escherichia coli* | Proteobacteria | Bacteria |
| 2.A.85.1.3 | 2.A.85 | Q9K3P9 | SCO1099 | P45537 | 0.000465927 | 10 | 9 | 4.5 | Aromatic acid | Aromatic compounds | *Escherichia coli* | Proteobacteria | Bacteria |
| 2.A.85.4.1 | 2.A.85 | Q9RD81 | SCO0796 | D4YEF0 | 4.91E-006 | 4 | 5 | 2.75 | Lantibiotic | Specific Drugs | *Aerococcus viridans* | Firmicutes | Bacteria |
| 2.A.86.1.7 | RND | Q9FBP6 | SCO5030 | O53656 | 3.71E-061 | 8 | 8 | 7.15 | Heme | Aromatic compounds | *Mycobacterium tuberculosis* | Actino-bacteria | Bacteria |
| 2.A.86.1.7 | RND | Q9RJL9 | SCO0513 | O53656 | 1.16E-041 | 8 | 8 | 7.55 | Heme | Aromatic compounds | *Mycobacterium tuberculosis* | Actino-bacteria | Bacteria |
| 2.A.86.1.7 | RND | Q9F321 | SCO3797 | O53656 | 1.46E-031 | 8 | 8 | 7 | Heme | Aromatic compounds | *Mycobacterium tuberculosis* | Actino-bacteria | Bacteria |
| 2.A.86.1.8 | 2.A.86 | O50485 | SCO5767 | O32095 | 8.39E-020 | 8 | 8 | 7 | Aldolose | Sugars & polyols | *Bacillus subtilis* | Firmicutes | Bacteria |
| 2.A.88.2.1 | 2.A.88 | Q9L203 | SCO2630 | Q2KUS5 | 7.25E-014 | 6 | 5 | 4.95 | Biotin | Vitamins & vitamin or cofactor precursors | *Bordetella avium* | Proteobacteria | Bacteria |
| 2.A.89.3.1 | 2.A.89 | Q9S2Y8 | SCO2027 | B2TCH9 | 1.62E-035 | 5 | 5 | 4.6 | Fe^2+^/Mn^2+^ | Cations | *Burkholderia phytofirmans* | Proteobacteria | Bacteria |
| 2.A.93.1.4 | 2.A.93 | Q9ZBW2 | SCO6556 | P39836 | 7.01E-076 | 8 | 10 | 7.4 | Na^+^ | Cations | *Escherichia coli* | Proteobacteria | Bacteria |
| 2.A.95.1.4 | 2.A.95 | Q9FBJ6 | SCO5162 | Q8J305 | 1.02E-022 | 6 | 6 | 4.05 | Glycine, alanine, L-amino acids | Amino acids & conjugates | *Thermococcus sp.* | Euryarchaeota | Archaea |
| 2.A.101.1.2 | U1T1 | Q9L0A3 | SCO2443 | C6B3K0 | 5.82E-120 | 13 | 11 | 8.65 | Dicarboxylate | Di- & tricarboxylates | *Rhizobium leguminosarum* | Proteobacteria | Bacteria |
| 2.A.102.3.1 | TSUP | Q9L2D4 | SCO2485 | P0AD30 | 1.67E-022 | 8 | 9 | 5.2 | 4-Toluene Sulfonate | Organoanions (noncarboxylic) | *Escherichia coli* | Proteobacteria | Bacteria |
| 2.A.102.3.1 | TSUP | Q9ADJ7 | SCO7462 | P0AD30 | 6.08E-013 | 8 | 9 | 5.3 | 4-Toluene Sulfonate | Organoanions (noncarboxylic) | *Escherichia coli* | Proteobacteria | Bacteria |
| 2.A.102.4.2 | TSUP | Q9RD63 | SCO0619 | E7BBJ3 | 5.61E-010 | 9 | 8 | 5.9 | Organo-sulfur-containing | Organoanions (noncarboxylic) | *Serratia sp.* | Proteobacteria | Bacteria |
| 2.A.103.1.3 | MPE | Q9L1H0 | SCO2607 | P07373 | 1.40E-052 | 10 | 10 | 6.45 | NAG-NAM-pentapeptide pyrophosphoryl undecaprenol (lipid II) | Lipids | *Bacillus subtilis* | Firmicutes | Bacteria |
| 2.A.103.1.3 | MPE | Q9ZBA6 | SCO2085 | P07373 | 6.57E-047 | 10 | 10 | 7.6 | NAG-NAM-pentapeptide pyrophosphoryl undecaprenol (lipid II) | Lipids | *Bacillus subtilis* | Firmicutes | Bacteria |
| 2.A.103.1.3 | MPE | Q9XA18 | SCO3846 | P07373 | 5.52E-038 | 12 | 10 | 5.15 | NAG-NAM-pentapeptide pyrophosphoryl undecaprenol (lipid II) | Lipids | *Bacillus subtilis* | Firmicutes | Bacteria |
| 2.A.103.1.3 | MPE | Q9XAF1 | SCO5302 | P07373 | 3.40E-033 | 12 | 10 | 6.8 | NAG-NAM-pentapeptide pyrophosphoryl undecaprenol (lipid II) | Lipids | *Bacillus subtilis* | Firmicutes | Bacteria |
| 3.A.1.1.1 | ABC | Q93J94 | SCO7503 | P0AEX9 | 3.25E-007 | 1 | 1 | 0.8 | Malatose (maltooligosaccharides) | Sugars & polyols | *Escherichia coli* | Proteobacteria | Bacteria |
| 3.A.1.1.3 | ABC | Q9RIV4 | SCO0952 | P0AG80 | 1.48E-014 | 1 | 1 | 0.4 | Glycerol-phosphate | Sugars & polyols | *Escherichia coli* | Proteobacteria | Bacteria |
| 3.A.1.1.5 | ABC | Q9X9X4 | SCO1898 | O30831 | 4.27E-093 | 1 | 1 | 0.5 | Hexitol (glucitol, mannitol) | Sugars & polyols | *Rhodobacter sphaeroides* | Proteobacteria | Bacteria |
| 3.A.1.1.5 | ABC | Q9X9X3 | SCO1899 | O30832 | 2.60E-049 | 6 | 6 | 6.4 | Hexitol (glucitol, mannitol) | Sugars & polyols | *Rhodobacter sphaeroides* | Proteobacteria | Bacteria |
| 3.A.1.1.5 | ABC | Q9X9X2 | SCO1900 | O30833 | 1.87E-048 | 6 | 6 | 6.5 | Hexitol (glucitol, mannitol) | Sugars & polyols | *Rhodobacter sphaeroides* | Proteobacteria | Bacteria |
| 3.A.1.1.6 | ABC | Q9KZ08 | SCO2229 | Q48397 | 8.94E-047 | 6 | 6 | 5.35 | Cyclodextrin | Sugars & polyols | *Klebsiella oxytoca* | Proteobacteria | Bacteria |
| 3.A.1.1.6 | ABC | Q9L1L6 | SCO5428 | Q48397 | 4.61E-029 | 6 | 6 | 5.9 | Cyclodextrin | Sugars & polyols | *Klebsiella oxytoca* | Proteobacteria | Bacteria |
| 3.A.1.1.6 | ABC | Q9RKF5 | SCO3482 | Q48397 | 1.07E-025 | 6 | 6 | 5.6 | Cyclodextrin | Sugars & polyols | *Klebsiella oxytoca* | Proteobacteria | Bacteria |
| 3.A.1.1.7 | ABC | Q93RU4 | SCO1882 | O51923 | 5.13E-025 | 1 | 1 | 0.9 | Maltose/trehalose | Sugars & polyols | *Thermococcus litoralis* | Euryarchaeota | Archaea |
| 3.A.1.1.7 | ABC | Q9L1L4 | SCO5430 | O51923 | 3.09E-024 | 1 | 1 | 0.65 | Maltose/trehalose | Sugars & polyols | *Thermococcus litoralis* | Euryarchaeota | Archaea |
| 3.A.1.1.7 | ABC | Q8CJY9 | SCO2658 | O51923 | 3.06E-019 | 1 | 1 | 0.9 | Maltose/trehalose | Sugars & polyols | *Thermococcus litoralis* | Euryarchaeota | Archaea |
| 3.A.1.1.7 | ABC | O87856 | SCO6601 | O51923 | 6.68E-016 | 1 | 1 | 0.9 | Maltose/trehalose | Sugars & polyols | *Thermococcus litoralis* | Euryarchaeota | Archaea |
| 3.A.1.1.10 | ABC | Q9KYS8 | SCO5687 | Q9KWT8 | 2.81E-051 | 6 | 6 | 2.35 | Alginate oligosacchardes | Carbohydrates | *Sphingomonassp* | Proteobacteria | Bacteria |
| 3.A.1.1.11 | ABC | Q9L271 | SCO1538 | Q93KC0 | 3.72E-065 | 6 | 6 | 6.4 | Sat/unsat oligogalacturonide | Carbohydrates | *Erwinia chrysanthemi* | Proteobacteria | Bacteria |
| 3.A.1.1.11 | ABC | Q9L272 | SCO1537 | Q93KB9 | 1.26E-054 | 6 | 7 | 5.5 | Sat/unsat oligogalacturonide | Carbohydrates | *Erwinia chrysanthemi* | Proteobacteria | Bacteria |
| 3.A.1.1.11 | ABC | Q9X860 | SCO3504 | Q93KC0 | 5.41E-048 | 6 | 6 | 6.55 | Sat/unsat oligogalacturonide | Carbohydrates | *Erwinia chrysanthemi* | Proteobacteria | Bacteria |
| 3.A.1.1.11 | ABC | Q9RK71 | SCO0539 | Q93KC0 | 1.37E-040 | 6 | 6 | 6.7 | Sat/unsat oligogalacturonide | Carbohydrates | *Erwinia chrysanthemi* | Proteobacteria | Bacteria |
| 3.A.1.1.11 | ABC | Q9L262 | SCO2659 | Q93KC0 | 2.08E-037 | 6 | 6 | 5.5 | Sat/unsat oligogalacturonide | Carbohydrates | *Erwinia chrysanthemi* | Proteobacteria | Bacteria |
| 3.A.1.1.11 | ABC | Q9L261 | SCO2660 | Q93KB9 | 2.44E-033 | 6 | 7 | 5.2 | Sat/unsat oligogalacturonide | Carbohydrates | *Erwinia chrysanthemi* | Proteobacteria | Bacteria |
| 3.A.1.1.12 | ABC | Q93JA9 | SCO7487 | Q9AI66 | 2.13E-034 | 7 | 6 | 4.25 | Palantinose | Sugars & polyols | *Erwinia rhapontici* | Proteobacteria | Bacteria |
| 3.A.1.1.17 | ABC | Q8CJL3 | SCO7012 | Q9R9Q6 | 3.32E-033 | 6 | 8 | 2.95 | Trehalose/maltose/sucrose | Sugars & polyols | *Rhizobium meliloti* | Proteobacteria | Bacteria |
| 3.A.1.1.17 | ABC | Q9L1L5 | SCO5429 | Q9R9Q6 | 2.70E-025 | 6 | 8 | 3.25 | Trehalose/maltose/sucrose | Sugars & polyols | *Rhizobium meliloti* | Proteobacteria | Bacteria |
| 3.A.1.1.18 | ABC | O50501 | SCO6007 | Q8RJU8 | 1.32E-087 | 6 | 6 | 3.2 | N-acetylglucosamine/N,N'-diacetyl chitobiose | Monocarboxylates | *Streptomyces olivaceoviridis* | Actino-bacteria | Bacteria |
| 3.A.1.1.18 | ABC | O50500 | SCO6006 | Q8RJU9 | 1.10E-059 | 6 | 6 | 5.9 | N-acetylglucosamine/N,N'-diacetyl chitobiose | Monocarboxylates | *Streptomyces olivaceoviridis* | Actino-bacteria | Bacteria |
| 3.A.1.1.18 | ABC | Q8CK62 | SCO0292 | Q8RJU8 | 4.31E-035 | 6 | 6 | 4.6 | N-acetylglucosamine/N,N'-diacetyl chitobiose | Monocarboxylates | *Streptomyces olivaceoviridis* | Actino-bacteria | Bacteria |
| 3.A.1.1.18 | ABC | Q9L025 | SCO7030 | Q8RJU8 | 8.62E-035 | 6 | 6 | 5.85 | N-acetylglucosamine/N,N'-diacetyl chitobiose | Monocarboxylates | *Streptomyces olivaceoviridis* | Actino-bacteria | Bacteria |
| 3.A.1.1.18 | ABC | Q9RJV6 | SCO0354 | Q8RJU8 | 8.92E-035 | 6 | 6 | 4.95 | N-acetylglucosamine/N,N'-diacetyl chitobiose | Monocarboxylates | *Streptomyces olivaceoviridis* | Actino-bacteria | Bacteria |
| 3.A.1.1.18 | ABC | O86594 | SCO6229 | Q8RJU8 | 3.15E-032 | 6 | 6 | 5.65 | N-acetylglucosamine/N,N'-diacetyl chitobiose | Monocarboxylates | *Streptomyces olivaceoviridis* | Actino-bacteria | Bacteria |
| 3.A.1.1.18 | ABC | Q7AKD8 | SCO7011 | Q8RJU8 | 2.39E-031 | 6 | 6 | 5.05 | N-acetylglucosamine/N,N'-diacetyl chitobiose | Monocarboxylates | *Streptomyces olivaceoviridis* | Actino-bacteria | Bacteria |
| 3.A.1.1.18 | ABC | Q9RL32 | SCO0455 | Q8RJU8 | 4.21E-031 | 6 | 6 | 5.65 | N-acetylglucosamine/N,N'-diacetyl chitobiose | Monocarboxylates | *Streptomyces olivaceoviridis* | Actino-bacteria | Bacteria |
| 3.A.1.1.18 | ABC | Q9RL33 | SCO0454 | Q8RJU9 | 2.19E-028 | 6 | 6 | 4.25 | N-acetylglucosamine/N,N'-diacetyl chitobiose | Monocarboxylates | *Streptomyces olivaceoviridis* | Actino-bacteria | Bacteria |
| 3.A.1.1.19 | ABC | Q9ADH7 | SCO6086 | Q8L125 | 2.30E-030 | 6 | 6 | 3.75 | Platinose | Sugars & polyols | *Agrobacterium tumefaciens* | Proteobacteria | Bacteria |
| 3.A.1.1.19 | ABC | Q93JA8 | SCO7488 | Q8L125 | 5.87E-029 | 6 | 6 | 3.6 | Platinose | Sugars & polyols | *Agrobacterium tumefaciens* | Proteobacteria | Bacteria |
| 3.A.1.1.19 | ABC | Q9L149 | SCO7410 | Q8L125 | 1.04E-027 | 6 | 6 | 3 | Platinose | Sugars & polyols | *Agrobacterium tumefaciens* | Proteobacteria | Bacteria |
| 3.A.1.1.20 | ABC | Q9L0B3 | SCO2433 | Q7WWQ9 | 1.73E-041 | 6 | 6 | 5.45 | Fructooligosaccharide | Carbohydrates | *Lactobacillus acidophilus* | Firmicutes | Bacteria |
| 3.A.1.1.20 | ABC | Q9RIV5 | SCO0951 | Q7WWQ9 | 2.10E-041 | 6 | 6 | 5.85 | Fructooligosaccharide | Carbohydrates | *Lactobacillus acidophilus* | Firmicutes | Bacteria |
| 3.A.1.1.20 | ABC | Q93J93 | SCO7504 | Q7WWQ9 | 4.42E-038 | 6 | 6 | 6.35 | Fructooligosaccharide | Carbohydrates | *Lactobacillus acidophilus* | Firmicutes | Bacteria |
| 3.A.1.1.20 | ABC | Q9RIV6 | SCO0950 | Q7WWQ8 | 6.15E-035 | 6 | 6 | 5.7 | Fructooligosaccharide | Carbohydrates | *Lactobacillus acidophilus* | Firmicutes | Bacteria |
| 3.A.1.1.20 | ABC | Q9RJV7 | SCO0353 | Q7WWQ9 | 5.39E-034 | 6 | 6 | 6.4 | Fructooligosaccharide | Carbohydrates | *Lactobacillus acidophilus* | Firmicutes | Bacteria |
| 3.A.1.1.20 | ABC | Q9L150 | SCO7409 | Q7WWQ8 | 1.48E-033 | 6 | 6 | 4.85 | Fructooligosaccharide | Carbohydrates | *Lactobacillus acidophilus* | Firmicutes | Bacteria |
| 3.A.1.1.20 | ABC | Q9FBS6 | SCO7166 | Q7WWQ9 | 2.72E-031 | 6 | 6 | 5.8 | Fructooligosaccharide | Carbohydrates | *Lactobacillus acidophilus* | Firmicutes | Bacteria |
| 3.A.1.1.20 | ABC | Q9RJA3 | SCO0661 | Q7WWQ9 | 7.58E-024 | 6 | 6 | 6.05 | Fructooligosaccharide | Carbohydrates | *Lactobacillus acidophilus* | Firmicutes | Bacteria |
| 3.A.1.1.20 | ABC | Q9K443 | SCO1056 | Q7WWR0 | 1.02E-021 | 1 | 1 | 0.45 | Fructooligosaccharide | Carbohydrates | *Lactobacillus acidophilus* | Firmicutes | Bacteria |
| 3.A.1.1.20 | ABC | Q9RK72 | SCO0538 | Q7WWR0 | 3.96E-020 | 1 | 1 | 0.9 | Fructooligosaccharide | Carbohydrates | *Lactobacillus acidophilus* | Firmicutes | Bacteria |
| 3.A.1.1.21 | ABC | Q9L027 | SCO7028 | Q9L027 | 0 | 1 | 1 | 0.9 | Xylobiose | Sugars & polyols | *Streptomyces coelicolor* | Actino-bacteria | Bacteria |
| 3.A.1.1.21 | ABC | Q9L026 | SCO7029 | Q9L026 | 0 | 6 | 6 | 6.75 | Xylobiose | Sugars & polyols | *Streptomyces coelicolor* | Actino-bacteria | Bacteria |
| 3.A.1.1.21 | ABC | Q9S2C3 | SCO0290 | Q9L027 | 6.59E-073 | 1 | 1 | 0.9 | Xylobiose | Sugars & polyols | *Streptomyces coelicolor* | Actino-bacteria | Bacteria |
| 3.A.1.1.21 | ABC | Q9S2C2 | SCO0291 | Q9L026 | 1.04E-064 | 6 | 6 | 5.8 | Xylobiose | Sugars & polyols | *Streptomyces coelicolor* | Actino-bacteria | Bacteria |
| 3.A.1.1.21 | ABC | Q9FBS5 | SCO7167 | Q9L027 | 1.69E-017 | 1 | 1 | 0.8 | Xylobiose | Sugars & polyols | *Streptomyces coelicolor* | Actino-bacteria | Bacteria |
| 3.A.1.1.21 | ABC | Q9KXV5 | SCO4286 | Q9L027 | 1.65E-013 | 1 | 1 | 0.8 | Xylobiose | Sugars & polyols | *Streptomyces coelicolor* | Actino-bacteria | Bacteria |
| 3.A.1.1.21 | ABC | Q9RJV8 | SCO0352 | Q9L027 | 1.27E-010 | 1 | 1 | 0.7 | Xylobiose | Sugars & polyols | *Streptomyces coelicolor* | Actino-bacteria | Bacteria |
| 3.A.1.1.21 | ABC | Q9S1S7 | SCO0065 | Q9L027 | 6.90E-010 | 1 | 1 | 0.9 | Xylobiose | Sugars & polyols | *Streptomyces coelicolor* | Actino-bacteria | Bacteria |
| 3.A.1.1.22 | ABC | Q9KZ07 | SCO2230 | Q9X0T0 | 6.28E-038 | 6 | 8 | 4.4 | Maltose | Sugars & polyols | *Thermotoga maritima* | Thermotogae | Bacteria |
| 3.A.1.1.23 | ABC | Q9F3I6 | SCO2795 | Q9X9R7 | 2.59E-114 | 1 | 1 | 0.9 | Cellobiose/cellotriose | Sugars & polyols | *Streptomyces reticuli* | Actino-bacteria | Bacteria |
| 3.A.1.1.23 | ABC | Q9F3I5 | SCO2796 | Q9X9R6 | 1.99E-109 | 6 | 6 | 5.75 | Cellobiose/cellotriose | Sugars & polyols | *Streptomyces reticuli* | Actino-bacteria | Bacteria |
| 3.A.1.1.23 | ABC | Q9F3I4 | SCO2797 | Q9X9R5 | 2.85E-102 | 6 | 6 | 5.6 | Cellobiose/cellotriose | Sugars & polyols | *Streptomyces reticuli* | Actino-bacteria | Bacteria |
| 3.A.1.1.23 | ABC | Q9F3C0 | SCO7555 | Q9X9R7 | 5.58E-099 | 1 | 1 | 0.6 | Cellobiose/cellotriose | Sugars & polyols | *Streptomyces reticuli* | Actino-bacteria | Bacteria |
| 3.A.1.1.23 | ABC | Q9F3B9 | SCO7556 | Q9X9R6 | 1.66E-094 | 6 | 6 | 4.85 | Cellobiose/cellotriose | Sugars & polyols | *Streptomyces reticuli* | Actino-bacteria | Bacteria |
| 3.A.1.1.23 | ABC | Q9F3B8 | SCO7557 | Q9X9R5 | 5.68E-094 | 6 | 6 | 5.85 | Cellobiose/cellotriose | Sugars & polyols | *Streptomyces reticuli* | Actino-bacteria | Bacteria |
| 3.A.1.1.23 | ABC | Q9K436 | SCO1063 | Q9X9R5 | 5.83E-042 | 6 | 6 | 5.6 | Cellobiose/cellotriose | Sugars & polyols | *Streptomyces reticuli* | Actino-bacteria | Bacteria |
| 3.A.1.1.23 | ABC | Q9K435 | SCO1064 | Q9X9R6 | 2.37E-038 | 6 | 6 | 6.1 | Cellobiose/cellotriose | Sugars & polyols | *Streptomyces reticuli* | Actino-bacteria | Bacteria |
| 3.A.1.1.23 | ABC | Q9RK90 | SCO0272 | Q9X9R5 | 1.83E-030 | 6 | 6 | 3.7 | Cellobiose/cellotriose | Sugars & polyols | *Streptomyces reticuli* | Actino-bacteria | Bacteria |
| 3.A.1.1.24 | ABC | Q9S1S5 | SCO0067 | Q72KX4 | 2.82E-027 | 6 | 6 | 4.8 | Glucose/mannose | Sugars & polyols | *Thermus thermophilus* | Chordata | Eukarya |
| 3.A.1.1.25 | ABC | O87858 | SCO6603 | Q72H66 | 8.28E-045 | 6 | 6 | 5.35 | Trehalose/maltose/sucrose/palatinose | Sugars & polyols | *Thermus thermophilus* | Chordata | Eukarya |
| 3.A.1.1.25 | ABC | Q9X9Y5 | SCO1887 | Q72H67 | 5.14E-041 | 6 | 6 | 5.15 | Trehalose/maltose/sucrose/palatinose | Sugars & polyols | *Thermus thermophilus* | Chordata | Eukarya |
| 3.A.1.1.25 | ABC | Q9K442 | SCO1057 | Q72H67 | 1.13E-040 | 6 | 6 | 5 | Trehalose/maltose/sucrose/palatinose | Sugars & polyols | *Thermus thermophilus* | Chordata | Eukarya |
| 3.A.1.1.25 | ABC | Q9RK91 | SCO0271 | Q72H67 | 2.30E-038 | 6 | 6 | 5.25 | Trehalose/maltose/sucrose/palatinose | Sugars & polyols | *Thermus thermophilus* | Chordata | Eukarya |
| 3.A.1.1.25 | ABC | Q9RKF4 | SCO3483 | Q72H67 | 2.18E-035 | 6 | 6 | 5.6 | Trehalose/maltose/sucrose/palatinose | Sugars & polyols | *Thermus thermophilus* | Chordata | Eukarya |
| 3.A.1.1.25 | ABC | O87857 | SCO6602 | Q72H67 | 9.81E-028 | 6 | 6 | 4.1 | Trehalose/maltose/sucrose/palatinose | Sugars & polyols | *Thermus thermophilus* | Chordata | Eukarya |
| 3.A.1.1.25 | ABC | Q9RKH7 | SCO3455 | Q72H67 | 2.51E-014 | 6 | 6 | 5.1 | Trehalose/maltose/sucrose/palatinose | Sugars & polyols | *Thermus thermophilus* | Chordata | Eukarya |
| 3.A.1.1.28 | ABC | Q9FBS7 | SCO7165 | Q00751 | 2.12E-033 | 7 | 6 | 5.6 | Raffinose/stachyose | Sugars & polyols | *Streptococcus mutans* | Actino-bacteria | Bacteria |
| 3.A.1.1.28 | ABC | O86595 | SCO6230 | Q00750 | 4.29E-029 | 6 | 6 | 5.8 | Raffinose/stachyose | Sugars & polyols | *Streptococcus mutans* | Actino-bacteria | Bacteria |
| 3.A.1.1.28 | ABC | O86596 | SCO6231 | Q00749 | 1.26E-011 | 1 | 1 | 0.55 | Raffinose/stachyose | Sugars & polyols | *Streptococcus mutans* | Actino-bacteria | Bacteria |
| 3.A.1.1.29 | ABC | Q9KYS7 | SCO5688 | A9QDR8 | 3.43E-038 | 6 | 6 | 5.4 | Aldouronate | Sugars & polyols | *Paenibacillus sp.* | Firmicutes | Bacteria |
| 3.A.1.1.32 | ABC | Q9L055 | SCO2980 | Q55473 | 2.49E-062 | 6 | 6 | 5.45 | Glucosylglycerol | Sugars & polyols | *Synechocystis sp.* | Cyanobacteria | Bacteria |
| 3.A.1.1.32 | ABC | Q9L057 | SCO2978 | Q55471 | 1.48E-058 | 1 | 1 | 0.8 | Glucosylglycerol | Sugars & polyols | *Synechocystis sp.* | Cyanobacteria | Bacteria |
| 3.A.1.1.32 | ABC | Q9S1S6 | SCO0066 | Q55472 | 4.16E-032 | 6 | 7 | 5.35 | Glucosylglycerol | Sugars & polyols | *Synechocystis sp.* | Cyanobacteria | Bacteria |
| 3.A.1.1.32 | ABC | Q9L0Z4 | SCO1465 | Q55471 | 1.41E-028 | 1 | 1 | 0.85 | Glucosylglycerol | Sugars & polyols | *Synechocystis sp.* | Cyanobacteria | Bacteria |
| 3.A.1.1.32 | ABC | Q9L056 | SCO2979 | Q55472 | 2.68E-026 | 6 | 7 | 3.15 | Glucosylglycerol | Sugars & polyols | *Synechocystis sp.* | Cyanobacteria | Bacteria |
| 3.A.1.1.33 | ABC | Q9K491 | SCO5232 | Q8KN19 | 0 | 1 | 1 | 0.85 | N,N'-diacetylchitobiose | Sugars & polyols | *Streptomyces sp.* | Actino-bacteria | Bacteria |
| 3.A.1.1.33 | ABC | Q9K490 | SCO5233 | Q8KN18 | 4.17E-154 | 6 | 6 | 6 | N,N'-diacetylchitobiose | Sugars & polyols | *Streptomyces sp.* | Actino-bacteria | Bacteria |
| 3.A.1.1.33 | ABC | Q9K489 | SCO5234 | Q8KN17 | 5.44E-140 | 6 | 6 | 6.15 | N,N'-diacetylchitobiose | Sugars & polyols | *Streptomyces sp.* | Actino-bacteria | Bacteria |
| 3.A.1.1.33 | ABC | Q9RK77 | SCO0533 | Q8KN17 | 4.83E-084 | 6 | 6 | 5.8 | N,N'-diacetylchitobiose | Sugars & polyols | *Streptomyces sp.* | Actino-bacteria | Bacteria |
| 3.A.1.1.33 | ABC | Q9RK78 | SCO0532 | Q8KN18 | 1.11E-063 | 6 | 6 | 6.25 | N,N'-diacetylchitobiose | Sugars & polyols | *Streptomyces sp.* | Actino-bacteria | Bacteria |
| 3.A.1.1.33 | ABC | Q9RCZ6 | SCO0914 | Q8KN19 | 3.06E-063 | 1 | 1 | 0.85 | N,N'-diacetylchitobiose | Sugars & polyols | *Streptomyces sp.* | Actino-bacteria | Bacteria |
| 3.A.1.1.33 | ABC | Q9L1V0 | SCO2944 | Q8KN17 | 3.00E-047 | 6 | 6 | 6 | N,N'-diacetylchitobiose | Sugars & polyols | *Streptomyces sp.* | Actino-bacteria | Bacteria |
| 3.A.1.1.33 | ABC | Q9L1U9 | SCO2945 | Q8KN18 | 3.13E-045 | 6 | 6 | 6.1 | N,N'-diacetylchitobiose | Sugars & polyols | *Streptomyces sp.* | Actino-bacteria | Bacteria |
| 3.A.1.1.33 | ABC | Q9X9Y6 | SCO1886 | Q8KN17 | 1.79E-041 | 6 | 6 | 4.6 | N,N'-diacetylchitobiose | Sugars & polyols | *Streptomyces sp.* | Actino-bacteria | Bacteria |
| 3.A.1.1.33 | ABC | Q9ADH6 | SCO6087 | Q8KN17 | 4.32E-040 | 6 | 6 | 6.05 | N,N'-diacetylchitobiose | Sugars & polyols | *Streptomyces sp.* | Actino-bacteria | Bacteria |
| 3.A.1.1.33 | ABC | Q9K441 | SCO1058 | Q8KN17 | 1.56E-035 | 6 | 6 | 4.95 | N,N'-diacetylchitobiose | Sugars & polyols | *Streptomyces sp.* | Actino-bacteria | Bacteria |
| 3.A.1.1.34 | ABC | Q9RK70 | SCO0540 | P94530 | 1.89E-043 | 6 | 6 | 5.3 | L-arabinose | Sugars & polyols | *Bacillus subtilis* | Firmicutes | Bacteria |
| 3.A.1.1.34 | ABC | Q9L0B4 | SCO2432 | P94530 | 1.96E-043 | 6 | 6 | 6.05 | L-arabinose | Sugars & polyols | *Bacillus subtilis* | Firmicutes | Bacteria |
| 3.A.1.1.34 | ABC | Q9X859 | SCO3503 | P94530 | 3.60E-043 | 6 | 6 | 3.65 | L-arabinose | Sugars & polyols | *Bacillus subtilis* | Firmicutes | Bacteria |
| 3.A.1.1.34 | ABC | Q93J92 | SCO7505 | P94530 | 2.14E-042 | 6 | 6 | 5.15 | L-arabinose | Sugars & polyols | *Bacillus subtilis* | Firmicutes | Bacteria |
| 3.A.1.1.34 | ABC | Q9RJA2 | SCO0662 | P94530 | 9.34E-031 | 6 | 6 | 5.15 | L-arabinose | Sugars & polyols | *Bacillus subtilis* | Firmicutes | Bacteria |
| 3.A.1.1.34 | ABC | Q9K434 | SCO1065 | P94528 | 2.64E-025 | 1 | 2 | 0.4 | Arabinose | Sugars & polyols | *Bacillus subtilis* | Firmicutes | Bacteria |
| 3.A.1.1.34 | ABC | Q9RJA4 | SCO0660 | P94528 | 1.89E-011 | 1 | 2 | 0.85 | Arabinose | Sugars & polyols | *Bacillus subtilis* | Firmicutes | Bacteria |
| 3.A.1.1.34 | ABC | Q8CK63 | SCO0273 | P94528 | 8.79E-010 | 1 | 2 | 0.9 | Arabinose | Sugars & polyols | *Bacillus subtilis* | Firmicutes | Bacteria |
| 3.A.1.1.34 | ABC | Q9L0B2 | SCO2434 | P94528 | 9.24E-010 | 1 | 2 | 0.95 | Arabinose | Sugars & polyols | *Bacillus subtilis* | Firmicutes | Bacteria |
| 3.A.1.1.34 | ABC | Q9RKF3 | SCO3484 | P94528 | 1.25E-009 | 1 | 2 | 0.85 | Arabinose | Sugars & polyols | *Bacillus subtilis* | Firmicutes | Bacteria |
| 3.A.1.2.1 | ABC | Q9RDI0 | SCO2747 | P02925 | 6.52E-068 | 9 | 1 | 1 | Ribose | Sugars & polyols | *Escherichia coli* | Proteobacteria | Bacteria |
| 3.A.1.2.4 | ABC | O50505 | SCO6011 | P0AGI4 | 3.32E-040 | 12 | 10 | 8.3 | Xylose | Sugars & polyols | *Escherichia coli* | Proteobacteria | Bacteria |
| 3.A.1.2.5 | ABC | Q9RDN3 | SCO2404 | P25548 | 1.87E-115 | 1 | 1 | 0.2 | Multiple sugars | Sugars & polyols | *Agrobacterium tumefaciens* | Proteobacteria | Bacteria |
| 3.A.1.2.5 | ABC | Q9RDN1 | SCO2406 | O05177 | 1.82E-098 | 12 | 12 | 9.8 | Multiple sugars | Sugars & polyols | *Agrobacterium tumefaciens* | Proteobacteria | Bacteria |
| 3.A.1.2.8 | ABC | Q9RD68 | SCO0809 | P0AFS1 | 3.56E-029 | 10 | 10 | 8.9 | Unknown (autoinducer) | Unknown | *Escherichia coli* | Proteobacteria | Bacteria |
| 3.A.1.2.9 | ABC | Q9RD67 | SCO0810 | Q7BSH3 | 1.42E-039 | 10 | 8 | 7.1 | Rhamnose | Sugars & polyols | *Rhizobium leguminosarum* | Proteobacteria | Bacteria |
| 3.A.1.2.12 | ABC | Q9AK38 | SCO4888 | Q8DU39 | 7.63E-037 | 12 | 9 | 6.65 | Deoxy- and ribonucleosides | Nucleic acids | *Streptococcus mutans* | Firmicutes | Bacteria |
| 3.A.1.2.15 | ABC | O69943 | SCO6568 | A8H4W6 | 1.57E-047 | 6 | 9 | 4.8 | Xylitol | Sugars & polyols | *Shewanella pealeana* | Proteobacteria | Bacteria |
| 3.A.1.2.16 | ABC | Q9KZH2 | SCO6980 | Q1M4Q8 | 2.11E-024 | 8 | 10 | 7 | Erythritol | Sugars & polyols | *Rhizobium leguminosarum* | Proteobacteria | Bacteria |
| 3.A.1.2.16 | ABC | Q9RKT4 | SCO6258 | Q1M4Q8 | 3.21E-024 | 10 | 10 | 8.85 | Erythritol | Sugars & polyols | *Rhizobium leguminosarum* | Proteobacteria | Bacteria |
| 3.A.1.2.17 | ABC | Q9AK39 | SCO4887 | A2RKA6 | 7.99E-029 | 10 | 8 | 7.1 | Purine/cytidine | Amino acids & conjugates | *Lactococcus lactis* | Firmicutes | Bacteria |
| 3.A.1.3.9 | ABC | O50493 | SCO5775 | P48244 | 2.71E-065 | 5 | 5 | 5.55 | Glutamate | Amino acids & conjugates | *Corynebacterium glutamicum* | Actino-bacteria | Bacteria |
| 3.A.1.3.9 | ABC | O50494 | SCO5776 | P48242 | 4.28E-059 | 1 | 1 | 0.85 | Glutamate | Amino acids & conjugates | *Corynebacterium glutamicum* | Actino-bacteria | Bacteria |
| 3.A.1.3.9 | ABC | O50492 | SCO5774 | P48245 | 2.96E-052 | 6 | 5 | 4.6 | Glutamate | Amino acids & conjugates | *Corynebacterium glutamicum* | Actino-bacteria | Bacteria |
| 3.A.1.3.12 | ABC | Q9F3K6 | SCO5259 | P73544 | 2.39E-034 | 6 | 6 | 3.7 | Arginine/lysine/histidine/glutamine | Amino acids & conjugates | *Synechocystis sp.* | Cyanobacteria | Bacteria |
| 3.A.1.3.14 | ABC | Q9RDC1 | SCO2830 | P42200 | 1.66E-027 | 5 | 5 | 5.3 | L-cystine | Amino acids & conjugates | *Bacillus subtilis* | Firmicutes | Bacteria |
| 3.A.1.3.15 | ABC | Q9RDC2 | SCO2829 | P54536 | 7.98E-030 | 5 | 5 | 3.5 | Arginine | Amino acids & conjugates | *Bacillus subtilis* | Firmicutes | Bacteria |
| 3.A.1.4.1 | ABC | Q9S2J4 | SCO2009 | P0AEX7 | 1.37E-052 | 9 | 9 | 7.7 | Leucine/isoleucine/valine | Amino acids & conjugates | *Escherichia coli* | Proteobacteria | Bacteria |
| 3.A.1.4.1 | ABC | Q9S2J3 | SCO2010 | P22729 | 1.54E-044 | 14 | 10 | 6.3 | Leucine/isoleucine/valine | Amino acids & conjugates | *Escherichia coli* | Proteobacteria | Bacteria |
| 3.A.1.4.1 | ABC | Q9FBZ9 | SCO7183 | P0AEX7 | 1.73E-030 | 7 | 9 | 5 | Leucine/isoleucine/valine | Amino acids & conjugates | *Escherichia coli* | Proteobacteria | Bacteria |
| 3.A.1.4.1 | ABC | Q9FBZ8 | SCO7184 | P22729 | 3.53E-020 | 12 | 10 | 5.8 | Leucine/isoleucine/valine | Amino acids & conjugates | *Escherichia coli* | Proteobacteria | Bacteria |
| 3.A.1.4.1 | ABC | Q9L2K1 | SCO0707 | P22729 | 8.35E-019 | 10 | 10 | 5.95 | Leucine/isoleucine/valine | Amino acids & conjugates | *Escherichia coli* | Proteobacteria | Bacteria |
| 3.A.1.4.3 | ABC | Q9S2J5 | SCO2008 | Q9L3M3 | 5.11E-032 | 1 | 1 | 0.05 | Acidic, basic, polar, semipolar and hydrophobic amino acids | Amino acids & conjugates | *Rhizobium leguminosarum* | Proteobacteria | Bacteria |
| 3.A.1.4.3 | ABC | Q9L2K0 | SCO0708 | Q8VM85 | 2.46E-024 | 8 | 7 | 6.05 | Acidic, basic, polar, semipolar and hydrophobic amino acids | Amino acids & conjugates | *Rhizobium leguminosarum* | Proteobacteria | Bacteria |
| 3.A.1.5.2 | ABC | Q9F353 | SCO5117 | P26906 | 4.24E-031 | 1 | 1 | 0.85 | Dipeptide (Heme and ALA as well) | Peptides | *Bacillus subtilis* | Firmicutes | Bacteria |
| 3.A.1.5.2 | ABC | O86636 | SCO5716 | P26906 | 8.94E-028 | 2 | 1 | 0.75 | Dipeptide (Heme and ALA as well) | Peptides | *Bacillus subtilis* | Firmicutes | Bacteria |
| 3.A.1.5.2 | ABC | Q9RJ53 | SCO1655 | P26906 | 5.22E-025 | 1 | 1 | 0.9 | Dipeptide (Heme and ALA as well) | Peptides | *Bacillus subtilis* | Firmicutes | Bacteria |
| 3.A.1.5.3 | ABC | Q9F3B1 | SCO7564 | P33591 | 1.77E-066 | 6 | 6 | 5.35 | Ni^2+^ | Cations | *Escherichia coli* | Proteobacteria | Bacteria |
| 3.A.1.5.3 | ABC | O86691 | SCO6645 | P33591 | 2.74E-023 | 11 | 6 | 5.05 | Ni^2+^ | Cations | *Escherichia coli* | Proteobacteria | Bacteria |
| 3.A.1.5.11 | ABC | O86573 | SCO5478 | P75798 | 4.33E-053 | 7 | 6 | 2.25 | Glutathione | Peptides | *Escherichia coli* | Proteobacteria | Bacteria |
| 3.A.1.5.11 | ABC | Q9F351 | SCO5119 | P75799 | 1.67E-048 | 6 | 6 | 5.9 | Glutathione | Peptides | *Escherichia coli* | Proteobacteria | Bacteria |
| 3.A.1.5.11 | ABC | Q93IU1 | SCO5114 | P75798 | 3.26E-045 | 6 | 6 | 3.6 | Glutathione | Peptides | *Escherichia coli* | Proteobacteria | Bacteria |
| 3.A.1.5.11 | ABC | Q9K3Y9 | SCO1344 | P75797 | 3.06E-021 | 1 | 1 | 0.9 | Glutathione | Peptides | *Escherichia coli* | Proteobacteria | Bacteria |
| 3.A.1.5.15 | ABC | Q8CJP2 | SCO6114 | Q9X268 | 2.31E-009 | 1 | 1 | 0.25 | Mannose/mannoside | Sugars & polyols | *Thermotoga maritima* | Thermotogae | Bacteria |
| 3.A.1.5.17 | ABC | Q9EWP9 | SCO7678 | Q92NF0 | 7.93E-037 | 6 | 6 | 5.4 | Proline/betaine | Amino acids & conjugates | *Rhizobium meliloti* | Proteobacteria | Bacteria |
| 3.A.1.5.17 | ABC | Q9EWP8 | SCO7679 | Q92NE9 | 2.77E-027 | 6 | 5 | 5.35 | Proline/betaine | Amino acids & conjugates | *Rhizobium meliloti* | Proteobacteria | Bacteria |
| 3.A.1.5.18 | ABC | O54107 | SCO5911 | O51307 | 2.26E-025 | 1 | 1 | 0.65 | Oligopeptide | Peptides | *Borrelia burgdorferi* | Spirochaetes | Bacteria |
| 3.A.1.5.20 | ABC | Q8CJP3 | SCO6113 | P42062 | 2.06E-062 | 6 | 6 | 5.55 | 5-6 amino acyl Oligopeptide | Peptides | *Bacillus subtilis* | Firmicutes | Bacteria |
| 3.A.1.5.20 | ABC | Q9F3B0 | SCO7565 | P42063 | 5.21E-062 | 5 | 6 | 4.95 | 5-6 amino acyl Oligopeptide | Peptides | *Bacillus subtilis* | Firmicutes | Bacteria |
| 3.A.1.5.20 | ABC | O86571 | SCO5476 | P42063 | 4.57E-060 | 6 | 6 | 2.8 | 5-6 amino acyl Oligopeptide | Peptides | *Bacillus subtilis* | Firmicutes | Bacteria |
| 3.A.1.5.20 | ABC | Q9ADF1 | SCO6112 | P42063 | 5.95E-056 | 6 | 6 | 5.8 | 5-6 amino acyl Oligopeptide | Peptides | *Bacillus subtilis* | Firmicutes | Bacteria |
| 3.A.1.5.20 | ABC | Q9ZBG2 | SCO6451 | P42061 | 1.08E-054 | 1 | 1 | 0.45 | 5-6 amino acyl Oligopeptide | Peptides | *Bacillus subtilis* | Firmicutes | Bacteria |
| 3.A.1.5.20 | ABC | Q9ZBG1 | SCO6452 | P42062 | 2.26E-054 | 7 | 6 | 6.1 | 5-6 amino acyl Oligopeptide | Peptides | *Bacillus subtilis* | Firmicutes | Bacteria |
| 3.A.1.5.20 | ABC | Q9ZBG0 | SCO6453 | P42063 | 2.92E-049 | 4 | 6 | 2.95 | 5-6 amino acyl Oligopeptide | Peptides | *Bacillus subtilis* | Firmicutes | Bacteria |
| 3.A.1.5.20 | ABC | Q8CJQ7 | SCO5715 | P42062 | 1.69E-047 | 6 | 6 | 5.6 | 5-6 amino acyl Oligopeptide | Peptides | *Bacillus subtilis* | Firmicutes | Bacteria |
| 3.A.1.5.20 | ABC | Q9Z533 | SCO5714 | P42063 | 2.29E-047 | 6 | 6 | 5.65 | 5-6 amino acyl Oligopeptide | Peptides | *Bacillus subtilis* | Firmicutes | Bacteria |
| 3.A.1.5.22 | ABC | Q93IU3 | SCO5112 | Q5V9S0 | 2.83E-046 | 6 | 6 | 3.1 | Peptide | Peptides | *Vibrio fluvialis* | Proteobacteria | Bacteria |
| 3.A.1.5.27 | ABC | Q9F352 | SCO5118 | P45096 | 1.94E-054 | 6 | 6 | 2.95 | Dipeptide - glutathione | Peptides | *Haemophilus influenzae* | Proteobacteria | Bacteria |
| 3.A.1.6.8 | ABC | O86752 | SCO5647 | Q73RE5 | 6.34E-029 | 12 | 12 | 9.5 | Thiamine pyrophosphate | Amino acids & conjugates | *Treponema denticola* | Spirochaetes | Bacteria |
| 3.A.1.7.1 | ABC | Q9L217 | SCO6815 | P0AGH8 | 3.88E-038 | 13 | 6 | 5.15 | Phosphate | Anions | *Escherichia coli* | Proteobacteria | Bacteria |
| 3.A.1.7.2 | ABC | Q9KZW0 | SCO4141 | Q7WTY7 | 1.04E-074 | 6 | 7 | 4.35 | Phosphate | Anions | *Mycobacterium smegmatis* | Actino-bacteria | Bacteria |
| 3.A.1.7.2 | ABC | Q9KZV9 | SCO4142 | Q7WTY8 | 5.34E-073 | 1 | 1 | 0.9 | Phosphate | Anions | *Mycobacterium smegmatis* | Actino-bacteria | Bacteria |
| 3.A.1.7.2 | ABC | Q9KZW1 | SCO4140 | Q7WTY6 | 2.10E-064 | 8 | 6 | 5.2 | Phosphate | Anions | *Mycobacterium smegmatis* | Actino-bacteria | Bacteria |
| 3.A.1.8.1 | ABC | Q9X8Y7 | SCO3705 | P0AF01 | 6.43E-040 | 6 | 5 | 4.35 | Molybdenum | Cations | *Escherichia coli* | Proteobacteria | Bacteria |
| 3.A.1.11.1 | ABC | O86829 | SCO5670 | P0AFK6 | 2.16E-039 | 6 | 6 | 6.15 | Spermidine/putrescine | Amines, amides, polyamines, & organocations | *Escherichia coli* | Proteobacteria | Bacteria |
| 3.A.1.11.1 | ABC | O86832 | SCO5667 | P0AFK9 | 3.21E-024 | 1 | 1 | 0.05 | Spermidine/putrescine | Amines, amides, polyamines, & organocations | *Escherichia coli* | Proteobacteria | Bacteria |
| 3.A.1.11.2 | ABC | O86830 | SCO5669 | P31135 | 3.45E-039 | 6 | 6 | 5.05 | Putrescine | Amines, amides, polyamines, & organocations | *Escherichia coli* | Proteobacteria | Bacteria |
| 3.A.1.11.3 | ABC | Q9RKH8 | SCO3454 | Q44381 | 4.96E-020 | 6 | 6 | 6.4 | Mannopine | Amines, amides, polyamines, & organocations | *Agrobacterium tumefaciens* | Proteobacteria | Bacteria |
| 3.A.1.12.3 | ABC | Q9R432 | SCO2932 | Q45461 | 1.21E-032 | 6 | 5 | 4.5 | Choline | Vitamins & vitamin or cofactor precursors | *Bacillus subtilis* | Firmicutes | Bacteria |
| 3.A.1.12.3 | ABC | Q9S2F1 | SCO2930 | Q45461 | 4.29E-028 | 5 | 5 | 4.15 | Choline | Vitamins & vitamin or cofactor precursors | *Bacillus subtilis* | Firmicutes | Bacteria |
| 3.A.1.12.3 | ABC | Q9X830 | SCO6063 | Q45461 | 2.46E-016 | 5 | 5 | 4.4 | Choline | Vitamins & vitamin or cofactor precursors | *Bacillus subtilis* | Firmicutes | Bacteria |
| 3.A.1.12.5 | ABC | Q9RJ81 | SCO1620 | Q9RQ05 | 2.90E-107 | 6 | 6 | 5.05 | Glycine-betaine and proline | Amino acids & conjugates | *Lactococcus lactis* | Firmicutes | Bacteria |
| 3.A.1.12.6 | ABC | Q9K3I9 | SCO4831 | Q9KKE2 | 1.62E-048 | 16 | 9 | 4.6 | Histidine, proline, proline-betaine, glycine betaine | Amino acids & conjugates | *Rhizobium meliloti* | Proteobacteria | Bacteria |
| 3.A.1.12.6 | ABC | Q9K3I8 | SCO4832 | Q9KKE3 | 5.82E-015 | 1 | 1 | 0.5 | Histidine, proline, proline-betaine, glycine betaine | Amino acids & conjugates | *Rhizobium meliloti* | Proteobacteria | Bacteria |
| 3.A.1.12.8 | ABC | Q9X831 | SCO6064 | Q93A34 | 7.79E-024 | 5 | 6 | 4.35 | Proline/glycine (oxgall + bile compounds) | Amino acids & conjugates | *Listeria monocytogenes* | Firmicutes | Bacteria |
| 3.A.1.14.12 | ABC | Q9L179 | SCO7398 | Q9L179 | 0 | 20 | 20 | 20.05 | Desferrioxamine | Amines, amides, polyamines, & organocations | *Streptomyces coelicolor* | Actino-bacteria | Bacteria |
| 3.A.1.14.13 | ABC | Q9RK12 | SCO0494 | Q9RK12 | 0 | 1 | 1 | 0.9 | Coelichelin | Siderophores; siderophores-Fe complexes | *Streptomyces coelicolor* | Actino-bacteria | Bacteria |
| 3.A.1.14.13 | ABC | Q9RK09 | SCO0497 | Q9RK09 | 0 | 9 | 9 | 9.7 | Coelichelin | Siderophores; siderophores-Fe complexes | *Streptomyces coelicolor* | Actino-bacteria | Bacteria |
| 3.A.1.14.13 | ABC | Q9RK10 | SCO0496 | Q9RK10 | 0 | 9 | 9 | 10 | Coelichelin | Siderophores; siderophores-Fe complexes | *Streptomyces coelicolor* | Actino-bacteria | Bacteria |
| 3.A.1.14.13 | ABC | Q9RK11 | SCO0495 | Q9RK11 | 2.04E-160 | 1 | 1 | 0.9 | Coelichelin | Siderophores; siderophores-Fe complexes | *Streptomyces coelicolor* | Actino-bacteria | Bacteria |
| 3.A.1.14.19 | ABC | Q9RKQ5 | SCO2273 | Q32AY2 | 3.25E-063 | 9 | 9 | 6.8 | Heme | Siderophores; siderophores-Fe complexes | *Shigella dysenteriae* | Proteobacteria | Bacteria |
| 3.A.1.14.19 | ABC | Q9K467 | SCO7218 | Q32AX9 | 2.72E-007 | 1 | 2 | 0.1 | Heme | Siderophores; siderophores-Fe complexes | *Shigella dysenteriae* | Proteobacteria | Bacteria |
| 3.A.1.14.2 | ABC | Q9S213 | SCO1787 | P23876 | 2.41E-055 | 9 | 10 | 8.05 | Ferric enterobactin | Siderophores; siderophores-Fe complexes | *Escherichia coli* | Proteobacteria | Bacteria |
| 3.A.1.14.5 | ABC | Q9EX41 | SCO0997 | Q56992 | 5.09E-052 | 9 | 9 | 7.9 | Heme | Siderophores; siderophores-Fe complexes | *Yersinia pestis* | Proteobacteria | Bacteria |
| 3.A.1.14.5 | ABC | Q9K469 | SCO7216 | Q56992 | 3.35E-045 | 9 | 9 | 7.95 | Heme | Siderophores; siderophores-Fe complexes | *Yersinia pestis* | Proteobacteria | Bacteria |
| 3.A.1.14.6 | ABC | Q9S214 | SCO1786 | Q9RCF4 | 1.29E-067 | 9 | 10 | 7.7 | Ferrous -vibriobactin/enterobactin | Siderophores; siderophores-Fe complexes | *Vibrio cholerae* | Proteobacteria | Bacteria |
| 3.A.1.14.12 | ABC | Q9L177 | SCO7499 | Q9L177 | 0 | 1 | 1 | 1 | Desferrioxamine B | Siderophores; siderophores-Fe complexes | *Streptomyces coelicolor* | Actinobacteria | Bacteria |
| 3.A.1.14.12 | ABC | Q9L178 | SCO7498 | Q9L178 | 0 | 18 | 18 | 18 | Desferrioxamine B | Siderophores; siderophores-Fe complexes | *Streptomyces coelicolor* | Actinobacteria | Bacteria |
| 3.A.1.14.12 | ABC | Q9L179 | SCO7400 | Q9L179 | 0 | 0 | 0 | 0 | Desferrioxamine B | Siderophores; siderophores-Fe complexes | *Streptomyces coelicolor* | Actinobacteria | Bacteria |
| 3.A.1.15.10 | ABC | Q9RJG1 | SCO0473 | Q5FA63 | 2.18E-025 | 1 | 1 | 0.6 | Mn^2+^/Zn^2+^ | Cations | *Neisseria gonorrhoeae* | Proteobacteria | Bacteria |
| 3.A.1.15.3 | ABC | Q9L2H6 | SCO2507 | O33704 | 5.14E-038 | 8 | 7 | 6.35 | Zn^2+^ | Cations | *Streptococcus pneumoniae* | Firmicutes | Bacteria |
| 3.A.1.15.4 | ABC | Q9RJF9 | SCO0475 | Q56955 | 3.41E-038 | 8 | 8 | 6.9 | Fe^2+^ and Mn^2+^ | Cations | *Yersinia pestis* | Proteobacteria | Bacteria |
| 3.A.1.17.1 | ABC | Q9KYY5 | SCO7544 | Q47539 | 8.89E-037 | 6 | 6 | 5.25 | Taurine | Organoanions (noncarboxylic) | *Escherichia coli* | Proteobacteria | Bacteria |
| 3.A.1.17.1 | ABC | Q9ADG9 | SCO6094 | Q47539 | 1.22E-019 | 6 | 6 | 5.15 | Taurine | Organoanions (noncarboxylic) | *Escherichia coli* | Proteobacteria | Bacteria |
| 3.A.1.18.1 | ABC | O54190 | SCO5961 | Q05594 | 4.31E-059 | 6 | 6 | 4.9 | Co^2+^ | Cations | *Salmonella typhimurium* | Proteobacteria | Bacteria |
| 3.A.1.18.1 | ABC | O54189 | SCO5960 | Q05595 | 3.47E-019 | 2 | 2 | 1.6 | Co^2+^ | Cations | *Salmonella typhimurium* | Proteobacteria | Bacteria |
| 3.A.1.18.1 | ABC | O54188 | SCO5959 | Q05598 | 1.47E-016 | 5 | 5 | 2.5 | Co^2+^ | Cations | *Salmonella typhimurium* | Proteobacteria | Bacteria |
| 3.A.1.20.1 | ABC | O54120 | SCO5924 | O54371 | 1.03E-016 | 12 | 6 | 5.05 | Fe^2+^ | Cations | *Treponema hyodysenteriae* | Spirochaetes | Bacteria |
| 3.A.1.21.1 | ABC | Q9EWN7 | SCO7690 | Q9Z375 | 5.58E-089 | 6 | 7 | 3.9 | Ferric-Yersiniabactin | Siderophores; siderophores-Fe complexes | *Yersinia pestis* | Proteobacteria | Bacteria |
| 3.A.1.21.2 | ABC | Q9EWN8 | SCO7689 | P63391 | 1.78E-094 | 6 | 6 | 6.75 | Ferric-carboxymycobactin | Siderophores; siderophores-Fe complexes | *Mycobacterium tuberculosis* | Actino-bacteria | Bacteria |
| 3.A.1.14.22 | ABC | Q9L074 | SCO2780 | Q9L074 | 0 | 1 | 1 | 1 | Desferrioxamine E | Siderophores; siderophores-Fe complexes | *Streptomyces coelicolor* | Actinobacteria | Bacteria |
| 3.A.1.14.22 | ABC | Q9S215 | SCO1785 | Q9S215 | 0 | 0 | 0 | 0 | Desferrioxamine E | Siderophores; siderophores-Fe complexes | *Streptomyces coelicolor* | Actinobacteria | Bacteria |
| 3.A.1.14.22 | ABC | Q9S214 | SCO1786 | Q9S214 | 0 | 9 | 9 | 9 | Desferrioxamine E | Siderophores; siderophores-Fe complexes | *Streptomyces coelicolor* | Actinobacteria | Bacteria |
| 3.A.1.14.22 | ABC | Q9S213 | SCO1787 | Q9S213 | 0 | 9 | 9 | 9 | Desferrioxamine E | Siderophores; siderophores-Fe complexes | *Streptomyces coelicolor* | Actinobacteria | Bacteria |
| 3.A.1.23.1 | ABC | Q9RKC8 | SCO3159 | Q79CJ1 | 5.05E-020 | 7 | 7 | 4.45 | Ni^2+^ | Cations | *Streptococcus salivarius* | Firmicutes | Bacteria |
| 3.A.1.23.2 | ABC | Q9RKC7 | SCO3160 | Q97JB9 | 6.22E-007 | 5 | 6 | 2.35 | Co^2+^ | Cations | *Clostridium acetobutylicum* | Firmicutes | Bacteria |
| 3.A.1.24.2 | ABC | Q9L1C4 | SCO1558 | O32168 | 2.48E-045 | 5 | 5 | 5.25 | L,D -Methionine | Amino acids & conjugates | *Bacillus subtilis* | Firmicutes | Bacteria |
| 3.A.1.27.4 | ABC | Q8CJZ6 | SCO2420 | Q0S7K3 | 4.43E-070 | 5 | 5 | 5.5 | Cholesterol | Other hydrophobic substances | *Rhodococcus sp.* | Actino-bacteria | Bacteria |
| 3.A.1.27.4 | ABC | Q9F357 | SCO2418 | Q0S7K1 | 8.42E-056 | 1 | 1 | 0.95 | Cholesterol | Other hydrophobic substances | *Rhodococcus sp.* | Actino-bacteria | Bacteria |
| 3.A.1.27.4 | ABC | Q9F356 | SCO2419 | Q0S7K2 | 1.16E-055 | 1 | 1 | 1 | Cholesterol | Other hydrophobic substances | *Rhodococcus sp.* | Actino-bacteria | Bacteria |
| 3.A.1.27.4 | ABC | Q9F358 | SCO2417 | Q0S7K1 | 8.02E-011 | 1 | 1 | 0.95 | Cholesterol | Other hydrophobic substances | *Rhodococcus sp.* | Actino-bacteria | Bacteria |
| 3.A.1.27.4 | ABC | Q9F361 | SCO2414 | Q0S7K2 | 2.47E-006 | 1 | 1 | 0.8 | Cholesterol | Other hydrophobic substances | *Rhodococcus sp.* | Actino-bacteria | Bacteria |
| 3.A.1.27.4 | ABC | Q9F359 | SCO2416 | Q0S7K2 | 2.10E-005 | 1 | 1 | 0.9 | Cholesterol | Other hydrophobic substances | *Rhodococcus sp.* | Actino-bacteria | Bacteria |
| 3.A.1.27.5 | ABC | Q9KXZ4 | SCO2421 | O53546 | 1.71E-070 | 5 | 6 | 4.5 | Steroid? | Unknown | *Mycobacterium tuberculosis* | Actino-bacteria | Bacteria |
| 3.A.1.32.1 | ABC | Q9KXJ7 | SCO2323 | Q9KXJ7 | 0 | 8 | 8 | 8 | Cobalamin precursor | Vitamins & vitamin or cofactor precursors | *Streptomyces coelicolor* | Actino-bacteria | Bacteria |
| 3.A.1.32.1 | ABC | Q9KXJ5 | SCO2325 | Q9KXJ5 | 4.24E-156 | 6 | 6 | 6.55 | Cobalamin precursor | Vitamins & vitamin or cofactor precursors | *Streptomyces coelicolor* | Actino-bacteria | Bacteria |
| 3.A.1.103.1 | ABC | Q9EWH9 | SCO2995 | Q48475 | 5.43E-018 | 6 | 6 | 4.95 | O-antigen | Lipids | *Klebsiella pneumoniae* | Proteobacteria | Bacteria |
| 3.A.1.103.1 | ABC | Q9X7V2 | SCO6757 | Q48475 | 3.19E-017 | 6 | 6 | 5.15 | O-antigen | Lipids | *Klebsiella pneumoniae* | Proteobacteria | Bacteria |
| 3.A.1.105.1 | ABC | Q9XA53 | SCO3825 | P32011 | 3.65E-074 | 6 | 6 | 6.3 | Multiple drugs | Multiple Drugs | *Streptomyces peucetius* | Actino-bacteria | Bacteria |
| 3.A.1.105.1 | ABC | Q9X8J6 | SCO3417 | P32011 | 3.97E-033 | 6 | 6 | 5.6 | Multiple drugs | Multiple Drugs | *Streptomyces peucetius* | Actino-bacteria | Bacteria |
| 3.A.1.105.1 | ABC | Q9S2B0 | SCO1720 | P32011 | 5.30E-027 | 6 | 6 | 5.7 | Multiple drugs | Multiple Drugs | *Streptomyces peucetius* | Actino-bacteria | Bacteria |
| 3.A.1.105.1 | ABC | Q9F2Y7 | SCO4360 | P32011 | 4.09E-024 | 6 | 6 | 6 | Multiple drugs | Multiple Drugs | *Streptomyces peucetius* | Actino-bacteria | Bacteria |
| 3.A.1.105.2 | ABC | Q9RKS0 | SCO2258 | Q53717 | 7.94E-125 | 6 | 6 | 5.8 | Oleandomycin | Specific Drugs | *Streptomyces antibioticus* | Actino-bacteria | Bacteria |
| 3.A.1.105.2 | ABC | Q9F384 | SCO4404 | Q53717 | 1.46E-032 | 6 | 6 | 5.3 | Oleandomycin | Specific Drugs | *Streptomyces antibioticus* | Actino-bacteria | Bacteria |
| 3.A.1.105.2 | ABC | Q9ADK3 | SCO4964 | Q53717 | 1.66E-031 | 6 | 6 | 4.9 | Oleandomycin | Specific Drugs | *Streptomyces antibioticus* | Actino-bacteria | Bacteria |
| 3.A.1.105.2 | ABC | Q9L1F2 | SCO5453 | Q53717 | 8.34E-006 | 6 | 6 | 5 | Oleandomycin | Specific Drugs | *Streptomyces antibioticus* | Actino-bacteria | Bacteria |
| 3.A.1.105.3 | ABC | Q9K4C9 | SCO5382 | Q70J76 | 1.08E-048 | 6 | 6 | 5.85 | Multiple drugs | Multiple Drugs | *Streptomyces griseus* | Actino-bacteria | Bacteria |
| 3.A.1.105.4 | ABC | Q7AKK5 | SCO4074 | Q4VWC7 | 0.000209431 | 6 | 6 | 4.45 | Pyoluteorin | Specific Drugs | *Pseudomonas sp.* | Proteobacteria | Bacteria |
| 3.A.1.106.2 | ABC | Q9L1F4 | SCO5451 | Q2G2M9 | 5.51E-075 | 6 | 5 | 4.55 | Multiple drugs | Multiple Drugs | *Staphylococcus aureus* | Firmicutes | Bacteria |
| 3.A.1.106.2 | ABC | Q9L2K4 | SCO0700 | Q2G2M9 | 4.10E-073 | 6 | 5 | 4.7 | Multiple drugs | Multiple Drugs | *Staphylococcus aureus* | Firmicutes | Bacteria |
| 3.A.1.106.3 | ABC | Q9EWY2 | SCO1144 | Q8G7R7 | 1.56E-103 | 6 | 5 | 4.75 | Multiple Drugs (Antimicrobrials, nisin, polymyxin) | Multiple Drugs | *Bifidobacterium longum* | Actino-bacteria | Bacteria |
| 3.A.1.106.3 | ABC | Q9KZE5 | SCO7008 | Q8G7R7 | 7.72E-092 | 6 | 5 | 4.2 | Multiple Drugs (Antimicrobrials, nisin, polymyxin) | Multiple Drugs | *Bifidobacterium longum* | Actino-bacteria | Bacteria |
| 3.A.1.106.4 | ABC | Q9L1F5 | SCO5450 | A4QD95 | 6.68E-091 | 6 | 12 | 4.9 | Multiple drugs | Multiple Drugs | *Corynebacterium glutamicum* | Actino-bacteria | Bacteria |
| 3.A.1.106.6 | ABC |  | SCO2763 |  |  |  |  |  | Putative ATPase and permease of DesE |  | *Sco* |  | Bacteria |
| 3.A.1.111.3 | ABC | Q9RJT6 | SCO0374 | Q03203 | 2.02E-044 | 5 | 5 | 3.8 | Nisin | Peptides | *Lactococcus lactis* | Firmicutes | Bacteria |
| 3.A.1.111.4 | ABC | Q9RJD6 | SCO0756 | Q5TLL2 | 7.79E-056 | 6 | 6 | 4.5 | Multiple drugs - Bacteriocin (tetracycline, penicillin, triclosan) | Multiple Drugs | *Streptococcus mutans* | Firmicutes | Bacteria |
| 3.A.1.112.1 | ABC | Q9RJD7 | SCO0755 | Q03727 | 1.58E-079 | 6 | 7 | 3.8 | Heptadecapeptide | Peptides | *Streptococcus pneumoniae* | Firmicutes | Bacteria |
| 3.A.1.117.2 | ABC | Q9ZNB0 | SCO0742 | O32748 | 9.47E-084 | 6 | 5 | 5.15 | Hop | Specific Drugs | *Lactobacillus brevis* | Firmicutes | Bacteria |
| 3.A.1.119.1 | ABC | Q9L1F7 | SCO5448 | Q54204 | 3.91E-129 | 6 | 4 | 3.4 | Multiple drugs - 5-Hydroxystreptomycin | Multiple Drugs | *Streptomyces glaucescens* | Actino-bacteria | Bacteria |
| 3.A.1.119.1 | ABC | Q9KZJ7 | SCO1148 | Q54204 | 3.25E-117 | 6 | 4 | 3.85 | Multiple drugs - 5-Hydroxystreptomycin | Multiple Drugs | *Streptomyces glaucescens* | Actino-bacteria | Bacteria |
| 3.A.1.119.1 | ABC | Q9L1F6 | SCO5449 | Q54203 | 7.80E-090 | 6 | 6 | 5.5 | Multiple drugs - 5-Hydroxystreptomycin | Multiple Drugs | *Streptomyces glaucescens* | Actino-bacteria | Bacteria |
| 3.A.1.119.1 | ABC | Q8CK38 | SCO1147 | Q54203 | 1.38E-074 | 6 | 6 | 4.9 | Multiple drugs - 5-Hydroxystreptomycin | Multiple Drugs | *Streptomyces glaucescens* | Actino-bacteria | Bacteria |
| 3.A.1.119.1 | ABC | Q9RK15 | SCO0491 | Q54203 | 1.63E-040 | 5 | 6 | 4.6 | Multiple drugs - 5-Hydroxystreptomycin | Multiple Drugs | *Streptomyces glaucescens* | Actino-bacteria | Bacteria |
| 3.A.1.119.3 | ABC | Q9RK13 | SCO0493 | O85018 | 8.42E-144 | 6 | 9 | 5.7 | Exochelin | Siderophores; siderophores-Fe complexes | *Mycobacterium smegmatis* | Actino-bacteria | Bacteria |
| 3.A.1.122.5 | ABC | Q9AD76 | SCO4935 | A4YGY2 | 5.34E-009 | 5 | 4 | 2.45 | Unknown | Unknown | *Metallosphaera sedula* | Crenarchaeota | Archaea |
| 3.A.1.123.2 | ABC | Q9Z4Z6 | SCO3235 | Q93GF4 | 7.34E-091 | 6 | 7 | 5.15 | Aurecoin | Specific Drugs | *Staphylococcus aureus* | Firmicutes | Bacteria |
| 3.A.1.125.1 | ABC | Q9AK94 | SCO1368 | P75958 | 4.03E-005 | 4 | 4 | 2.95 | Lipoprotein | Lipids | *Escherichia coli* | Proteobacteria | Bacteria |
| 3.A.1.125.3 | ABC | Q9K3M9 | SCO1031 | Q2J9P4 | 7.57E-145 | 10 | 10 | 10.1 | Lipoprotein | Lipids | *Frankia sp.* | Actino-bacteria | Bacteria |
| 3.A.1.125.3 | ABC | Q9KZ59 | SCO3090 | Q2J9P4 | 1.57E-134 | 10 | 10 | 10.1 | Lipoprotein | Lipids | *Frankia sp.* | Actino-bacteria | Bacteria |
| 3.A.1.125.3 | ABC | Q9F2P0 | SCO3110 | Q2J9P4 | 4.24E-127 | 10 | 10 | 9 | Lipoprotein | Lipids | *Frankia sp.* | Actino-bacteria | Bacteria |
| 3.A.1.125.3 | ABC | Q8CJV7 | SCO3754 | Q2J9P4 | 5.60E-038 | 10 | 10 | 5.05 | Lipoprotein | Lipids | *Frankia sp.* | Actino-bacteria | Bacteria |
| 3.A.1.125.3 | ABC | Q9L0V2 | SCO3753 | Q2J9P4 | 1.27E-011 | 13 | 10 | 8.4 | Lipoprotein | Lipids | *Frankia sp.* | Actino-bacteria | Bacteria |
| 3.A.1.125.3 | ABC | Q8CJN2 | SCO6365 | Q2J9P4 | 9.38E-007 | 10 | 10 | 4.8 | Lipoprotein | Lipids | *Frankia sp.* | Actino-bacteria | Bacteria |
| 3.A.1.127.1 | ABC | Q53819 | SCO6684 | Q07638 | 2.39E-111 | 4 | 4 | 3.75 | Extracellular peptidic | Peptides | *Streptomyces griseus* | Actino-bacteria | Bacteria |
| 3.A.1.127.1 | ABC | O88039 | SCO6683 | Q07639 | 1.86E-099 | 2 | 6 | 1.75 | Extracellular peptidic | Peptides | *Streptomyces griseus* | Actino-bacteria | Bacteria |
| 3.A.1.129.1 | ABC | Q9ZBY5 | SCO3947 | P29018 | 1.55E-088 | 12 | 6 | 5.3 | Cysteine/ Glutathione | Amino acids & conjugates | *Escherichia coli* | Proteobacteria | Bacteria |
| 3.A.1.134.3 | ABC | Q9XAA6 | SCO0819 | O34741 | 7.36E-005 | 10 | 10 | 3.7 | Bacitracin | Specific Drugs | *Bacillus subtilis* | Firmicutes | Bacteria |
| 3.A.1.135.2 | ABC | Q9L2F2 | SCO2464 | Q9A1K4 | 4.32E-106 | 4 | 5 | 2.75 | Multidrug | Multiple Drugs | *Streptococcus pyogenes* | Firmicutes | Bacteria |
| 3.A.1.135.2 | ABC | Q9L2F3 | SCO2463 | Q9A1K5 | 6.77E-098 | 6 | 6 | 5.9 | Multidrug | Multiple Drugs | *Streptococcus pyogenes* | Firmicutes | Bacteria |
| 3.A.1.140.1 | ABC | Q9L1S7 | SCO2968 | P0AC31 | 1.11E-009 | 4 | 4 | 3.25 | Unknown | Unknown | *Escherichia coli* | Proteobacteria | Bacteria |
| 3.A.1.141.1 | ABC | Q9KZQ8 | SCO2895 | P74757 | 2.22E-006 | 6 | 5 | 4.45 | Multiple drugs - ethyl viologen | Multiple Drugs | *Synechocystis sp.* | Cyanobacteria | Bacteria |
| 3.A.1.141.2 | ABC | Q9KZQ7 | SCO2896 | Q8R6Q5 | 8.70E-012 | 6 | 6 | 5.3 | Multiple drugs | Multiple Drugs | *Thermoanaerobacter tengcongensis* | Firmicutes | Bacteria |
| 3.A.1.142.1 | ABC | Q9F340 | SCO5130 | Q8DNC0 | 8.58E-017 | 6 | 6 | 5.65 | Glycolipid | Lipids | *Streptococcus pneumoniae* | Firmicutes | Bacteria |
| 3.A.1.142.1 | ABC | Q9L007 | SCO2306 | Q8DNC0 | 2.27E-016 | 6 | 6 | 4.8 | Glycolipid | Lipids | *Streptococcus pneumoniae* | Firmicutes | Bacteria |
| 3.A.1.204.11 | ABC | Q9AJX1 | SCO1806 | A9SCA8 | 3.10E-035 | 7 | 7 | 3.25 | Unknown (pigment?) | Unknown | *Physcomitrella patens* | Bryophyta | Eukarya |
| 3.A.1.210.2 | ABC | Q93RX7 | SCO6295 | Q02592 | 1.58E-073 | 6 | 10 | 4.1 | Phytochelins and Cadmium phytochelin | Cations | *Schizosaccharomyces pombe* | Ascomycota | Eukarya |
| 3.A.2.1.1 | F-ATPase | Q9K4D8 | SCO5367 | P0AB98 | 4.91E-012 | 7 | 6 | 3.95 | H^+^ | Cations | *Escherichia coli* | Proteobacteria | Bacteria |
| 3.A.2.1.1 | F-ATPase | Q9K4D7 | SCO5369 | P0ABA0 | 1.00E-011 | 1 | 1 | 0.9 | H^+^ | Cations | *Escherichia coli* | Proteobacteria | Bacteria |
| 3.A.2.1.2 | F-ATPase | P0A304 | SCO5368 | P21905 | 3.26E-006 | 2 | 2 | 1.3 | Na^+^ | Cations | *Propionigenium modestum* | Fusobacteria | Bacteria |
| 3.A.3.5.18 | P-ATPase | Q9K3L4 | SCO1046 | O32220 | 2.88E-173 | 8 | 8 | 5.65 | Cu^2+/+^ | Cations | *Bacillus subtilis* | Firmicutes | Bacteria |
| 3.A.3.5.18 | P-ATPase | Q9RDJ4 | SCO2731 | O32220 | 2.51E-172 | 8 | 8 | 5 | Cu^2+/+^ | Cations | *Bacillus subtilis* | Firmicutes | Bacteria |
| 3.A.3.5.19 | P-ATPase | Q9RCV1 | SCO0860 | Q7A3E6 | 1.25E-140 | 8 | 7 | 4.15 | Cu^2+/+^, Fe ^2+/3+^, Pb^2+^ | Cations | *Staphylococcus aureus* | Firmicutes | Bacteria |
| 3.A.3.6.9 | P-ATPase | Q9ZBF3 | SCO6460 | O31688 | 4.93E-119 | 5 | 6 | 3.95 | Zn^2+^ | Cations | *Bacillus subtilis* | Firmicutes | Bacteria |
| 3.A.3.7.1 | P-ATPase | Q9X8Z9 | SCO3717 | P03960 | 0 | 7 | 7 | 5.6 | K^+^ | Cations | *Escherichia coli* | Proteobacteria | Bacteria |
| 3.A.3.7.1 | P-ATPase | Q9X900 | SCO3718 | P03959 | 2.75E-132 | 10 | 12 | 8.6 | K^+^ | Cations | *Escherichia coli* | Proteobacteria | Bacteria |
| 3.A.3.7.1 | P-ATPase | Q9X8Z8 | SCO3716 | P03961 | 2.35E-023 | 1 | 1 | 1 | K^+^ | Cations | *Escherichia coli* | Proteobacteria | Bacteria |
| 3.A.3.23.1 | P-ATPase | Q9Z4W5 | SCO3216 | Q9KXM5 | 0 | 10 | 10 | 8.45 | Cation | Cations | *Streptomyces coelicolor* | Actino-bacteria | Bacteria |
| 3.A.3.23.1 | P-ATPase | Q9KXM5 | SCO4332 | Q9KXM5 | 0 | 10 | 10 | 9.5 | Cation | Cations | *Streptomyces coelicolor* | Actino-bacteria | Bacteria |
| 3.A.3.25.1 | P-ATPase | Q9RJ01 | SCO0164 | Q9RJ01 | 0 | 4 | 4 | 4.55 | Cation | Cations | *Streptomyces coelicolor* | Actino-bacteria | Bacteria |
| 3.A.5.2.2 | Sec | P46785 | SCO4722 | P0A5Z2 | 2.16E-153 | 10 | 10 | 8.85 | Preprotein | Proteins | *Mycobacterium tuberculosis* | Actino-bacteria | Bacteria |
| 3.A.5.2.2 | Sec | Q9ZBP9 | SCO5580 | P66842 | 1.89E-112 | 1 | 1 | 1 | Protein | Proteins | *Mycobacterium tuberculosis* | Actino-bacteria | Bacteria |
| 3.A.5.2.2 | Sec | Q53955 | SCO1516 | Q50634 | 1.17E-072 | 5 | 5 | 3 | Protein | Proteins | *Mycobacterium tuberculosis* | Actino-bacteria | Bacteria |
| 3.A.5.2.2 | Sec | Q53956 | SCO1515 | Q50635 | 1.08E-061 | 6 | 6 | 3.85 | Protein | Proteins | *Mycobacterium tuberculosis* | Actino-bacteria | Bacteria |
| 3.A.5.2.2 | Sec | Q9Z521 | SCO1944 | P66791 | 3.36E-013 | 2 | 3 | 1.85 | Protein | Proteins | *Mycobacterium tuberculosis* | Actino-bacteria | Bacteria |
| 3.A.5.2.2 | Sec | P0A4G8 | SCO4646 | P0A5Z0 | 1.40E-011 | 1 | 1 | 1 | Preprotein | Proteins | *Mycobacterium tuberculosis* | Actino-bacteria | Bacteria |
| 3.A.5.2.2 | Sec | Q9L292 | SCO1517 | P65025 | 3.91E-010 | 1 | 1 | 0.85 | Protein | Proteins | *Mycobacterium tuberculosis* | Actino-bacteria | Bacteria |
| 3.A.7.13.1 | 3.A.7 | O69886 | SCO5598 | Q7CM88 | 6.99E-016 | 2 | 1 | 1 | T-strand? | Nucleic acids | *Bacillus anthracis* | Firmicutes | Bacteria |
| 3.A.7.13.1 | 3.A.7 | O69885 | SCO5597 | Q7CM88 | 3.88E-010 | 2 | 1 | 0.9 | T-strand? | Nucleic acids | *Bacillus anthracis* | Firmicutes | Bacteria |
| 3.A.7.13.1 | 3.A.7 | O69887 | SCO5599 | Q7CM88 | 9.41E-007 | 2 | 1 | 1.05 | T-strand? | Nucleic acids | *Bacillus anthracis* | Firmicutes | Bacteria |
| 3.A.7.15.1 | 3.A.7 | Q9KY92 | SCO5010 | Q9XC05 | 1.03E-009 | 5 | 5 | 2.15 | Fibril (pilus) | Proteins | *Actinobacillus actinomycetemcomitans* | Proteobacteria | Bacteria |
| 3.A.10.2.2 | H+-PPase | Q9X913 | SCO3547 | Q6BCL0 | 0 | 17 | 17 | 17.4 | H^+^-pyrophosphatase | Cations | *Streptomyces coelicolor* | Actino-bacteria | Bacteria |
| 3.A.11.1.1 | DNA-T | Q9RDL9 | SCO2567 | P39695 | 5.02E-035 | 6 | 12 | 1.9 | DNA | Nucleic acids | *Bacillus subtilis* | Firmicutes | Bacteria |
| 3.A.12.1.1 | S-DNA-T | O86810 | SCO5750 | P21458 | 4.97E-130 | 4 | 4 | 1.35 | Septum DNA | Nucleic acids | *Bacillus subtilis* | Firmicutes | Bacteria |
| 3.A.15.3.1 | MTB | Q9XAP1 | SCO4549 | O68433 | 2.09E-012 | 8 | 6 | 4.4 | Fimbrilin | Proteins | *Legionella pneumophila* | Proteobacteria | Bacteria |
| 3.B.1.1.2 | NaT-DC | O86517 | SCO5535 | Q57079 | 8.91E-171 | 1 | 1 | 0.9 | Na^+^ | Cations | *Veillonella parvula* | Firmicutes | Bacteria |
| 3.B.1.1.5 | NaT-DC | Q9X4K7 | SCO4926 | Q9V0A4 | 4.45E-173 | 1 | 3 | 0.9 | Na^+^ | Cations | *Pyrococcus abyssi* | Euryarchaeota | Archaea |
| 3.B.1.1.5 | NaT-DC | Q93S05 | SCO6284 | Q9V0A4 | 6.06E-170 | 1 | 3 | 0.9 | Na^+^ | Cations | *Pyrococcus abyssi* | Euryarchaeota | Archaea |
| 3.B.1.1.5 | NaT-DC | Q9L077 | SCO2776 | Q9V0A4 | 2.67E-076 | 1 | 3 | 0.9 | Na^+^ | Cations | *Pyrococcus abyssi* | Euryarchaeota | Archaea |
| 3.D.1.1.1 | NDH | Q9XAR7 | SCO4575 | P0AFF0 | 2.86E-062 | 14 | 14 | 9.5 | NADH | Electrons | *Escherichia coli* | Proteobacteria | Bacteria |
| 3.D.1.2.1 | NDH | Q9XAR5 | SCO4573 | P29924 | 1.23E-093 | 16 | 16 | 10.7 | NADH | Electrons | *Paracoccus denitrificans* | Proteobacteria | Bacteria |
| 3.D.1.2.1 | NDH | Q9XAR6 | SCO4574 | P29925 | 5.04E-091 | 14 | 14 | 7.15 | NADH | Electrons | *Paracoccus denitrificans* | Proteobacteria | Bacteria |
| 3.D.1.2.1 | NDH | Q9F2V4 | SCO4607 | P29925 | 1.95E-077 | 14 | 14 | 7.35 | NADH | Electrons | *Paracoccus denitrificans* | Proteobacteria | Bacteria |
| 3.D.1.2.1 | NDH | Q9F2V6 | SCO4605 | P29923 | 2.23E-015 | 3 | 3 | 2.8 | NADH | Electrons | *Paracoccus denitrificans* | Proteobacteria | Bacteria |
| 3.D.1.3.1 | NDH | Q9XAR4 | SCO4572 | Q56226 | 1.27E-019 | 3 | 2 | 2.15 | NADH | Electrons | *Paracoccus denitrificans* | Proteobacteria | Bacteria |
| 3.D.1.5.1 | NDH | Q9XAR1 | SCO4569 | Q746T2 | 5.77E-082 | 9 | 8 | 5.65 | H+ - NADH | Electrons | *Geobacter sulfurreducens* | Proteobacteria | Bacteria |
| 3.D.1.5.1 | NDH | Q9F2V9 | SCO4602 | Q746T2 | 2.80E-057 | 8 | 8 | 5.15 | H+ - NADH | Electrons | *Geobacter sulfurreducens* | Proteobacteria | Bacteria |
| 3.D.1.6.1 | NDH | Q9F2W1 | SCO4600 | P42026 | 2.60E-047 | 2 | 2 | 0.85 | H+ - NADH | Electrons | *Bos taurus* | Chordata | Eukarya |
| 3.D.1.7.1 | NDH | Q9XAR3 | SCO4571 | Q0P858 | 1.51E-014 | 5 | 5 | 4.8 | NADH: uniquinone oxidoreductase | Electrons | *Campylobacter jejuni* | Proteobacteria | Bacteria |
| 3.D.1.8.1 | NDH | Q9F2W2 | SCO4599 | P56751 | 2.14E-020 | 3 | 3 | 2.9 | NAD(P)H-quinone oxidoreductase | Electrons | *Arabidopsis thaliana* | Angiosperms | Eukarya |
| 3.D.1.8.1 | NDH | Q9XAQ4 | SCO4562 | P56751 | 3.76E-020 | 3 | 3 | 3.2 | NAD(P)H-quinone oxidoreductase | Electrons | *Arabidopsis thaliana* | Angiosperms | Eukarya |
| 3.D.2.1.1 | PTH | Q9F3D5 | SCO7622 | P0AB67 | 1.60E-179 | 9 | 10 | 6.35 | H+ | Cations | *Escherichia coli* | Proteobacteria | Bacteria |
| 3.D.2.1.1 | PTH | Q9F3D4 | SCO7623 | P07001 | 1.21E-168 | 4 | 5 | 3.2 | H+ | Cations | *Escherichia coli* | Proteobacteria | Bacteria |
| 3.D.3.4.1 | QCR | Q9X808 | SCO2150 | P46913 | 0.000602691 | 2 | 3 | 0.9 | Electrons – Menaquinone | Electrons | *Bacillus subtilis* | Firmicutes | Bacteria |
| 3.D.4.3.1 | COX | Q9ZBY6 | SCO3946 | O54596 | 5.35E-018 | 9 | 9 | 6.9 | Electrons-cytochrom oxidase | Electrons | *Halobacterium saliNa+rium* | Euryarchaeota | Archaea |
| 3.D.4.4.1 | COX | Q9XAC6 | SCO1930 | P12946 | 1.63E-007 | 8 | 8 | 5.65 | Electrons-cytochrom oxidase | Electrons | *Bacillus subtilis* | Firmicutes | Bacteria |
| 3.D.4.4.2 | COX | Q9K451 | SCO7234 | Q79VD7 | 0 | 12 | 12 | 10.85 | Electrons-cytochrom oxidase | Electrons | *Corynebacterium glutamicum* | Actino-bacteria | Bacteria |
| 3.D.4.4.2 | COX | Q9X813 | SCO2155 | Q79VD7 | 0 | 12 | 12 | 11.15 | Electrons-cytochrom oxidase | Electrons | *Corynebacterium glutamicum* | Actino-bacteria | Bacteria |
| 3.D.4.4.2 | COX | Q9X809 | SCO2151 | Q9AEL8 | 2.08E-061 | 5 | 5 | 4.85 | Electrons-cytochrom oxidase | Electrons | *Corynebacterium glutamicum* | Actino-bacteria | Bacteria |
| 3.D.4.4.2 | COX | Q9X814 | SCO2156 | Q8NNK2 | 5.47E-038 | 3 | 3 | 2.1 | Electrons-cytochrom oxidase | Electrons | *Corynebacterium glutamicum* | Actino-bacteria | Bacteria |
| 3.D.4.4.2 | COX | Q9X812 | SCO2154 | Q8NNK3 | 4.06E-013 | 4 | 4 | 3.1 | Electrons-cytochrom oxidase | Electrons | *Corynebacterium glutamicum* | Actino-bacteria | Bacteria |
| 3.D.4.4.2 | COX | Q9K4J0 | SCO7235 | Q8NNK3 | 7.98E-011 | 2 | 4 | 1.5 | Electrons-cytochrom oxidase | Electrons | *Corynebacterium glutamicum* | Actino-bacteria | Bacteria |
| 3.D.4.5.1 | COX | Q9XAC2 | SCO1934 | P0AEA5 | 1.21E-035 | 9 | 9 | 7.4 | Electron - Quinol oxidase | Electrons | *Escherichia coli* | Proteobacteria | Bacteria |
| 3.D.5.1.1 | Na+-NDH | Q9L131 | SCO7428 | Q56584 | 2.81E-007 | 1 | 2 | 0.05 | Na^+^- quinone reductase | Electrons | *Vibrio* | Proteobacteria | Bacteria |
| 3.D.9.1.1 | F420H2DH | Q9F2V5 | SCO4606 | Q9P9F5 | 7.93E-081 | 14 | 18 | 8.1 | Electrons | Electrons | *Methanosarcina mazei* | Euryarchaeota | Archaea |
| 3.D.9.1.1 | F420H2DH | Q9F2V3 | SCO4608 | Q9P9F3 | 6.81E-053 | 14 | 13 | 10.65 | Electrons | Electrons | *Methanosarcina mazei* | Euryarchaeota | Archaea |
| 3.D.9.1.1 | F420H2DH | Q9F2V7 | SCO4604 | Q9P9F7 | 7.67E-020 | 5 | 5 | 4.65 | Electrons | Electrons | *Methanosarcina mazei* | Euryarchaeota | Archaea |
| 3.E.2.2.2 | PRC | Q9FC66 | SCO7120 | Q57038 | 1.73E-028 | 8 | 5 | 2.15 | Electron | Electrons | *Synechocystis sp.* | Cyanobacteria | Bacteria |
| 3.E.2.2.2 | PRC | Q9X806 | SCO2148 | Q57038 | 4.01E-028 | 8 | 5 | 2.7 | Electron | Electrons | *Synechocystis sp.* | Cyanobacteria | Bacteria |
| 3.E.2.2.2 | PRC | Q9K4I9 | SCO7236 | Q57038 | 1.68E-025 | 8 | 5 | 1.7 | Electron | Electrons | *Synechocystis sp.* | Cyanobacteria | Bacteria |
| 4.A.1.1.3 | Glc | Q9RL52 | SCO0434 | P19642 | 5.01E-180 | 10 | 8 | 7.25 | Maltose | Sugars & polyols | *Escherichia coli* | Proteobacteria | Bacteria |
| 4.A.1.1.5 | Glc | Q9S2H5 | SCO2906 | Q8GBT7 | 0 | 12 | 12 | 9.6 | N-Acetylglucosamine + xylose | Amines, amides, polyamines, & organocations | *Streptomyces olivaceoviridis* | Actino-bacteria | Bacteria |
| 4.A.1.1.5 | Glc | Q9S2H4 | SCO2907 | Q8GBT7 | 4.47E-160 | 12 | 12 | 6.85 | N-Acetylglucosamine + xylose | Amines, amides, polyamines, & organocations | *Streptomyces olivaceoviridis* | Actino-bacteria | Bacteria |
| 4.A.2.1.13 | Fru | Q9KYU9 | SCO3196 | Q0S1N2 | 1.32E-171 | 8 | 8 | 8.8 | Fructose | Sugars & polyols | *Rhodococcus sp.* | Actino-bacteria | Bacteria |
| 4.A.7.1.1 | L-Asc | Q9S1Z0 | SCO0136 | P39301 | 5.38E-036 | 11 | 11 | 6.9 | L-ascorbate | Vitamins & vitamin or cofactor precursors | *Escherichia coli* | Proteobacteria | Bacteria |
| 4.B.1.1.3 | 4.B.1 | Q9EWJ7 | SCO1442 | Q9EWJ7 | 1.00E-118 | 4 | 4 | 4.65 | Nicotinamide | Nucleic acids | *Streptomyces coelicolor* | Actino-bacteria | Bacteria |
| 4.C.1.1.4 | 4.C.1 | Q9ZBW6 | SCO6552 | P69451 | 2.37E-030 | 3 | 2 | 0.25 | Long chain fatty acids | Lipids | *Escherichia coli* | Proteobacteria | Bacteria |
| 5.A.1.2.1 | DsbD | Q9F2R6 | SCO4473 | P45706 | 1.89E-028 | 6 | 6 | 5.95 | Electrons | Electrons | *Bacillus subtilis* | Firmicutes | Bacteria |
| 5.A.1.2.2 | DsbD | Q93JD3 | SCO5994 | F4FC13 | 2.53E-067 | 7 | 11 | 7.35 | Electrons | Electrons | *Verrucosispora maris* | Actino-bacteria | Bacteria |
| 5.A.1.2.2 | DsbD | Q93JD5 | SCO5992 | F4FC13 | 2.97E-029 | 4 | 11 | 3.45 | Electrons | Electrons | *Verrucosispora maris* | Actino-bacteria | Bacteria |
| 5.A.3.1.1 | PMO | O86714 | SCO6532 | P11350 | 3.60E-028 | 5 | 5 | 4.5 | Electrons | Electrons | *Escherichia coli* | Proteobacteria | Bacteria |
| 5.A.3.1.1 | PMO | Q9RI29 | SCO0219 | P11350 | 2.01E-025 | 5 | 5 | 4.5 | Electrons | Electrons | *Escherichia coli* | Proteobacteria | Bacteria |
| 5.A.3.1.2 | PMO | Q9EWF6 | SCO4950 | P0AF32 | 1.09E-024 | 5 | 5 | 4.7 | Electrons - nitrate reductase | Electrons | *Escherichia coli* | Proteobacteria | Bacteria |
| 5.A.4.1.1 | 5.A.4 | Q9RCY7 | SCO0923 | Q65GF4 | 9.61E-101 | 1 | 1 | 0.9 | Electrons-succinate | Electrons | *Bacillus licheniformis* | Firmicutes | Bacteria |
| 5.A.4.1.1 | 5.A.4 | Q9X8N8 | SCO3382 | Q65GF4 | 2.11E-054 | 1 | 1 | 0.75 | Electrons-succinate | Electrons | *Bacillus licheniformis* | Firmicutes | Bacteria |
| 5.B.1.6.1 | gp91phox | Q9L1A8 | SCO1573 | Q9KN15 | 7.28E-017 | 6 | 7 | 3.3 | Electrons | Electrons | *Vibrio cholerae* | Proteobacteria | Bacteria |
| 8.A.21.2.1 | 8.A.21 | O69841 | SCO6053 | O59180 | 4.02E-056 | 1 | 3 | 0.85 | Stomatin | Proteins | *Pyrococcus horikoshii* | Euryarchaeota | Archaea |
| 8.A.21.2.1 | 8.A.21 | Q9K458 | SCO7227 | O59180 | 2.42E-029 | 1 | 3 | 0.75 | Stomatin | Proteins | *Pyrococcus horikoshii* | Euryarchaeota | Archaea |
| 8.A.21.2.1 | 8.A.21 | Q9X9Z6 | SCO1796 | O59180 | 2.61E-026 | 3 | 3 | 2.2 | Stomatin | Proteins | *Pyrococcus horikoshii* | Euryarchaeota | Archaea |
| 8.A.21.2.1 | 8.A.21 | Q9X9Z5 | SCO1797 | O59179 | 8.83E-005 | 2 | 7 | 2.15 | Stomatin | Proteins | *Pyrococcus horikoshii* | Euryarchaeota | Archaea |
| 9.A.10.2.3 | OFeT | Q9RKQ1 | SCO2277 | P75901 | 2.63E-048 | 7 | 6 | 4.4 | Fe^2+^ | Cations | *Escherichia coli* | Proteobacteria | Bacteria |
| 9.A.30.1.2 | 9.A.30 | Q9Z503 | SCO1963 | F0N2E7 | 1.17E-064 | 9 | 9 | 6 | Tellurite | Cations | *Neisseria meningitidis* | Proteobacteria | Bacteria |
| 9.A.30.1.2 | 9.A.30 | Q9RD18 | SCO0892 | F0N2E7 | 7.80E-062 | 9 | 9 | 6.55 | Tellurite | Cations | *Neisseria meningitidis* | Proteobacteria | Bacteria |
| 9.A.30.3.1 | 9.A.30 | Q9KY24 | SCO2366 | C9RH03 | 4.72E-083 | 9 | 9 | 6.45 | Tellurium | Cations | *Methanocaldococcus vulcanius* | Euryarchaeota | Archaea |
| 9.A.30.3.1 | 9.A.30 | Q9RJH8 | SCO0562 | C9RH03 | 2.85E-080 | 9 | 9 | 6.4 | Tellurium | Cations | *Methanocaldococcus vulcanius* | Euryarchaeota | Archaea |
| 9.A.30.3.1 | 9.A.30 | Q93RS9 | SCO6313 | C9RH03 | 1.30E-077 | 9 | 9 | 5.7 | Tellurium | Cations | *Methanocaldococcus vulcanius* | Euryarchaeota | Archaea |
| 9.A.40.2.1 | 9.A.40 | Q9FCB6 | SCO1251 | P54428 | 4.94E-050 | 3 | 4 | 2.45 | Co^2+^/Mg^2+^ | Cations | *Bacillus subtilis* | Firmicutes | Bacteria |
| 9.A.40.2.1 | 9.A.40 | Q9S1P5 | SCO0246 | P54428 | 1.48E-048 | 3 | 4 | 2.7 | Co^2+^/Mg^2+^ | Cations | *Bacillus subtilis* | Firmicutes | Bacteria |
| 9.A.40.2.1 | 9.A.40 | Q9F2L6 | SCO3765 | P54428 | 1.33E-047 | 3 | 4 | 2.5 | Co^2+^/Mg^2+^ | Cations | *Bacillus subtilis* | Firmicutes | Bacteria |
| 9.A.40.2.1 | 9.A.40 | Q9RKZ4 | SCO1436 | P54428 | 2.16E-047 | 3 | 4 | 2.85 | Co^2+^/Mg^2+^ | Cations | *Bacillus subtilis* | Firmicutes | Bacteria |
| 9.A.40.2.1 | 9.A.40 | Q9RKZ3 | SCO1435 | P54428 | 1.78E-046 | 3 | 4 | 2.8 | Co^2+^/Mg^2+^ | Cations | *Bacillus subtilis* | Firmicutes | Bacteria |
| 9.A.40.2.1 | 9.A.40 | Q9FCB5 | SCO1252 | P54428 | 7.82E-044 | 3 | 4 | 2.95 | Co^2+^/Mg^2+^ | Cations | *Bacillus subtilis* | Firmicutes | Bacteria |
| 9.A.40.2.1 | 9.A.40 | Q9F2L7 | SCO3764 | P54428 | 4.19E-042 | 3 | 4 | 3.1 | Co^2+^/Mg^2+^ | Cations | *Bacillus subtilis* | Firmicutes | Bacteria |
| 9.A.40.2.1 | 9.A.40 | Q8CJZ2 | SCO2534 | P54428 | 5.15E-036 | 3 | 4 | 1.7 | Co^2+^/Mg^2+^ | Cations | *Bacillus subtilis* | Firmicutes | Bacteria |
| 9.A.40.2.1 | 9.A.40 | Q9S1P6 | SCO0245 | P54428 | 1.57E-034 | 3 | 4 | 3.05 | Co^2+^/Mg^2+^ | Cations | *Bacillus subtilis* | Firmicutes | Bacteria |
| 9.A.42.1.1 | 9.A.42 | O86641 | SCO5721 | O53933 | 2.64E-005 | 1 | 1 | 0.9 | Protein | Proteins | *Mycobacterium tuberculosis* | Actino-bacteria | Bacteria |
| 9.A.44.1.1 | 9.A.44 | Q9L0T6 | SCO4508 | Q932J9 | 3.11E-077 | 2 | 2 | 0.4 | EsxA or EsxB | Proteins | *Staphylococcus aureus* | Firmicutes | Bacteria |
| 9.A.47.1.1 | 9.A.47 | Q9KY91 | SCO5011 | Q9S4A8 | 3.94E-007 | 4 | 5 | 2.9 | Pilus | Proteins | *Actinobacillus actinomycetemcomitans* | Proteobacteria | Bacteria |
| 9.A.49.1.1 | 9.A.49 | Q8CJV2 | SCO3934 | Q15HV3 | 2.22E-019 | 1 | 4 | 0.75 | Transfer DNA, DNA, transfer (possibly RNA since bacteria) | Nucleic acids | *Clostridium perfringens* | Firmicutes | Bacteria |
| 9.A.49.1.1 | 9.A.49 | Q9KYD8 | SCO4132 | Q15HU6 | 1.05E-007 | 1 | 1 | 0.75 | Transfer DNA, DNA, transfer (possibly RNA since bacteria) | Nucleic acids | *Clostridium perfringens* | Firmicutes | Bacteria |
