# Supplementary material for: Comparative genomics of transport proteins in developmental bacteria: Myxococcus xanthus and Streptomyces coelicolor
Source: BMC Microbiol. 2013 Dec 5;13:279. doi: 10.1186/1471-2180-13-279 (PMC3924187; doi:10.1186/1471-2180-13-279)
Supplement: Additional file 2: Table S2 — Mxa transport proteins. Detailed description of Mxa transport proteins and their homologues in TCDB, including comparison scores obtained via G-Blast and GSAT, substrate, substrate class, organism, phylum, and organismal domain. Proteins are organized from lowest to highest TC#. [file 1471-2180-13-279-S2.docx]

**Table S2. Mxa Transport proteins.** Detailed description of Mxa transport proteins and their homologues in TCDB, including comparison scores obtained via G-Blast and NCBI, substrate, substrate class, organism, phylum, and domain. Proteins are organized from lowest to highest TC#.

| **Hit TCID** | **Family Abrv.** | **Query Acc #** | **MXAN#** | **Hit Acc #** | **e-Value** | **Query TMS#** | **Hit TMS#** | **TM-Overlap Score** | **Substrate** | **Substrate Class** | **Organism** | **Phylum** | **Domain** |
| --- | --- | --- | --- | --- | --- | --- | --- | --- | --- | --- | --- | --- | --- |
| 1.A.1.2.11 | VIC | Q1D027 | MXAN_5859 | Q14721 | 1.15E-23 | 5 | 6 | 3.1 | K^+^ | Cations | *Homo sapiens* | Chordata | Eukarya |
| 1.A.6.2.2 | ENaC | Q1D7J8 | MXAN_3172 | Q27433 | 2.77E-05 | 1 | 2 | 0.8 | Na^+^ | Cations | *Caenorhabditis elegans* | Nematoda | Eukarya |
| 1.A.8.3.1 | MIP | Q1D2A0 | MXAN_5069 | P60844 | 1.20E-51 | 6 | 6 | 5.45 | Water | Water | *Escherichia coli* | Proteobacteria | Bacteria |
| 1.A.8.15.1 | MIP | Q1CZT0 | MXAN_5956 | Q92R43 | 3.39E-34 | 4 | 6 | 2.95 | As^3+^ | Cations | *Sinorhizobium meliloti* | Proteobacteria | Bacteria |
| 1.A.11.5.1 | Amt | Q1CZ61 | MXAN_6181 | O29285 | 1.31E-85 | 12 | 11 | 6.4 | Trimeric ammonia | Amines, amides, polyamines, & organocations | *Archaeoglobus fulgidus* | Euryarchaeota | Archaea |
| 1.A.23.2.1 | MscS | Q1D0J8 | MXAN_5687 | P0C0S1 | 6.37E-10 | 8 | 4 | 2.95 | Ion | Nonspecific ion | *Escherichia coli* | Proteobacteria | Bacteria |
| 1.A.23.3.1 | MscS | Q1DEP9 | MXAN_0606 | O34897 | 6.75E-34 | 9 | 2 | 2 | Ion | Nonspecific ion | *Bacillus subtilis* | Firmicutes | Bacteria |
| 1.A.23.5.1 | MscS | Q1D8K7 | MXAN_2799 | P71915 | 2.27E-20 | 5 | 5 | 4.2 | Cyclic nucleotides | Nucleotides | *Mycobacterium tuberculosis* | Actinobacteria | Bacteria |
| 1.A.23.4.5 | MscS | Q1D4Y2 | MXAN_4113 | P0AAT4 | 7.99E-85 | 4 | 4 | 3.5 | Ion | Nonspecific ion | *Escherichia coli* | Proteobacteria | Bacteria |
| 1.A.30.1.3 | Mot-Exb | Q1CWS3 | MXAN_7037 | P28612 | 3.42E-21 | 1 | 1 | 0.6 | H^+^ or Na^+^ | Cations | *Bacillus subtilis* | Firmicutes | Bacteria |
| 1.A.30.2.1 | Mot-Exb | Q1CX94 | MXAN_6862 | P0ABU7 | 1.40E-09 | 3 | 3 | 1.6 | Fe^3+^ | Cations | *Escherichia coli* | Proteobacteria | Bacteria |
| 1.A.30.2.1 | Mot-Exb | Q1DFL7 | MXAN_0275 | P0ABU7 | 2.34E-19 | 3 | 3 | 3.25 | Fe^3+^ | Cations | *Escherichia coli* | Proteobacteria | Bacteria |
| 1.A.30.2.2 | Mot-Exb | Q1D811 | MXAN_3003 | P0ABU9 | 9.03E-05 | 3 | 3 | 0.45 | Bacteriocin and protein | Toxins | *Escherichia coli* | Proteobacteria | Bacteria |
| 1.A.30.2.2 | Mot-Exb | Q1D3D8 | MXAN_4669 | P0ABU9 | 4.05E-15 | 3 | 3 | 2.75 | Bacteriocin and protein | Toxins | *Escherichia coli* | Proteobacteria | Bacteria |
| 1.A.30.2.2 | Mot-Exb | Q1CYB8 | MXAN_6483 | P0ABU9 | 5.70E-16 | 3 | 3 | 2.85 | Bacteriocin and protein | Toxins | *Escherichia coli* | Proteobacteria | Bacteria |
| 1.A.30.2.2 | Mot-Exb | Q1DE42 | MXAN_0818 | P0ABU9 | 2.68E-15 | 3 | 3 | 2.85 | Bacteriocin and protein | Toxins | *Escherichia coli* | Proteobacteria | Bacteria |
| 1.A.30.2.2 | Mot-Exb | Q1D0D3 | MXAN_5753 | P0ABU9 | 1.70E-35 | 3 | 3 | 3.1 | Bacteriocin and protein | Toxins | *Escherichia coli* | Proteobacteria | Bacteria |
| 1.A.30.2.2 | Mot-Exb | Q1DCB8 | MXAN_1448 | P0ABU9 | 3.16E-19 | 3 | 3 | 3.2 | Bacteriocin and protein | Toxins | *Escherichia coli* | Proteobacteria | Bacteria |
| 1.B.33.1.3 | OmpIP | Q1D5X3 | MXAN_3768 | P77774 | 3.93E-24 | 1 | 1 | 0.5 | Protein insertion | Proteins | *Escherichia coli* | Proteobacteria | Bacteria |
| 1.B.33.1.3 | OmpIP | Q1DAU0 | MXAN_1998 | P0AC02 | 1.20E-20 | 1 | 1 | 0.75 | Protein insertion | Proteins | *Escherichia coli* | Proteobacteria | Bacteria |
| 1.B.42.1.2 | OmpIP | Q1D894 | MXAN_2918 | P0ADC6 | 0.0001422 | 6 | 6 | 4.5 | Lipopolysaccharide | Carbohydrates | *Escherichia coli* | Proteobacteria | Bacteria |
| 2.A.1.2.3 | MFS | Q1D988 | MXAN_2566 | P32482 | 1.84E-96 | 12 | 12 | 11.85 | H^+^; multidrug; isopropyl β-thiogalactoside | Multiple drugs | *Pseudomonas aeruginosa* | Proteobacteria | Bacteria |
| 2.A.1.2.7 | MFS | Q1CVH5 | MXAN_7495 | P28246 | 2.25E-46 | 11 | 12 | 9.05 | H^+^ | Cations | *Escherichia coli* | Proteobacteria | Bacteria |
| 2.A.1.2.8 | MFS | Q1D695 | MXAN_3641 | P39843 | 4.72E-12 | 12 | 12 | 11.15 | H^+^ | Cations | *Bacillus subtilis* | Firmicutes | Bacteria |
| 2.A.1.2.25 | MFS | Q1CZY0 | MXAN_5906 | Q797E3 | 3.81E-15 | 12 | 12 | 9.95 | Purine base/nucleoside | Amino acids & conjugates | *Bacillus subtilis* | Firmicutes | Bacteria |
| 2.A.1.2.25 | MFS | Q1CZ10 | MXAN_6234 | Q797E3 | 4.21E-52 | 12 | 12 | 11.15 | Purine base/nucleoside | Amino acids & conjugates | *Bacillus subtilis* | Firmicutes | Bacteria |
| 2.A.1.2.37 | MFS | Q1D659 | MXAN_3677 | Q5WX21 | 6.30E-05 | 12 | 12 | 4.8 | Legiobactin | Siderophores; siderophores-Fe complexes | *Legionella pneumophila* | Proteobacteria | Bacteria |
| 2.A.1.2.38 | MFS | Q1D9B2 | MXAN_2542 | Q56RY7 | 1.90E-38 | 12 | 12 | 8.8 | Tetracycline-specific | Specific drugs | *Acinetobacter sp.* | Proteobacteria | Bacteria |
| 2.A.1.2.39 | MFS | Q1D6T2 | MXAN_3444 | Q5JAK9 | 2.57E-41 | 12 | 12 | 11.45 | Tetracycline-specific | Specific drugs | *Serratia marcescens* | Proteobacteria | Bacteria |
| 2.A.1.2.42 | MFS | Q1DA89 | MXAN_2212 | Q9KMQ3 | 9.59E-69 | 12 | 12 | 11 | Multidrug | Multiple drugs | *Vibrio cholerae* | Proteobacteria | Bacteria |
| 2.A.1.3.12 | MFS | Q1DFK1 | MXAN_0292 | P39886 | 4.64E-52 | 14 | 13 | 6.6 | H^+^ | Cations | *Streptomyces glaucescens* | Actinobacteria | Bacteria |
| 2.A.1.3.20 | MFS | Q1DBQ7 | MXAN_1664 | Q9RQ29 | 1.62E-48 | 14 | 14 | 12 | Fatty acid | Lipids | *Neisseria gonorrhoeae* | Proteobacteria | Bacteria |
| 2.A.1.3.26 | MFS | Q1D3J6 | MXAN_4609 | P36554 | 2.14E-67 | 14 | 14 | 11.6 | Novobiocin/deoxycholate exporting MDR | Drugs | *Escherichia coli* | Proteobacteria | Bacteria |
| 2.A.1.3.35 | MFS | Q1DG35 | MXAN_0105 | O32182 | 2.18E-89 | 12 | 14 | 9.35 | Novobiocin/deoxycholate exporting MDR | Drugs | *Bacillus subtilis* | Firmicutes | Bacteria |
| 2.A.1.3.35 | MFS | Q1DBZ5 | MXAN_1576 | O32182 | 5.83E-44 | 14 | 14 | 10.05 | Novobiocin/deoxycholate exporting MDR | Drugs | *Bacillus subtilis* | Firmicutes | Bacteria |
| 2.A.1.3.35 | MFS | Q1D2X6 | MXAN_4836 | O32182 | 8.05E-78 | 14 | 14 | 10.2 | Novobiocin/deoxycholate exporting MDR | Drugs | *Bacillus subtilis* | Firmicutes | Bacteria |
| 2.A.1.4.4 | MFS | Q1D8Z1 | MXAN_2663 | P09836 | 1.27E-13 | 13 | 12 | 8.75 | Hexose | Sugars & polyols | *Escherichia coli* | Proteobacteria | Bacteria |
| 2.A.1.6.4 | MFS | Q1CXB5 | MXAN_6841 | P0C0L7 | 8.59E-45 | 12 | 12 | 10 | H^+^/Na^+^; taurine, ectoine, pipecolate, proline-betaine, N,N-dimethylglycine, carnitine, and 1-carboxymethyl-pyridinium | Amino acids & conjugates | *Escherichia coli* | Proteobacteria | Bacteria |
| 2.A.1.15.7 | MFS | Q1D5L3 | MXAN_3880 | O34691 | 8.11E-14 | 12 | 12 | 11.9 | Niacin/nicotinamide | Vitamins & vitamin or cofactor precursors | *Bacillus subtilis* | Firmicutes | Bacteria |
| 2.A.1.21.1 | MFS | Q1DAA8 | MXAN_2193 | P95827 | 1.27E-10 | 4 | 12 | 3.25 | Macrolide | Multiple drugs | *Streptococcus pyogenes/Streptococcus pneumoniae* | Firmicutes | Bacteria |
| 2.A.1.21.1 | MFS | Q1D3S2 | MXAN_4533 | P95827 | 3.32E-24 | 12 | 12 | 9.2 | Macrolide | Multiple drugs | *Streptococcus pyogenes/Streptococcus pneumoniae* | Firmicutes | Bacteria |
| 2.A.1.21.1 | MFS | Q1D3K0 | MXAN_4605 | P95827 | 2.83E-22 | 12 | 12 | 9.95 | Macrolide | Multiple drugs | *Streptococcus pyogenes/Streptococcus pneumoniae* | Firmicutes | Bacteria |
| 2.A.1.21.1 | MFS | Q1D447 | MXAN_4404 | P95827 | 1.86E-20 | 12 | 12 | 11.1 | Macrolide | Multiple drugs | *Streptococcus pyogenes/Streptococcus pneumoniae* | Firmicutes | Bacteria |
| 2.A.1.21.1 | MFS | Q1DGD0 | MXAN_0009 | P95827 | 2.16E-17 | 12 | 12 | 11.9 | Macrolide | Multiple drugs | *Streptococcus pyogenes/Streptococcus pneumoniae* | Firmicutes | Bacteria |
| 2.A.1.21.1 | MFS | Q1D1Q4 | MXAN_5267 | P95827 | 1.70E-40 | 12 | 12 | 12.05 | Macrolide | Multiple drugs | *Streptococcus pyogenes/Streptococcus pneumoniae* | Firmicutes | Bacteria |
| 2.A.1.21.3 | MFS | Q1D5S6 | MXAN_3815 | O31137 | 4.91E-16 | 12 | 12 | 10.6 | Tetracycline resistance | Drugs | *Mycobacterium smegmatis* | Actinobacteria | Bacteria |
| 2.A.1.24.4 | MFS | Q1CWQ3 | MXAN_7057 | Q1CWQ3 | 0.00E+00 | 12 | 11 | 6.6 | MFS permease | Cations | *Saccharomyces cerevisiae* | Ascomycota | Eukarya |
| 2.A.1.25.2 | MFS | Q1D1G6 | MXAN_5357 | P0AE16 | 4.47E-36 | 12 | 14 | 8.55 | Peptides and penicillin | Peptides | *Escherichia coli* | Proteobacteria | Bacteria |
| 2.A.1.25.2 | MFS | Q1D2S9 | MXAN_4887 | P0AE16 | 2.48E-101 | 12 | 14 | 10.5 | Peptides and penicillin | Peptides | *Escherichia coli* | Proteobacteria | Bacteria |
| 2.A.1.25.2 | MFS | Q1DC14 | MXAN_1557 | P0AE16 | 1.53E-44 | 12 | 14 | 11 | Peptides and penicillin | Peptides | *Escherichia coli* | Proteobacteria | Bacteria |
| 2.A.1.36.2 | MFS | Q1DG53 | MXAN_0087 | A8GHT9 | 5.36E-46 | 12 | 12 | 10.7 | Hypothetical MFS | Cations | *Escherichia coli* | Proteobacteria | Bacteria |
| 2.A.1.49.1 | MFS | Q1D0C6 | MXAN_5760 | Q9GQQ0 | 3.66E-59 | 12 | 12 | 9.5 | Unknown | Lipids | *Drosophila melanogaster* | Arthropoda | Eukarya |
| 2.A.1.60.1 | MFS | Q1DA69 | MXAN_2233 | Q07609 | 1.55E-26 | 12 | 12 | 7.1 | Opine | Amino acids & conjugates | *Rhizobium meliloti* | Proteobacteria | Bacteria |
| 2.A.3.3.2 | APC | Q1DDV1 | MXAN_0911 | P18581 | 2.80E-12 | 12 | 14 | 8 | Low affinity basic amino acid | Amino acids & conjugates | *Mus musculus* | Chordata | Eukarya |
| 2.A.3.8.7 | APC | Q1DC13 | MXAN_1558 | Q9R0S5 | 3.02E-42 | 12 | 12 | 10.25 | Amino acids; secondary: Na^+^, Li^+^, H^+^; neurotoxicant | Amino acids & conjugates | *Rattus norvegicus* | Chordata | Eukarya |
| 2.A.4.1.3 | CDF | Q1D6Z4 | MXAN_3382 | O07084 | 8.03E-58 | 7 | 5 | 2.6 | Cd^2+^ or Zn^2+^ :H^+^ and K^+^ | Cations | *Bacillus subtilis* | Firmicutes | Bacteria |
| 2.A.4.1.3 | CDF | Q1D1Q7 | MXAN_5264 | O07084 | 7.51E-46 | 6 | 5 | 4.4 | Cd^2+^ or Zn^2+^ :H+ and K^+^ | Cations | *Bacillus subtilis* | Firmicutes | Bacteria |
| 2.A.4.7.1 | CDF | Q1D7X1 | MXAN_3043 | P69380 | 1.98E-27 | 6 | 6 | 5 | Zn^2+^/Cd^2+^/Hg^2+^/Fe^2+^: H^+^ | Cations | *Escherichia coli* | Proteobacteria | Bacteria |
| 2.A.5.4.9 | ZIP | Q1D0K3 | MXAN_5682 | Q8BZH0 | 6.01E-11 | 8 | 7 | 3.75 | Zn^2+^ | Cations | *Mus musculus* | Chordata | Eukarya |
| 2.A.5.5.1 | ZIP | Q1D508 | MXAN_4087 | P0A8H3 | 1.51E-12 | 8 | 8 | 7.05 | Fe^2+^, Co^2+^, Mn^2+^, Cd^2+^ and Zn^2+^ | Cations | *Escherichia coli* | Proteobacteria | Bacteria |
| 2.A.6.1.2 | RND | Q1DDM9 | MXAN_0983 | P13511 | 1.36E-168 | 8 | 12 | 4.35 | Co^2+^; Zn^2+^; Cd^2+^ | Cations | *Alcaligenes eutrophus* | Proteobacteria | Bacteria |
| 2.A.6.1.3 | RND | Q1DDM4 | MXAN_0988 | Q9ZHC9 | E+000 | 12 | 12 | 8.95 | Ag^+^ | Cations | *Salmonella typhimurium* | Proteobacteria | Bacteria |
| 2.A.6.1.5 | RND | Q1D665 | MXAN_3671 | Q88RT6 | E+000 | 9 | 12 | 4.65 | Zn^2+^, Cd^2+^, Pb^2+^ | Cations | *Pseudomonas putida* | Proteobacteria | Bacteria |
| 2.A.6.1.5 | RND | Q1CVN1 | MXAN_7437 | Q88RT6 | E+000 | 11 | 12 | 4.65 | Zn^2+^, Cd^2+^, Pb^2+^ | Cations | *Pseudomonas putida* | Proteobacteria | Bacteria |
| 2.A.6.1.5 | RND | Q1D6S7 | MXAN_3449 | Q88RT6 | E+000 | 9 | 12 | 5.95 | Zn^2+^, Cd^2+^, Pb^2+^ | Cations | *Pseudomonas putida* | Proteobacteria | Bacteria |
| 2.A.6.2.1 | RND | Q1CW70 | MXAN_7240 | P24181 | E+000 | 12 | 12 | 11 | Multidrug resistance | Multiple drugs | *Escherichia coli* | Proteobacteria | Bacteria |
| 2.A.6.2.2 | RND | Q1D2D6 | MXAN_5032 | P31224 | E+000 | 12 | 12 | 9.7 | Multidrug/dye/detergent/bile salt/organic solvent resistance | Multiple drugs | *Escherichia coli* | Proteobacteria | Bacteria |
| 2.A.6.2.15 | RND | Q1DG48 | MXAN_0092 | Q9HVI9 | E+000 | 14 | 12 | 10.45 | Multidrug | Multiple drugs | *Pseudomonas aeruginosa* | Proteobacteria | Bacteria |
| 2.A.6.2.26 | RND | Q1D4Q0 | MXAN_4198 | Q87SJ8 | 1.82E-14 | 1 | 1 | 0.7 | Multidrug | Multiple drugs | *Vibrio parahaemolyticus* | Proteobacteria | Bacteria |
| 2.A.6.3.1 | RND | Q1D5J0 | MXAN_3903 | P25197 | 3.17E-145 | 12 | 12 | 7.85 | Lipooligosaccharide | Carbohydrates | *Rhizobium meliloti* | Proteobacteria | Bacteria |
| 2.A.6.3.1 | RND | Q1DEI8 | MXAN_0667 | P25197 | 1.82E-171 | 13 | 12 | 7.9 | Lipooligosaccharide | Carbohydrates | *Rhizobium meliloti* | Proteobacteria | Bacteria |
| 2.A.6.3.1 | RND | Q1CZ65 | MXAN_6177 | P25197 | 8.16E-94 | 12 | 12 | 8.2 | Lipooligosaccharide | Carbohydrates | *Rhizobium meliloti* | Proteobacteria | Bacteria |
| 2.A.6.3.1 | RND | Q1D5H0 | MXAN_3923 | P25197 | 1.59E-103 | 12 | 12 | 8.25 | Lipooligosaccharide | Carbohydrates | *Rhizobium meliloti* | Proteobacteria | Bacteria |
| 2.A.6.3.1 | RND | Q1D847 | MXAN_2967 | P25197 | E+000 | 13 | 12 | 9.3 | Lipooligosaccharide | Carbohydrates | *Rhizobium meliloti* | Proteobacteria | Bacteria |
| 2.A.6.3.1 | RND | Q1DEX6 | MXAN_0528 | P25197 | E+000 | 9 | 12 | 9.7 | Lipooligosaccharide | Carbohydrates | *Rhizobium meliloti* | Proteobacteria | Bacteria |
| 2.A.6.4.1 | RND | Q1D3B6 | MXAN_4691 | P0AG90 | 1.13E-73 | 5 | 6 | 1.5 | Proteins | Proteins | *Escherichia coli* | Proteobacteria | Bacteria |
| 2.A.6.7.1 | RND | Q1CY80 | MXAN_6520 | O29039 | 1.16E-12 | 12 | 12 | 2.5 | Unknown | Other hydrophobic substances | *Archaeoglobus fulgidus* | Euryarchaeota | Archaea |
| 2.A.6.7.1 | RND | Q1D5Z2 | MXAN_3749 | O29039 | 2.80E-18 | 9 | 12 | 6.75 | Unknown | Other hydrophobic substances | *Archaeoglobus fulgidus* | Euryarchaeota | Archaea |
| 2.A.6.7.1 | RND | Q1DF63 | MXAN_0440 | O29039 | 3.08E-17 | 9 | 12 | 6.8 | Unknown | Other hydrophobic substances | *Archaeoglobus fulgidus* | Euryarchaeota | Archaea |
| 2.A.6.7.2 | RND | Q1DF62 | MXAN_0441 | Q58957 | 2.72E-17 | 10 | 6 | 2.2 | Unknown | Other hydrophobic substances | *Methanococcus jannaschii* | Euryarchaeota | Archaea |
| 2.A.7.1.4 | DMT | Q1D671 | MXAN_3665 | P69937 | 8.86E-31 | 4 | 4 | 3.75 | Quaternary ammonium | Multiple drugs | *Escherichia coli* | Proteobacteria | Bacteria |
| 2.A.7.1.10 | DMT | Q1DFB7 | MXAN_0384 | D5CES3 | 6.34E-28 | 4 | 4 | 3.65 | SugE Supressor of GroEL/ES | Multiple drugs | *Escherichia coli* | Proteobacteria | Bacteria |
| 2.A.7.1.10 | DMT | Q1D1W2 | MXAN_5209 | D5CES3 | 9.76E-21 | 4 | 4 | 3.7 | SugE Supressor of GroEL/ES | Multiple drugs | *Escherichia coli* | Proteobacteria | Bacteria |
| 2.A.7.1.11 | DMT | Q1D1Y4 | MXAN_5186 | Q2FD83 | 1.44E-28 | 4 | 4 | 3.4 | Cationic lipophilic drugs | Multiple drugs | *Escherichia coli* | Proteobacteria | Bacteria |
| 2.A.7.2.1 | DMT | Q1D126 | MXAN_5504 | P29939 | 2.85E-23 | 5 | 5 | 4.65 | Unknown | Drugs | *Pseudomonas denitrificans* | Proteobacteria | Bacteria |
| 2.A.7.3.4 | DMT | Q1D2H4 | MXAN_4993 | P39649 | 4.75E-06 | 10 | 10 | 8.15 | Unknown | Drugs | *Bacillus subtilis* | Firmicutes | Bacteria |
| 2.A.7.3.6 | DMT | Q1DFY5 | MXAN_0155 | P0AA67 | 3.45E-77 | 10 | 10 | 9.15 | Thronine/Homoserine | Amino acids & conjugates | *Escherichia coli* | Proteobacteria | Bacteria |
| 2.A.7.3.11 | DMT | Q1DA85 | MXAN_2217 | A4FP84 | 0.0001004 | 10 | 10 | 7.95 | Unknown | Unknown | *Escherichia coli* | Proteobacteria | Bacteria |
| 2.A.7.3.11 | DMT | Q1CXM9 | MXAN_6727 | A4FP84 | 0.0001043 | 10 | 10 | 7.15 | Unknown | Unknown | *Escherichia coli* | Proteobacteria | Bacteria |
| 2.A.7.3.13 | DMT | Q1CZU7 | MXAN_5939 | A7JQ96 | 3.45E-05 | 9 | 10 | 7.3 | Unknown | Drugs | *Pelagibacter ubique* | Proteobacteria | Bacteria |
| 2.A.7.23.1 | DMT | Q1DFU1 | MXAN_0200 | P42243 | 3.76E-11 | 8 | 10 | 7 | Tryptophan | Amino acids & conjugates | *Bacillus subtilis* | Firmicutes | Bacteria |
| 2.A.7.24.5 | DMT | Q1D740 | MXAN_3334 | Q4FKW8 | 4.14E-11 | 10 | 10 | 6.5 | Unknown | Drugs | *Pelagibacter ubique* | Proteobacteria | Bacteria |
| 2.A.7.26.1 | DMT | Q1D445 | MXAN_4406 | P76169 | 6.75E-26 | 4 | 4 | 3.7 | Unknown | Drugs | *Escherichia coli* | Proteobacteria | Bacteria |
| 2.A.12.4.1 | AAA | Q1DEX2 | MXAN_0532 | Q6MDZ0 | 5.33E-54 | 12 | 12 | 8.65 | NAD^+^:ADP | Nucleotides | *Protochlamydia amoebophila* | Chlamydiae | Bacteria |
| 2.A.17.1.1 | POT | Q1D947 | MXAN_2607 | P0C2U2 | 3.26E-65 | 12 | 13 | 7.6 | Di- or tripeptide: H^+^ | Peptides | *Lactococcus lactis* | Firmicutes | Bacteria |
| 2.A.17.4.4 | POT | Q1D946 | MXAN_2608 | Q9ES07 | 5.28E-66 | 11 | 11 | 4.45 | Peptides | Peptides | *Mus musculus* | Chordata | Eukarya |
| 2.A.20.1.2 | PiT | Q1DA22 | MXAN_2280 | P43676 | 1.14E-27 | 9 | 10 | 6.2 | Low affinity Pi | Organoanions (noncarboxylic) | *Escherichia coli* | Proteobacteria | Bacteria |
| 2.A.21.5.5 | SSS | Q1CZ52 | MXAN_6190 | Q3ZMH1 | 7.65E-43 | 14 | 13 | 8.7 | Na^+^ | Cations | *Danio rerio* | Chordata | Eukarya |
| 2.A.21.7.2 | SSS | Q1D028 | MXAN_5858 | P32705 | 3.15E-162 | 13 | 14 | 11.25 | Na^+^ | Cations | *Escherichia coli* | Proteobacteria | Bacteria |
| 2.A.21.9.1 | SSS | Q1DFU4 | MXAN_0197 | Q9HV74 | 1.31E-23 | 10 | 13 | 2.75 | Na^+^ | Cations | *Pseudomonas aeruginosa* | Proteobacteria | Bacteria |
| 2.A.21.9.1 | SSS | Q1D034 | MXAN_5852 | Q9HV74 | E+000 | 13 | 13 | 11.95 | Na^+^ | Cations | *Pseudomonas aeruginosa* | Proteobacteria | Bacteria |
| 2.A.23.1.5 | DAACS | Q1D5L6 | MXAN_3877 | O59010 | 7.71E-69 | 10 | 9 | 5.95 | Archaeal aspartate, Na^+^ | Amino acids & conjugates | *Pyrococcus horikoshii* | Euryarchaeota | Archaea |
| 2.A.23.1.5 | DAACS | Q1D9Q0 | MXAN_2403 | O59010 | 7.90E-61 | 10 | 9 | 6.45 | Archaeal aspartate, Na^+^ | Amino acids & conjugates | *Pyrococcus horikoshii* | Euryarchaeota | Archaea |
| 2.A.23.1.6 | DAACS | Q1D844 | MXAN_2970 | P96603 | 6.95E-132 | 8 | 10 | 6.95 | Succinate, fumurate, malate and oxaloacetate; H^+^ | Di- & tricarboxylates | *Bacillus subtilis* | Firmicutes | Bacteria |
| 2.A.28.1.2 | BASS | Q1D5R2 | MXAN_3829 | Q12908 | 5.98E-18 | 9 | 9 | 8.1 | Ileal bile acid:Na^+^ | Drugs | *Homo sapiens* | Chordata | Eukarya |
| 2.A.28.1.3 | BASS | Q1CXF7 | MXAN_6799 | Q70EX6 | 7.98E-05 | 8 | 9 | 6.9 | The organic anion:Na^+^ | Anions | *Rattus norvegicus* | Chordata | Eukarya |
| 2.A.33.1.1 | NhaA | Q1CZI3 | MXAN_6055 | P13738 | 6.52E-78 | 12 | 10 | 7.95 | Na^+^:2H^+^ | Cations | *Escherichia coli* | Proteobacteria | Bacteria |
| 2.A.33.1.2 | NhaA | Q1D5J5 | MXAN_3898 | Q56725 | 8.17E-79 | 10 | 10 | 5.4 | Na^+^,K^+^:H^+^ | Cations | *Vibrio parahaemolyticus* | Proteobacteria | Bacteria |
| 2.A.36.6.1 | CPA1 | Q1D257 | MXAN_5112 | O29412 | 2.55E-77 | 13 | 13 | 8.75 | Na^+^:H^+^ | Cations | *Archaeoglobus fulgidus* | Euryarchaeota | Archaea |
| 2.A.37.1.2 | CPA2 | Q1CX04 | MXAN_6954 | P45522 | 1.82E-140 | 12 | 13 | 11 | K^+^ | Cations | *Escherichia coli* | Proteobacteria | Bacteria |
| 2.A.37.1.3 | CPA2 | Q1CZY3 | MXAN_5903 | Q0ZAH7 | 4.11E-06 | 2 | 13 | 1.3 | K^+^ | Cations | *Alkalimonas amylolytica* | Proteobacteria | Bacteria |
| 2.A.37.1.3 | CPA2 | Q1DA42 | MXAN_2260 | Q0ZAH7 | 1.13E-46 | 13 | 13 | 8.85 | K^+^ | Cations | *Alkalimonas amylolytica* | Proteobacteria | Bacteria |
| 2.A.37.2.3 | CPA2 | Q1CXH1 | MXAN_6785 | Q45308 | 6.96E-34 | 17 | 13 | 10.9 | Spore germination protein | Cations | *Bacillus megaterium* | Firmicutes | Bacteria |
| 2.A.37.4.2 | CPA2 | Q1DFN2 | MXAN_0260 | Q9SUQ7 | 8.89E-98 | 13 | 13 | 10.2 | K^+^:H^+^ | Cations | *Arabidopsis thaliana* | Angiosperms | Eukarya |
| 2.A.45.3.1 | ArsB | Q1D5Q7 | MXAN_3834 | Q10SY9 | 1.80E-41 | 11 | 10 | 6.15 | Silicon | Other hydrophobic substances | *Oryza sativa subsp. japonica* | Angiosperms | Eukarya |
| 2.A.47.4.1 | DASS | Q1D803 | MXAN_3011 | P72958 | 8.64E-76 | 10 | 10 | 8.6 | Sulfur-deprivation response protein; divalent anion, Na^+^, K^+^ | Cations | *Synechocystis sp.* | Cyanobacteria | Bacteria |
| 2.A.47.4.1 | DASS | Q1DCV1 | MXAN_1265 | P72958 | 3.26E-81 | 11 | 10 | 9.35 | Sulfur-deprivation response protein; divalent anion, Na^+^, K^+^ | Cations | *Synechocystis sp.* | Cyanobacteria | Bacteria |
| 2.A.47.5.1 | DASS | Q1D4L1 | MXAN_4237 | Q58086 | 5.34E-40 | 11 | 13 | 8.5 | Na^+^ | Cations | *Methanococcus jannaschii* | Euryarchaeota | Archaea |
| 2.A.49.4.1 | CIC | Q1DDN4 | MXAN_0978 | Q57753 | 3.52E-13 | 10 | 10 | 5.3 | Cl^-^ | Anions | *Methanococcus jannaschii* | Euryarchaeota | Archaea |
| 2.A.49.4.1 | CIC | Q1DG09 | MXAN_0131 | Q57753 | 6.87E-20 | 8 | 10 | 6.45 | Cl^-^ | Anions | *Methanococcus jannaschii* | Euryarchaeota | Archaea |
| 2.A.49.4.1 | CIC | Q1CYU1 | MXAN_6307 | Q57753 | 1.12E-25 | 12 | 10 | 6.85 | Cl^-^ | Anions | *Methanococcus jannaschii* | Euryarchaeota | Archaea |
| 2.A.49.6.1 | CIC | Q1CVK4 | MXAN_7465 | P74477 | 7.28E-26 | 10 | 11 | 8.25 | Cl^-^ | Anions | *Synechocystis sp.* | Cyanobacteria | Bacteria |
| 2.A.50.2.1 | GUP | Q1CY97 | MXAN_6503 | P39580 | 7.58E-28 | 9 | 12 | 4.2 | Alanyl teichoic acid synthesis; glycerol | Sugars & polyols | *Bacillus subtilis* | Firmicutes | Bacteria |
| 2.A.51.1.2 | CHR | Q1CYQ2 | MXAN_6347 | Q55027 | 3.14E-45 | 11 | 12 | 9.55 | Chromate-sensitivity anion permease | Anions | *Synechococcus sp.* | Cyanobacteria | Bacteria |
| 2.A.53.4.1 | SulP | Q1CY95 | MXAN_6505 | P72770 | 1.48E-89 | 12 | 13 | 9.65 | Sulfate | Anions | *Synechocystis sp.* | Cyanobacteria | Bacteria |
| 2.A.53.4.1 | SulP | Q1CY94 | MXAN_6506 | P72770 | 8.84E-78 | 12 | 13 | 10.25 | Sulfate | Anions | *Synechocystis sp.* | Cyanobacteria | Bacteria |
| 2.A.53.3.8 | SulP | Q1D327 | MXAN_4784 | Q8F8H7 | 1.26E-55 | 9 | 10 | 4.05 | Probable bicarbonate | Anions | *Leptospira interrogans* | Spirochaetes | Bacteria |
| 2.A.63.1.3 | CPA3 | Q1D5N0 | MXAN_3863 | P60694 | 1.62E-07 | 3 | 3 | 2.8 | Na^+^:H^+^ | Cations | *Staphylococcus aureus* | Firmicutes | Bacteria |
| 2.A.63.1.4 | CPA3 | Q1D5N3 | MXAN_3860 | O05260 | 6.73E-20 | 3 | 3 | 2.7 | Na^+^:H^+^ | Cations | *Bacillus subtilis* | Firmicutes | Bacteria |
| 2.A.63.1.4 | CPA3 | Q1D5N1 | MXAN_3862 | Q7WY60 | 9.73E-18 | 3 | 3 | 2.7 | Na^+^:H^+^ | Cations | *Bacillus subtilis* | Firmicutes | Bacteria |
| 2.A.63.1.2 | CPA3 | Q1D5M9 | MXAN_3864 | Q9KDA1 | 1.32E-16 | 3 | 3 | 2.75 | Na^+^:H^+^ | Cations | *Bacillus subtilis* | Firmicutes | Bacteria |
| 2.A.63.1.2 | CPA3 | Q1D5N2 | MXAN_3861 | Q9KD98 | 2.18E-89 | 15 | 14 | 12.35 | Na^+^:H^+^ | Cations | *Bacillus subtilis* | Firmicutes | Bacteria |
| 2.A.64.1.1 | Tat | Q1CZY2 | MXAN_5904 | P69423 | 9.76E-21 | 5 | 5 | 3.05 | Unknown | Unknown | *Escherichia coli* | Proteobacteria | Bacteria |
| 2.A.64.2.1 | Tat | Q1CZY1 | MXAN_5905 | Q9XH75 | 9.16E-08 | 1 | 1 | 0.8 | Unknown | Unknown | *Arabidopsis thaliana* | Angiosperms | Eukarya |
| 2.A.66.1.22 | MOP | Q1CWJ1 | MXAN_7119 | D5CJ69 | 1.20E-21 | 12 | 12 | 8.45 | Quinolone:H^+^ | Multiple drugs | *Staphylococcus aureus* | Firmicutes | Bacteria |
| 2.A.66.1.22 | MOP | Q1CXF2 | MXAN_6804 | D5CJ69 | 1.27E-10 | 12 | 12 | 12.5 | Quinolone:H^+^ | Multiple drugs | *Staphylococcus aureus* | Firmicutes | Bacteria |
| 2.A.66.1.22 | MOP | Q1D8L7 | MXAN_2789 | D5CJ69 | 2.88E-21 | 12 | 12 | 8.65 | Quinolone:H^+^ | Multiple drugs | *Burkholderia vietnamiensis* | Proteobacteria | Bacteria |
| 2.A.66.1.9 | MOP | Q1CWT8 | MXAN_7022 | Q9F5N7 | 4.48E-51 | 12 | 12 | 11.35 | Norfloxacin, polymyxin B resistance | Multiple drugs | *Burkholderia vietnamiensis* | Proteobacteria | Bacteria |
| 2.A.66.2.6 | MOP | Q1DDH7 | MXAN_1035 | O32273 | 2.09E-15 | 14 | 14 | 11.85 | Teichuronic acid (polysaccharide) | Carbohydrates | *Bacillus subtilis* | Firmicutes | Bacteria |
| 2.A.66.2.9 | MOP | Q1CVQ1 | MXAN_7416 | O26442 | 2.07E-13 | 14 | 14 | 11.55 | Succinoglycan Biosynthesis homologue (polysaccharide) | Carbohydrates | *Methanothermobacter thermautotrophicus* | Euryarchaeota | Archaea |
| 2.A.66.4.1 | MOP | Q1D6H2 | MXAN_3558 | P37169 | 1.56E-44 | 14 | 14 | 9.9 | Mouse virulence factor MviN, a putative lipid flippase | Drugs | *Salmonella typhimurium* | Proteobacteria | Bacteria |
| 2.A.67.2.6 | OPT | Q1D9V0 | MXAN_2353 | Q54EF1 | 5.82E-07 | 17 | 13 | 7.2 | Unknown | Unknown | *Dictyostelium discoideum* | Mycetozoa | Eukarya |
| 2.A.67.3.1 | OPT | Q1D9P8 | MXAN_2405 | Q9S433 | 2.45E-54 | 16 | 15 | 10.65 | Early sporulation protein, oligopeptide | Peptides | *Myxococcus xanthus* | Proteobacteria | Bacteria |
| 2.A.67.3.1 | OPT | Q1D7S9 | MXAN_3091 | Q9S433 | 5.60E-47 | 15 | 15 | 10.65 | Early sporulation protein, oligopeptide | Peptides | *Myxococcus xanthus* | Proteobacteria | Bacteria |
| 2.A.67.3.1 | OPT | Q1DDT0 | MXAN_0932 | Q9S433 | E+000 | 15 | 15 | 16.75 | Early sporulation protein, oligopeptide | Peptides | *Myxococcus xanthus* | Proteobacteria | Bacteria |
| 2.A.67.4.2 | OPT | Q1D720 | MXAN_3356 | O58099 | 5.45E-71 | 18 | 17 | 10 | Uncharacterized oligopeptide | Peptides | *Pyrococcus horikoshii* | Euryarchaeota | Archaea |
| 2.A.69.3.1 | AEC | Q1DE54 | MXAN_0806 | Q48797 | 5.50E-07 | 10 | 10 | 7.75 | Malate permease | Anions | *Oenococcus oeni* | Firmicutes | Bacteria |
| 2.A.72.1.1 | KUP | Q1DFF8 | MXAN_0341 | P63183 | 2.44E-177 | 12 | 13 | 10.6 | K^+^ | Cations | *Escherichia coli* | Proteobacteria | Bacteria |
| 2.A.73.1.1 | AtoE | Q1DEF1 | MXAN_0704 | P76460 | 5.34E-87 | 11 | 11 | 9.2 | Short chain fatty acid | Lipids | *Escherichia coli* | Proteobacteria | Bacteria |
| 2.A.76.1.5 | RhtB | Q1D2A1 | MXAN_5068 | P76249 | 1.08E-23 | 5 | 6 | 4.4 | Leucine | Amino acids & conjugates | *Escherichia coli* | Proteobacteria | Bacteria |
| 2.A.78.2.1 | LIV-E | Q1D212 | MXAN_5158 | A4EZB2 | 1.40E-08 | 5 | 6 | 4.1 | Branched chain amino acid | Amino acids & conjugates | *Bacillus subtilis* | Firmicutes | Bacteria |
| 2.A.79.1.1 | ThrE | Q1DBY8 | MXAN_1583 | Q79VD1 | 1.14E-09 | 10 | 11 | 5.8 | Threonine/Serine | Amino acids & conjugates | *Corynebacterium glutamicum* | Actinobacteria | Bacteria |
| 2.A.85.1.1 | ArAE | Q1DCH0 | MXAN_1396 | P75870 | 6.40E-20 | 8 | 12 | 7.25 | Unknown | Unknown | *Escherichia coli* | Proteobacteria | Bacteria |
| 2.A.85.1.1 | ArAE | Q1DEF0 | MXAN_0705 | P75870 | 1.94E-18 | 12 | 12 | 9.55 | Unknown | Unknown | *Escherichia coli* | Proteobacteria | Bacteria |
| 2.A.86.1.5 | AI-2E | Q1D6E5 | MXAN_3588 | C9VRY8 | 3.42E-35 | 8 | 7 | 7.25 | Unknown | Unknown | *Brucella abortus* | Proteobacteria | Bacteria |
| 2.A.86.1.5 | AI-2E | Q1D502 | MXAN_4093 | C9VRY8 | 3.52E-12 | 8 | 7 | 7.3 | Unknown | Unknown | *Brucella abortus* | Proteobacteria | Bacteria |
| 2.A.86.1.7 | AI-2E | Q1CW40 | MXAN_7270 | O53656 | 1.63E-15 | 8 | 7 | 6.3 | Unknown | Unknown | *Escherichia coli* | Proteobacteria | Bacteria |
| 2.A.86.1.8 | AI-2E | Q1D0Q0 | MXAN_5634 | O32095 | 2.96E-16 | 8 | 7 | 4.9 | Unknown | Unknown | *Escherichia coli* | Proteobacteria | Bacteria |
| 2.A.86.1.8 | AI-2E | Q1DFH3 | MXAN_0325 | O32095 | 2.54E-14 | 7 | 7 | 5.55 | Unknown | Unknown | *Escherichia coli* | Proteobacteria | Bacteria |
| 2.A.86.1.8 | AI-2E | Q1D8D7 | MXAN_2875 | O32095 | 1.32E-16 | 8 | 7 | 5.8 | Unknown | Unknown | *Escherichia coli* | Proteobacteria | Bacteria |
| 2.A.86.1.8 | AI-2E | Q1DBZ0 | MXAN_1581 | O32095 | 8.52E-06 | 7 | 7 | 6.95 | Unknown | Unknown | *Escherichia coli* | Proteobacteria | Bacteria |
| 2.A.88.2.1 | VUT | Q1DA94 | MXAN_2207 | Q2KUS5 | 8.69E-18 | 6 | 5 | 4.65 | Uncharacterized vitamin | Vitamins & vitamin or cofactor precursors | *Bordetella avium* | Proteobacteria | Bacteria |
| 2.A.93.1.4 | UBS1 | Q1D5S4 | MXAN_3817 | P39836 | 1.71E-53 | 9 | 10 | 7.4 | Na^+^ | Cations | *Escherichia coli* | Proteobacteria | Bacteria |
| 2.A.95.1.4 | NAAT | Q1D391 | MXAN_4720 | Q8J305 | 7.08E-31 | 6 | 6 | 5.15 | Glycine, alanine and neutral L-amino acids | Amino acids & conjugates | *Thermococcus sp.* | Euryarchaeota | Archaea |
| 2.A.98.1.3 | PSE | Q1D4D6 | MXAN_4312 | P62723 | 2.99E-34 | 11 | 10 | 7.7 | Unknown | Unknown | *Escherichia coli* | Proteobacteria | Bacteria |
| 2.A.102.3.1 | TSUP | Q1DDA5 | MXAN_1108 | P0AD30 | 8.04E-35 | 9 | 9 | 6.35 | Unknown | Unknown | *Escherichia coli* | Proteobacteria | Bacteria |
| 2.A.102.4.1 | TSUP | Q1CZ80 | MXAN_6162 | Q9UYH7 | 5.11E-14 | 7 | 7 | 4.3 | Unknown | Unknown | *Pyrococcus abyssi* | Euryarchaeota | Archaea |
| 2.A.102.4.1 | TSUP | Q1D329 | MXAN_4782 | Q9UYH7 | 9.40E-16 | 8 | 7 | 4.75 | Unknown | Unknown | *Pyrococcus abyssi* | Euryarchaeota | Archaea |
| 2.A.102.4.1 | TSUP | Q1DFW3 | MXAN_0177 | Q9UYH7 | 1.13E-15 | 8 | 7 | 4.9 | Unknown | Unknown | *Pyrococcus abyssi* | Euryarchaeota | Archaea |
| 2.A.102.4.1 | TSUP | Q1D2K6 | MXAN_4961 | Q9UYH7 | 1.19E-21 | 7 | 7 | 5.55 | Unknown | Unknown | *Pyrococcus abyssi* | Euryarchaeota | Archaea |
| 2.A.102.4.2 | TSUP | Q1D4C7 | MXAN_4321 | E7BBJ3 | 8.27E-14 | 8 | 8 | 6.1 | Possible  organo-sulfur-containing compound | Organoanions (noncarboxylic) | *Serratia sp.* | Proteobacteria | Bacteria |
| 2.A.103.1.3 | MPE | Q1D0S9 | MXAN_5605 | P07373 | 3.24E-75 | 10 | 10 | 9.4 | Sporulation protein; peptidoglycan biosynthetic process | Carbohydrates | *Bacillus subtilis* | Firmicutes | Bacteria |
| 2.C.1.1.1 | TonB | Q1DCB7 | MXAN_1449 | P02929 | 2.45E-06 | 2 | 1 | 0.7 | Iron ion, protein, cobalamin, and bacteriocin | Cations | *Escherichia coli* | Proteobacteria | Bacteria |
| 2.C.1.2.1 | TonB | Q1D0D0 | MXAN_5756 | P0A855 | 4.95E-33 | 1 | 1 | 0.85 | Bacteriocin and protein transfer | Toxins | *Escherichia coli* | Proteobacteria | Bacteria |
| 3.A.1.1.6 | ABC | Q1D1E4 | MXAN_5379 | Q48397 | 4.44E-49 | 6 | 6 | 3.65 | Cyclodextrin porter | Carbohydrates | *Klebsiella oxytoca* | Proteobacteria | Bacteria |
| 3.A.1.1.7 | ABC | Q1CW15 | MXAN_7295 | O51923 | 5.92E-60 | 1 | 1 | 0.65 | Maltose/trehalose porter | Sugars & polyols | *Thermococcus litoralis, and Pyrococcus furiosus* | Euryarchaeota | Archaea |
| 3.A.1.1.22 | ABC | Q1D1E5 | MXAN_5378 | Q9X0T0 | 2.34E-48 | 7 | 8 | 4.95 | The maltose, maltotriose, mannotetraose/maltose, maltotriose, trehalose | Sugars & polyols | *Thermotoga maritima* | Thermotogae | Bacteria |
| 3.A.1.1.25 | ABC | Q1CW16 | MXAN_7294 | Q72H67 | 5.92E-66 | 6 | 6 | 5.1 | Trehalose/maltose/sucrose/palatinose | Sugars & polyols | *Thermus thermophilus* | Deinococcus | Bacteria |
| 3.A.1.1.35 | ABC | Q1CW17 | MXAN_7293 | O50453 | 1.65E-57 | 6 | 6 | 5.9 | Trehalose-recycling ABC | Sugars & polyols | *Erwinia rhapontici* | Proteobacteria | Bacteria |
| 3.A.1.2.10 | ABC | Q1D0H2 | MXAN_5713 | O83342 | 4.27E-37 | 9 | 9 | 7.75 | Purine nucleoside permease | Nucleotides | *Treponema pallidum* | Spirochaetes | Bacteria |
| 3.A.1.2.17 | ABC | Q1D0H1 | MXAN_5714 | A2RKA5 | 1.96E-47 | 9 | 9 | 6.4 | General nucleoside | Nucleosides | *Lactococcus lactis subsp. cremoris* | Firmicutes | Bacteria |
| 3.A.1.3.17 | ABC | Q1D127 | MXAN_5503 | Q8YSA2 | 2.09E-47 | 4 | 6 | 2.65 | Basic amino acid | Amino acids & conjugates | *Anabaena sp.* | Cyanobacteria | Bacteria |
| 3.A.1.4.1 | ABC | Q1CXU3 | MXAN_6663 | P22729 | 1.10E-35 | 8 | 10 | 3.2 | Leucine; leucine/isoleucine/valine | Amino acids & conjugates | *Escherichia coli* | Proteobacteria | Bacteria |
| 3.A.1.4.1 | ABC | Q1CXU2 | MXAN_6664 | P0AEX7 | 5.03E-53 | 9 | 9 | 8.25 | Leucine; leucine/isoleucine/valine | Amino acids & conjugates | *Escherichia coli* | Proteobacteria | Bacteria |
| 3.A.1.5.16 | ABC | Q1CY49 | MXAN_6552 | Q9WXN7 | 5.61E-69 | 7 | 6 | 5.6 | Probable β-glucoside | Enzymes | *Thermotoga maritima* | Thermotogae | Bacteria |
| 3.A.1.5.19 | ABC | Q1DAS4 | MXAN_2020 | Q2FZR6 | 2.29E-14 | 6 | 6 | 3.55 | Major oligopeptide | Peptides | *Staphylococcus aureus* | Firmicutes | Bacteria |
| 3.A.1.5.25 | ABC | Q1DAS5 | MXAN_2019 | Q9CIL1 | 7.41E-14 | 6 | 6 | 4 | ABC peptide/signalling peptide | Peptides | *Lactococcus lactis subsp. lactis* | Firmicutes | Bacteria |
| 3.A.1.6.1 | ABC | Q1D2Z2 | MXAN_4820 | P16701 | 1.46E-69 | 6 | 6 | 5.85 | Sulfate/thiosulfate | Anions | *Escherichia coli* | Proteobacteria | Bacteria |
| 3.A.1.6.1 | ABC | Q1D2Z3 | MXAN_4819 | P0AEB0 | 4.00E-77 | 6 | 6 | 5.9 | Sulfate/thiosulfate | Anions | *Escherichia coli* | Proteobacteria | Bacteria |
| 3.A.1.7.1 | ABC | Q1D322 | MXAN_4789 | P0AGH8 | 1.69E-27 | 6 | 6 | 2.75 | Phosphate | Anions | *Escherichia coli* | Proteobacteria | Bacteria |
| 3.A.1.7.2 | ABC | Q1D321 | MXAN_4790 | Q7WTY6 | 9.59E-25 | 6 | 6 | 5 | Phosphate | Anions | *Mycobacterium smegmatis* | Actinobacteria | Bacteria |
| 3.A.1.8.1 | ABC | Q1CXW1 | MXAN_6645 | P37329 | 4.41E-39 | 1 | 1 | 0.75 | Molybdate | Anions | *Escherichia coli* | Proteobacteria | Bacteria |
| 3.A.1.8.2 | ABC | Q1CXW2 | MXAN_6644 | Q8TJ86 | 4.33E-39 | 5 | 5 | 4.35 | Molybdate/tungstate | Anions | *Methanosarcina acetivorans* | Euryarchaeota | Archaea |
| 3.A.1.10.3 | ABC | Q1DE86 | MXAN_0771 | P71338 | 8.23E-65 | 12 | 12 | 7.3 | Fe^3+^ | Cations | *Haemophilus influenzae* | Proteobacteria | Bacteria |
| 3.A.1.10.3 | ABC | Q1CWJ5 | MXAN_7115 | P71338 | 1.68E-19 | 12 | 12 | 8.45 | Fe^3+^ | Cations | *Haemophilus influenzae* | Proteobacteria | Bacteria |
| 3.A.1.12.2 | ABC | Q1DA52 | MXAN_2250 | P46921 | 1.09E-76 | 7 | 8 | 6.6 | Glycine betaine | Amino acids & conjugates | *Bacillus subtilis* | Firmicutes | Bacteria |
| 3.A.1.12.4 | ABC | Q1DEQ8 | MXAN_0597 | O34878 | 1.60E-37 | 4 | 5 | 4 | Uptake system for choline, L-carnitine, D-carnitine, glycine betaine, proline betaine, crotonobetaine, γ-butyrobetaine, dimethylsulfonioacetate, dimethylsulfoniopropionate, ectoine and choline-O-sulfate | Amino acids & conjugates | *Bacillus subtilis* | Firmicutes | Bacteria |
| 3.A.1.14.1 | ABC | Q1DEG9 | MXAN_0686 | P15029 | 3.87E-23 | 10 | 10 | 8.25 | Fe^3+^ or ferric-dicitrate | Cations | *Escherichia coli* | Proteobacteria | Bacteria |
| 3.A.1.14.10 | ABC | Q1DEH0 | MXAN_0685 | Q99YA3 | 7.21E-11 | 9 | 9 | 6.3 | Heme | Cofactors | *Streptococcus pyogenes* | Firmicutes | Bacteria |
| 3.A.1.14.17 | ABC | Q1CY26 | MXAN_6575 | A8GDS7 | 4.54E-46 | 9 | 9 | 6.2 | Heme | Cofactors | *Serratia proteamaculans* | Proteobacteria | Bacteria |
| 3.A.1.14.19 | ABC | Q1CY25 | MXAN_6576 | Q32AX9 | 3.83E-20 | 1 | 2 | 0.7 | Heme | Cofactors | *Shigella dysenteriae serotype 1* | Proteobacteria | Bacteria |
| 3.A.1.14.20 | ABC | Q1DCP6 | MXAN_1320 | Q93SS2 | 1.21E-59 | 8 | 9 | 5.9 | Heme | Cofactors | *Plesiomonas shigelloides* | Proteobacteria | Bacteria |
| 3.A.1.15.5 | ABC | Q1D865 | MXAN_2948 | P39832 | 5.00E-11 | 9 | 7 | 5.15 | Zn^2+^ | Cations | *Escherichia coli* | Proteobacteria | Bacteria |
| 3.A.1.17.2 | ABC | Q1DGA4 | MXAN_0036 | O85765 | 1.53E-20 | 6 | 6 | 5.05 | Aromatic sulfonate | Aromatic compounds | *Pseudomonas putida* | Proteobacteria | Bacteria |
| 3.A.1.17.6 | ABC | Q1D8I0 | MXAN_2832 | Q9CLG9 | 3.70E-29 | 8 | 6 | 5.4 | Putative hydroxymethylpyrimidine transport system; takes up a thiamin salvage pathway intermediate | Aromatic compounds | *Pasteurella multocida* | Proteobacteria | Bacteria |
| 3.A.1.21.1 | ABC | Q1D1P5 | MXAN_5276 | Q9Z375 | 3.22E-91 | 6 | 7 | 5 | Fe^3+^-Yersiniabactin | Siderophores; siderophores-Fe complexes | *Yersinia pestis* | Proteobacteria | Bacteria |
| 3.A.1.21.1 | ABC | Q1D1P6 | MXAN_5275 | Q9R7V3 | 2.52E-97 | 6 | 7 | 5.4 | Fe^3+^-Yersiniabactin | Siderophores; siderophores-Fe complexes | *Yersinia pestis* | Proteobacteria | Bacteria |
| 3.A.1.24.2 | ABC | Q1DDP5 | MXAN_0967 | O32168 | 2.49E-37 | 5 | 5 | 5.2 | L- and D-methionine | Amino acids & conjugates | *Bacillus subtilis* | Firmicutes | Bacteria |
| 3.A.1.27.2 | ABC | Q1D5I3 | MXAN_3910 | Q8L4R0 | 9.01E-25 | 4 | 5 | 4.25 | Chloroplast lipid | Lipids | *Arabidopsis thaliana* | Angiosperms | Eukarya |
| 3.A.1.27.2 | ABC | Q1D5I4 | MXAN_3909 | Q8L4R0 | 5.35E-23 | 5 | 5 | 4.45 | Chloroplast lipid | Lipids | *Arabidopsis thaliana* | Angiosperms | Eukarya |
| 3.A.1.27.2 | ABC | Q1D620 | MXAN_3717 | Q8L4R0 | 2.14E-38 | 6 | 5 | 4.95 | Chloroplast lipid | Lipids | *Arabidopsis thaliana* | Angiosperms | Eukarya |
| 3.A.1.27.2 | ABC | Q1DFP3 | MXAN_0249 | Q8L4R0 | 1.15E-43 | 5 | 5 | 5.1 | Chloroplast lipid | Lipids | *Arabidopsis thaliana* | Angiosperms | Eukarya |
| 3.A.1.27.3 | ABC | Q1D5I1 | MXAN_3912 | P64604 | 4.38E-05 | 1 | 1 | 0.95 | Phospholipid | Lipids | *Escherichia coli* | Proteobacteria | Bacteria |
| 3.A.1.27.3 | ABC | Q1DFP1 | MXAN_0251 | P64604 | 2.70E-05 | 1 | 1 | 1 | Phospholipid | Lipids | *Escherichia coli* | Proteobacteria | Bacteria |
| 3.A.1.27.4 | ABC | Q1D618 | MXAN_3719 | Q0S7K1 | 1.94E-06 | 1 | 1 | 0.85 | Cholesterol | Lipids | *Rhodococcus sp.* | Actinobacteria | Bacteria |
| 3.A.1.103.1 | ABC | Q1D3I2 | MXAN_4623 | Q48475 | 4.12E-34 | 7 | 6 | 6.1 | Lipopolysaccharide | Carbohydrates | *Klebsiella pneumoniae* | Proteobacteria | Bacteria |
| 3.A.1.105.3 | ABC | Q1D1K6 | MXAN_5316 | Q70J76 | 4.63E-14 | 6 | 6 | 5.55 | Biosynthetic precursor of chromomycin | Specific drugs | *Streptomyces griseus* | Actinobacteria | Bacteria |
| 3.A.1.106.1 | ABC | Q1D395 | MXAN_4716 | P60752 | 2.76E-80 | 4 | 5 | 2.7 | Phospholipid, LPS, lipid A and drug (flippase) | Lipids | *Escherichia coli* | Proteobacteria | Bacteria |
| 3.A.1.106.1 | ABC | Q1DEA9 | MXAN_0748 | P60752 | 6.52E-81 | 4 | 5 | 3.25 | Phospholipid, LPS, lipid A and drug (flippase) | Lipids | *Escherichia coli* | Proteobacteria | Bacteria |
| 3.A.1.106.2 | ABC | Q1DCT0 | MXAN_1286 | Q2G2M9 | 6.66E-71 | 6 | 5 | 3.9 | Homodimeric Sav1866 multidrug | Multiple drugs | *Staphylococcus aureus* | Firmicutes | Bacteria |
| 3.A.1.107.1 | ABC | Q1D7B7 | MXAN_3257 | P30964 | 4.58E-12 | 6 | 6 | 6.1 | Putative heme | Cofactors | *Bradyrhizobium japonicum* | Proteobacteria | Bacteria |
| 3.A.1.109.1 | ABC | Q1DBD6 | MXAN_1789 | P08716 | 1.29E-33 | 6 | 8 | 4 | α-Hemolysin | Toxins | *Escherichia coli* | Proteobacteria | Bacteria |
| 3.A.1.110.1 | ABC | Q1D8F9 | MXAN_2853 | Q93GK5 | 2.09E-105 | 4 | 6 | 3.3 | Microcin E492 | Peptides | *Klebsiella pneumoniae* | Proteobacteria | Bacteria |
| 3.A.1.111.2 | ABC | Q1CX25 | MXAN_6934 | P33116 | 6.61E-85 | 5 | 5 | 3.6 | Subtilin | Peptides | *Bacillus subtilis* | Firmicutes | Bacteria |
| 3.A.1.115.1 | ABC | Q1CXD0 | MXAN_6826 | P46904 | 5.97E-32 | 5 | 6 | 5 | Na^+^ | Cations | *Bacillus subtilis* | Firmicutes | Bacteria |
| 3.A.1.122.1 | ABC | Q1D4P8 | MXAN_4200 | P75830 | 3.28E-20 | 1 | 1 | 0.6 | Macrolide | Specific drugs | *Escherichia coli* | Proteobacteria | Bacteria |
| 3.A.1.122.1 | ABC | Q1D4S3 | MXAN_4175 | P75830 | 1.09E-27 | 1 | 1 | 0.85 | Macrolide | Specific drugs | *Escherichia coli* | Proteobacteria | Bacteria |
| 3.A.1.122.1 | ABC | Q1D4S6 | MXAN_4172 | P75831 | 5.17E-41 | 5 | 4 | 2.15 | Macrolide | Specific drugs | *Escherichia coli* | Proteobacteria | Bacteria |
| 3.A.1.122.1 | ABC | Q1CWG4 | MXAN_7146 | P75831 | 9.98E-16 | 8 | 4 | 2.95 | Macrolide | Specific drugs | *Escherichia coli* | Proteobacteria | Bacteria |
| 3.A.1.122.1 | ABC | Q1DBM7 | MXAN_1695 | P75831 | 3.41E-15 | 8 | 4 | 3.15 | Macrolide | Specific drugs | *Escherichia coli* | Proteobacteria | Bacteria |
| 3.A.1.122.1 | ABC | Q1D4P7 | MXAN_4201 | P75831 | 1.11E-62 | 4 | 4 | 3.2 | Macrolide | Specific drugs | *Escherichia coli* | Proteobacteria | Bacteria |
| 3.A.1.122.1 | ABC | Q1D686 | MXAN_3650 | P75831 | 2.31E-16 | 8 | 4 | 3.45 | Macrolide | Specific drugs | *Escherichia coli* | Proteobacteria | Bacteria |
| 3.A.1.122.1 | ABC | Q1D688 | MXAN_3648 | P75831 | 1.53E-16 | 8 | 4 | 3.65 | Macrolide | Specific drugs | *Escherichia coli* | Proteobacteria | Bacteria |
| 3.A.1.122.1 | ABC | Q1D5W8 | MXAN_3773 | P75831 | 4.69E-17 | 8 | 4 | 3.7 | Macrolide | Specific drugs | *Escherichia coli* | Proteobacteria | Bacteria |
| 3.A.1.122.1 | ABC | Q1D8M3 | MXAN_2783 | P75831 | 3.42E-15 | 8 | 4 | 3.7 | Macrolide | Specific drugs | *Escherichia coli* | Proteobacteria | Bacteria |
| 3.A.1.122.2 | ABC | Q1CWG3 | MXAN_7147 | O31710 | 0.0002559 | 1 | 1 | 0.75 | Antimicrobial peptide resistance | Peptides | *Bacillus subtilis* | Firmicutes | Bacteria |
| 3.A.1.122.2 | ABC | Q1DA34 | MXAN_2268 | O31712 | 7.91E-13 | 7 | 4 | 2.4 | Antimicrobial peptide resistance | Peptides | *Bacillus subtilis* | Firmicutes | Bacteria |
| 3.A.1.122.2 | ABC | Q1D202 | MXAN_5168 | O31712 | 8.49E-18 | 8 | 4 | 3.45 | Antimicrobial peptide resistance | Peptides | *Bacillus subtilis* | Firmicutes | Bacteria |
| 3.A.1.122.2 | ABC | Q1D4S5 | MXAN_4173 | O31712 | 4.90E-42 | 4 | 4 | 3.5 | Antimicrobial peptide resistance | Peptides | *Bacillus subtilis* | Firmicutes | Bacteria |
| 3.A.1.122.2 | ABC | Q1DG33 | MXAN_0107 | O31712 | 8.13E-37 | 4 | 4 | 3.5 | Antimicrobial peptide resistance | Peptides | *Bacillus subtilis* | Firmicutes | Bacteria |
| 3.A.1.122.2 | ABC | Q1DEF9 | MXAN_0696 | O31712 | 1.35E-18 | 8 | 4 | 3.7 | Antimicrobial peptide resistance | Peptides | *Bacillus subtilis* | Firmicutes | Bacteria |
| 3.A.1.122.2 | ABC | Q1DG32 | MXAN_0108 | O31712 | 3.79E-49 | 4 | 4 | 3.75 | Antimicrobial peptide resistance | Peptides | *Bacillus subtilis* | Firmicutes | Bacteria |
| 3.A.1.122.3 | ABC | Q1D9M5 | MXAN_2428 | Q8RKC0 | 1.97E-13 | 4 | 4 | 1.7 | Enterocin AS-48 | Toxins | *Enterococcus faecalis* | Firmicutes | Bacteria |
| 3.A.1.122.8 | ABC | Q1D0I7 | MXAN_5698 | Q73MJ4 | 4.00E-09 | 4 | 4 | 3.3 | Putative ABC3 permease | Unknown | *Treponema denticola* | Spirochaetes | Bacteria |
| 3.A.1.125.1 | ABC | Q1D382 | MXAN_4729 | P75957 | 4.76E-58 | 1 | 1 | 0.85 | Lipoprotein translocation | Proteins | *Escherichia coli* | Proteobacteria | Bacteria |
| 3.A.1.125.1 | ABC | Q1D381 | MXAN_4730 | P0ADC3 | 1.72E-32 | 13 | 4 | 3.15 | Lipoprotein translocation | Proteins | *Escherichia coli* | Proteobacteria | Bacteria |
| 3.A.1.125.1 | ABC | Q1D1A7 | MXAN_5419 | P0ADC3 | 3.34E-33 | 14 | 4 | 3.35 | Lipoprotein translocation | Proteins | *Escherichia coli* | Proteobacteria | Bacteria |
| 3.A.1.125.2 | ABC | Q1CXI9 | MXAN_6766 | Q7D911 | 1.00E-168 | 9 | 10 | 8.15 | Putative lipoprotein homologue | Proteins | *Mycobacterium tuberculosis* | Actinobacteria | Bacteria |
| 3.A.1.132.1 | ABC | Q1DC23 | MXAN_1548 | Q93LN1 | 9.39E-20 | 6 | 6 | 5.4 | Putative ABC transporter; may function in secretion of a macromolecule such as an exopolysaccharide | Unknown | *Cytophaga johnsonae* | Bacteroidetes | Bacteria |
| 3.A.1.132.4 | ABC | Q1D2T7 | MXAN_4879 | Q2SDB0 | 8.11E-25 | 6 | 8 | 3.3 | Uncharacterized ABC transporter (Gld family) | Unknown | *Hahella chejuensis* | Proteobacteria | Bacteria |
| 3.A.1.140.1 | ABC | Q1D0D9 | MXAN_5747 | P0AC31 | 6.45E-19 | 5 | 4 | 3.55 | The putative FtsX/FtsE ABC | Unknown | *Escherichia coli* | Proteobacteria | Bacteria |
| 3.A.1.141.1 | ABC | Q1CZN7 | MXAN_6001 | P74757 | 2.04E-33 | 6 | 5 | 4.6 | Ethyl (methyl; benzyl) viologen | Toxins | *Synechocystis sp.* | Cyanobacteria | Bacteria |
| 3.A.1.141.1 | ABC | Q1CZN6 | MXAN_6002 | P74256 | 3.55E-25 | 6 | 6 | 5.3 | Ethyl (methyl; benzyl) viologen | Toxins | *Synechocystis sp.* | Cyanobacteria | Bacteria |
| 3.A.1.201.8 | ABC | Q1DDB6 | MXAN_1097 | Q0WML0 | 2.55E-134 | 6 | 6 | 3.9 | Aluminum chelate protein | Aromatic compounds | *Arabidopsis thaliana* | Angiosperms | Eukarya |
| 3.A.1.201.8 | ABC | Q1D203 | MXAN_5167 | Q0WML0 | 1.33E-122 | 6 | 6 | 4.2 | Aluminum chelate protein | Aromatic compounds | *Arabidopsis thaliana* | Angiosperms | Eukarya |
| 3.A.1.210.7 | ABC | Q1DBD5 | MXAN_1790 | Q9XUJ1 | 1.22E-25 | 6 | 10 | 3.5 | Exports phytochelatin (γ(Glu-Cys)n)-Cd^2+^ complexes | Peptides | *Caenorhabditis elegans* | Nematoda | Eukarya |
| 3.A.2.1.1 | F-ATPase | Q1DFA0 | MXAN_0402 | P0AB98 | 7.07E-12 | 6 | 6 | 3.3 | H^+^-translocating F-type ATPase | Cations | *Escherichia coli* | Proteobacteria | Bacteria |
| 3.A.2.1.2 | F-ATPase | Q1DF98 | MXAN_0404 | P21904 | 2.73E-10 | 1 | 1 | 0.85 | H^+^-translocating F-type ATPase | Cations | *Propionigenium modestum* | Fusobacteria | Bacteria |
| 3.A.2.1.2 | F-ATPase | Q1DF99 | MXAN_0403 | P21905 | 1.01E-05 | 2 | 2 | 1.8 | Na^+^-translocating F-type ATPase | Cations | *Propionigenium modestum* | Fusobacteria | Bacteria |
| 3.A.3.2.4 | P-ATPase | Q1CYB0 | MXAN_6491 | P37367 | E+000 | 10 | 10 | 7.65 | Ca^2+^-ATPase | Cations | *Synechocystis sp.* | Cyanobacteria | Bacteria |
| 3.A.3.5.4 | P-ATPase | Q1D6W1 | MXAN_3415 | Q9ZHC7 | E+000 | 8 | 9 | 2.95 | Ag^+^-ATPase | Cations | *Salmonella typhimurium* | Proteobacteria | Bacteria |
| 3.A.3.5.4 | P-ATPase | Q1DDN3 | MXAN_0979 | Q9ZHC7 | E+000 | 8 | 9 | 2.95 | Ag^+^-ATPase | Cations | *Salmonella typhimurium* | Proteobacteria | Bacteria |
| 3.A.3.5.15 | P-ATPase | Q1D6V4 | MXAN_3422 | Q3MNJ6 | E+000 | 8 | 9 | 4.7 | Cu | Cations | *Enterococcus faecium* | Firmicutes | Bacteria |
| 3.A.3.7.1 | P-ATPase | Q1DFX4 | MXAN_0166 | P03961 | 1.63E-36 | 1 | 1 | 1 | K^+^-ATPase | Cations | *Escherichia coli* | Proteobacteria | Bacteria |
| 3.A.3.7.1 | P-ATPase | Q1DFX5 | MXAN_0165 | P03960 | E+000 | 7 | 7 | 7 | K^+^-ATPase | Cations | *Escherichia coli* | Proteobacteria | Bacteria |
| 3.A.3.7.1 | P-ATPase | Q1DFX6 | MXAN_0164 | P03959 | 2.21E-172 | 10 | 12 | 7.95 | K^+^-ATPase | Cations | *Escherichia coli* | Proteobacteria | Bacteria |
| 3.A.3.27.3 | P-ATPase | Q1D0Z1 | MXAN_5543 | A6Q500 | 1.96E-72 | 8 | 7 | 3.8 | Functionally uncharacterized ε-proteobacteria P-type ATPase; copper | Cations | *Nitratiruptor sp.* | Proteobacteria | Bacteria |
| 3.A.3.32.6 | P-ATPase | Q1D4H8 | MXAN_4270 | A5UJX0 | 1.06E-90 | 4 | 2 | 1.85 | Functionally uncharacterized P-type ATPase family 32 | Cations | *Methanobrevibacter smithii* | Euryarchaeota | Archaea |
| 3.A.5.1.1 | Sec | Q1D8I8 | MXAN_2818 | P0AG99 | 7.74E-09 | 3 | 3 | 2.2 | General secretory pathway complex | Proteins | *Escherichia coli* | Proteobacteria | Bacteria |
| 3.A.5.2.2 | Sec | Q1D3B7 | MXAN_4690 | Q50635 | 1.39E-21 | 5 | 6 | 1.7 | General secretory pathway | Proteins | *Mycobacterium tuberculosis* | Actinobacteria | Bacteria |
| 3.A.5.2.2 | Sec | Q1D755 | MXAN_3319 | P0A5Z2 | 2.67E-100 | 10 | 10 | 7.75 | General secretory pathway | Proteins | *Mycobacterium tuberculosis* | Actinobacteria | Bacteria |
| 3.A.6.1.1 | IIISP | Q1D9L4 | MXAN_2439 | Q7BFA4 | 2.04E-19 | 1 | 3 | 0.5 | Type III protein secretion complex | Unknown | *Yersinia enterocolitica* | Proteobacteria | Bacteria |
| 3.A.6.1.1 | IIISP | Q1D0N8 | MXAN_5646 | Q7BFA7 | 2.22E-09 | 2 | 2 | 2.3 | Type III protein secretion complex | Unknown | *Yersinia enterocolitica* | Proteobacteria | Bacteria |
| 3.A.6.1.1 | IIISP | Q1D0P0 | MXAN_5644 | Q93KT4 | 2.46E-38 | 4 | 5 | 2.5 | Type III protein secretion complex | Unknown | *Yersinia enterocolitica* | Proteobacteria | Bacteria |
| 3.A.6.1.1 | IIISP | Q1D0N7 | MXAN_5647 | Q9ZA77 | 1.24E-43 | 4 | 4 | 3 | Type III protein secretion complex | Unknown | *Yersinia enterocolitica* | Proteobacteria | Bacteria |
| 3.A.6.1.1 | IIISP | Q1D9J0 | MXAN_2463 | P0C2V3 | 4.61E-176 | 7 | 7 | 6.55 | Type III protein secretion complex | Unknown | *Yersinia enterocolitica* | Proteobacteria | Bacteria |
| 3.A.6.3.1 | IIISP | Q1D933 | MXAN_2621 | O84671 | 7.34E-07 | 1 | 1 | 0.95 | Chlamydial type III secretion complex | Unknown | *Chlamydia trachomatis* | Chlamydiae | Bacteria |
| 3.A.6.3.1 | IIISP | Q1D9K5 | MXAN_2448 | O84567 | 6.41E-17 | 2 | 2 | 2.2 | Chlamydial type III secretion complex | Unknown | *Chlamydia trachomatis* | Chlamydiae | Bacteria |
| 3.A.6.3.1 | IIISP | Q1D9K3 | MXAN_2450 | O84093 | 1.15E-54 | 4 | 5 | 3.5 | Chlamydial type III secretion complex | Unknown | *Chlamydia trachomatis* | Chlamydiae | Bacteria |
| 3.A.6.3.1 | IIISP | Q1D0N9 | MXAN_5645 | O84568 | 3.93E-14 | 7 | 5 | 3.85 | Chlamydial type III secretion complex | Unknown | *Chlamydia trachomatis* | Chlamydiae | Bacteria |
| 3.A.6.3.1 | IIISP | Q1D9K6 | MXAN_2447 | O84566 | 9.15E-51 | 4 | 5 | 4 | Chlamydial type III secretion complex | Unknown | *Chlamydia trachomatis* | Chlamydiae | Bacteria |
| 3.A.6.3.1 | IIISP | Q1D9K4 | MXAN_2449 | O84568 | 1.68E-16 | 6 | 5 | 4.75 | Chlamydial type III secretion complex | Unknown | *Chlamydia trachomatis* | Chlamydiae | Bacteria |
| 3.A.6.3.1 | IIISP | Q1D0P1 | MXAN_5643 | O84092 | 6.20E-140 | 8 | 7 | 6.35 | Chlamydial type III secretion complex | Unknown | *Chlamydia trachomatis* | Chlamydiae | Bacteria |
| 3.A.7.15.1 | IVSP | Q1D3F6 | MXAN_4651 | Q9XC05 | 4.36E-09 | 5 | 5 | 3.3 | Nonspecific tight adherence fibril (pilus) secretion septum | Unknown | *Actinobacillus actinomycetemcomitans* | Proteobacteria | Bacteria |
| 3.A.10.1.5 | H+-Ppase | Q1DBQ0 | MXAN_1671 | B0M926 | 8.81E-158 | 16 | 16 | 12.35 | Na^+^-translocating PPase | Cations | *Moorella thermoacetica* | Arthropoda | Eukarya |
| 3.A.11.1.1 | DNA-T | Q1D928 | MXAN_2626 | P39695 | 1.11E-39 | 10 | 12 | 2.9 | DNA translocase | DNA/DNA Proteins | *Bacillus subtilis* | Firmicutes | Bacteria |
| 3.A.11.1.3 | MTB | Q1D9E2 | MXAN_2512 | Q8VRL3 | 1.13E-65 | 3 | 3 | 0.55 | Multicomponent DNA uptake competence | Proteins | *Klebsiella pneumoniae* | Proteobacteria | Bacteria |
| 3.A.15.1.1 | MTB | Q1D9X1 | MXAN_2332 | P15643 | 2.96E-06 | 1 | 1 | 0.55 | Pullulanase secretion system | Proteins | *Klebsiella pneumoniae* | Proteobacteria | Bacteria |
| 3.A.15.1.1 | MTB | Q1D9D9 | MXAN_2515 | P15643 | 1.46E-07 | 1 | 1 | 0.9 | Pullulanase secretion system | Proteins | *Klebsiella pneumoniae* | Proteobacteria | Bacteria |
| 3.A.15.2.1 | MTB | Q1DCJ7 | MXAN_1369 | Q59652 | 8.26E-06 | 1 | 1 | 0.7 | Pilin secretion/fimbrial assembly system | Proteins | *Pseudomonas aeruginosa* | Proteobacteria | Bacteria |
| 3.A.15.2.1 | MTB | Q1DDJ5 | MXAN_1017 | Q59652 | 2.70E-07 | 1 | 1 | 1 | Pilin secretion/fimbrial assembly system | Proteins | *Pseudomonas aeruginosa* | Proteobacteria | Bacteria |
| 3.A.15.2.1 | MTB | Q59589 | MXAN_5783 | Q60148 | 2.02E-08 | 1 | 1 | 1.1 | Pilin secretion/fimbrial assembly system | Proteins | *Pseudomonas aeruginosa* | Proteobacteria | Bacteria |
| 3.A.15.2.1 | MTB | O30387 | MXAN_5779 | P22610 | 6.41E-29 | 7 | 6 | 2.95 | Pilin secretion/fimbrial assembly system | Proteins | *Pseudomonas aeruginosa* | Proteobacteria | Bacteria |
| 3.A.15.2.1 | MTB | Q1D0A0 | MXAN_5786 | P22609 | 7.64E-92 | 4 | 4 | 3.1 | Pilin secretion/fimbrial assembly system | Proteins | *Pseudomonas aeruginosa* | Proteobacteria | Bacteria |
| 3.A.15.3.1 | MTB | Q1D5R7 | MXAN_3824 | Q9XD71 | 7.10E-21 | 1 | 1 | 0.8 | Legionella secretion pathway/fimbrial assembly system | Proteins | *Legionella pneumophila* | Proteobacteria | Bacteria |
| 3.A.15.3.1 | MTB | Q1CVS1 | MXAN_7395 | Q9XD71 | 5.71E-09 | 1 | 1 | 0.8 | Legionella secretion pathway/fimbrial assembly system | Proteins | *Legionella pneumophila* | Proteobacteria | Bacteria |
| 3.A.15.3.1 | MTB | Q1D9E5 | MXAN_2509 | Q9XD71 | 2.16E-08 | 1 | 1 | 0.9 | Legionella secretion pathway/fimbrial assembly system | Proteins | *Legionella pneumophila* | Proteobacteria | Bacteria |
| 3.A.15.3.1 | MTB | Q1D9E4 | MXAN_2510 | Q9XD71 | 1.49E-21 | 1 | 1 | 1.05 | Legionella secretion pathway/fimbrial assembly system | Proteins | *Legionella pneumophila* | Proteobacteria | Bacteria |
| 3.B.1.1.2 | NaT-DC | Q1DDA0 | MXAN_1113 | Q57079 | E+000 | 1 | 1 | 0.85 | Na^+^-transporting methylmalonyl-CoA decarboxylase | Cations | *Veillonella parvula* | Firmicutes | Bacteria |
| 3.B.1.1.2 | NaT-DC | Q1D5Y2 | MXAN_3759 | Q57079 | 4.04E-95 | 3 | 1 | 0.9 | Na^+^-transporting methylmalonyl-CoA decarboxylase | Cations | *Veillonella parvula* | Firmicutes | Bacteria |
| 3.B.1.1.5 | NaT-DC | Q1DG59 | MXAN_0081 | Q9V0A4 | 4.40E-63 | 1 | 3 | 0.2 | Putative Na^+^-transporting methylmalonyl-CoA decarboxylase | Cations | *Pyrococcus abyssi* | Euryarchaeota | Archaea |
| 3.B.1.1.5 | NaT-DC | Q1D8V2 | MXAN_2704 | Q9V0A4 | 5.69E-14 | 2 | 3 | 0.85 | Putative Na^+^-transporting methylmalonyl-CoA decarboxylase | Cations | *Pyrococcus abyssi* | Euryarchaeota | Archaea |
| 3.D.1.1.1 | NDH | Q1DDD1 | MXAN_1082 | P33607 | 7.79E-94 | 18 | 18 | 9.15 | NADH-quinone oxidoreductase subunit L | Electrons | *Escherichia coli* | Proteobacteria | Bacteria |
| 3.D.1.2.1 | NDH | Q1D8S2 | MXAN_2734 | P29919 | 8.94E-22 | 3 | 3 | 2.5 | NADH-quinone oxidoreductase subunit L | Electrons | *Paracoccus denitrificans* | Proteobacteria | Bacteria |
| 3.D.1.2.1 | NDH | Q1DDD0 | MXAN_1083 | P29923 | 2.91E-20 | 3 | 3 | 2.95 | NADH-quinone oxidoreductase subunit L | Electrons | *Paracoccus denitrificans* | Proteobacteria | Bacteria |
| 3.D.1.2.1 | NDH | Q1DDD2 | MXAN_1081 | P29925 | 6.62E-106 | 15 | 14 | 8.15 | NADH-quinone oxidoreductase subunit L | Electrons | *Paracoccus denitrificans* | Proteobacteria | Bacteria |
| 3.D.1.5.1 | NDH | Q1D8S9 | MXAN_2727 | Q746T2 | 1.92E-64 | 10 | 8 | 4.05 | Proton-translocating NADH dehydrogenase I | Cations | *Geobacter sulfurreducens* | Proteobacteria | Bacteria |
| 3.D.4.3.1 | COX | Q1CX47 | MXAN_6912 | O54596 | 2.10E-10 | 9 | 9 | 6.6 | Proton-translocating Cytochrome oxidase | Cations | *Halobacterium halobium* | Euryarchaeota | Archaea |
| 3.D.4.3.1 | COX | Q1D0Z3 | MXAN_5541 | P33518 | 1.71E-10 | 15 | 13 | 7.75 | Proton-translocating Cytochrome oxidase | Cations | *Halobacterium halobium* | Euryarchaeota | Archaea |
| 3.D.4.4.1 | COX | Q1CZF0 | MXAN_6088 | P24012 | 3.61E-18 | 5 | 5 | 3.95 | Proton-translocating Cytochrome oxidase | Cations | *Bacillus subtilis* | Firmicutes | Bacteria |
| 3.D.4.4.1 | COX | Q1D1K4 | MXAN_5318 | P24009 | 1.39E-45 | 9 | 9 | 6.5 | Proton-translocating Cytochrome oxidase | Cations | *Bacillus subtilis* | Firmicutes | Bacteria |
| 3.D.4.4.1 | COX | Q1DEQ3 | MXAN_0602 | P12946 | 8.84E-13 | 8 | 8 | 7.65 | Proton-translocating Cytochrome oxidase | Cations | *Bacillus subtilis* | Firmicutes | Bacteria |
| 3.D.4.4.1 | COX | Q1D5M5 | MXAN_3868 | P24010 | 1.64E-101 | 12 | 14 | 12.3 | Proton-translocating Cytochrome oxidase | Cations | *Bacillus subtilis* | Firmicutes | Bacteria |
| 3.D.4.4.1 | COX | Q1CZF1 | MXAN_6087 | P24010 | 4.22E-106 | 12 | 14 | 12.8 | Proton-translocating Cytochrome oxidase | Cations | *Bacillus subtilis* | Firmicutes | Bacteria |
| 3.D.4.6.1 | COX | Q1CZF2 | MXAN_6086 | P08306 | 3.28E-19 | 2 | 3 | 1.9 | Proton-translocating Cytochrome oxidase | Cations | *Paracoccus denitrificans* | Proteobacteria | Bacteria |
| 3.D.4.6.1 | COX | Q1D5M4 | MXAN_3869 | P08306 | 2.49E-16 | 3 | 3 | 2 | Proton-translocating Cytochrome oxidase | Cations | *Paracoccus denitrificans* | Proteobacteria | Bacteria |
| 3.D.4.7.1 | COX | Q1D5M6 | MXAN_3867 | P00415 | 4.35E-14 | 5 | 6 | 3.95 | Proton-translocating Cytochrome oxidase | Cations | *Bos taurus* | Chordata | Eukarya |
| 3.D.7.2.2 | HHO | Q1DFX9 | MXAN_0161 | P31875 | 3.81E-07 | 4 | 5 | 2.7 | Membrane-bound Ni/Fe-hydrogenase complex | Cations | *Wolinella succinogenes* | Proteobacteria | Bacteria |
| 3.D.9.1.1 | F420H2DH | Q1DDC9 | MXAN_1084 | Q9P9F7 | 8.78E-16 | 5 | 5 | 4.75 | H^+^-translocating F420H2 Dehydrogenase | Cations | *Methanosarcina mazei* | Euryarchaeota | Archaea |
| 3.D.9.1.1 | F420H2DH | Q1DDD3 | MXAN_1080 | Q9P9F3 | 5.82E-66 | 14 | 13 | 7.05 | H^+^-translocating F420H2 Dehydrogenase | Cations | *Methanosarcina mazei* | Euryarchaeota | Archaea |
| 4.A.6.1.7 | Man | Q1CY69 | MXAN_6532 | Q5M5W8 | 8.93E-29 | 7 | 4 | 1.9 | The glucose/mannose/2-deoxyglucose/fructose phosphotransferase systems | Sugars & polyols | *Streptococcus thermophilus* | Firmicutes | Bacteria |
| 4.A.6.1.9 | Man | Q1CY68 | MXAN_6533 | Q97N93 | 0.0001226 | 5 | 6 | 4.05 | Putative fructose PTS | Sugars & polyols | *Streptococcus pneumoniae* | Firmicutes | Bacteria |
| 4.C.1.1.4 | FAT | Q1DFR6 | MXAN_0225 | P69451 | 9.60E-65 | 2 | 2 | 0.05 | Long chain fatty acyl CoA synthase (ligase); phospholipids | Lipids | *Escherichia coli* | Proteobacteria | Bacteria |
| 4.C.1.1.4 | FAT | Q1DFS5 | MXAN_0216 | P69451 | 2.17E-65 | 1 | 2 | 0.1 | Long chain fatty acyl CoA synthase (ligase); phospholipids | Lipids | *Escherichia coli* | Proteobacteria | Bacteria |
| 4.C.1.1.4 | FAT | Q1D691 | MXAN_3645 | P69451 | 3.80E-39 | 2 | 2 | 0.1 | Long chain fatty acyl CoA synthase (ligase); phospholipids | Lipids | *Escherichia coli* | Proteobacteria | Bacteria |
| 4.C.1.1.4 | FAT | Q1D4E3 | MXAN_4305 | P69451 | 3.97E-24 | 1 | 2 | 0.2 | Long chain fatty acyl CoA synthase (ligase); phospholipids | Lipids | *Escherichia coli* | Proteobacteria | Bacteria |
| 4.C.1.1.4 | FAT | Q1D446 | MXAN_4405 | P69451 | 8.47E-29 | 2 | 2 | 0.4 | Long chain fatty acyl CoA synthase (ligase); phospholipids | Lipids | *Escherichia coli* | Proteobacteria | Bacteria |
| 5.A.1.1.1 | DsbD | Q1D2G8 | MXAN_4999 | P36655 | 2.32E-56 | 9 | 9 | 5.85 | Disulfide bond oxidoreductase-D | Electrons | *Escherichia coli* | Proteobacteria | Bacteria |
| 5.A.1.2.1 | DsbD | Q1CZ86 | MXAN_6156 | P45706 | 2.02E-26 | 5 | 6 | 5.05 | Cytochrome c-type biogenesis protein | Unknown | *Bacillus subtilis* | Firmicutes | Bacteria |
| 5.A.1.5.1 | DsbD | Q1DFK4 | MXAN_0289 | O33918 | 9.16E-49 | 9 | 7 | 5.95 | Suppressor of copper-sensitivity B | Cations | *Salmonella typhimurium* | Proteobacteria | Bacteria |
| 5.A.3.2.1 | PMO | Q1D4A9 | MXAN_4341 | P24183 | 1.40E-19 | 1 | 2 | 0.55 | Anaerobic, respiratory, membrane-bound formate dehydrogenase. | Electrons | *Escherichia coli* | Proteobacteria | Bacteria |
| 5.A.3.3.3 | PMO | Q1CZF8 | MXAN_6080 | Q9HR72 | 1.95E-09 | 10 | 10 | 5.6 | Anaerobic dimethylsulfoxide /trimethylamine-N-oxide reductase | Unknown | *Halobacterium halobium* | Euryarchaeota | Archaea |
| 5.A.3.3.3 | PMO | Q1D0X8 | MXAN_5556 | Q9HR72 | 9.73E-12 | 10 | 10 | 5.7 | Anaerobic dimethylsulfoxide /trimethylamine-N-oxide reductase | Unknown | *Halobacterium halobium* | Euryarchaeota | Archaea |
| 5.A.3.3.3 | PMO | Q1D5L9 | MXAN_3874 | Q9HR72 | 7.67E-12 | 10 | 10 | 5.95 | Anaerobic dimethylsulfoxide /trimethylamine-N-oxide reductase | Unknown | *Halobacterium halobium* | Euryarchaeota | Archaea |
| 5.A.3.4.2 | PMO | Q1DA91 | MXAN_2210 | P52005 | 9.19E-06 | 1 | 1 | 0.65 | Anaerobic trimethylamine-N-oxide reductase 2 | Electrons | *Escherichia coli* | Proteobacteria | Bacteria |
| 5.A.4.1.1 | SDH | Q1D1U7 | MXAN_5224 | Q65GF4 | 7.07E-92 | 2 | 1 | 0.9 | Succinate:menaquinone oxidoreductase | Anions | *Bacillus licheniformis* | Firmicutes | Bacteria |
| 5.A.4.1.1 | SDH | Q1DDE1 | MXAN_1072 | Q65GF3 | 6.22E-20 | 5 | 5 | 3.9 | Succinate:menaquinone oxidoreductase | Anions | *Bacillus licheniformis* | Firmicutes | Bacteria |
| 8.A.1.6.2 | MFP | Q1D5H1 | MXAN_3922 | P76397 | 2.55E-27 | 1 | 1 | 0.1 | Multidrug | Multiple drugs | *Escherichia coli* | Proteobacteria | Bacteria |
| 8.A.3.1.1 | MPA1-C or MPA1 + C | Q1DDI7 | MXAN_1025 | P33698 | 9.08E-42 | 1 | 3 | 0.85 | PST-type exopolysaccharide | Carbohydrates | *Sinorhizobium meliloti* | Proteobacteria | Bacteria |
| 8.A.3.1.1 | MPA1-C or MPA1 + C | Q1D7E7 | MXAN_3227 | P33698 | 7.95E-09 | 1 | 3 | 0.85 | PST-type exopolysaccharide | Carbohydrates | *Sinorhizobium meliloti* | Proteobacteria | Bacteria |
| 8.A.3.2.3 | MPA1-C or MPA1 + C | Q1D727 | MXAN_3348 | Q93E02 | 1.89E-07 | 2 | 1 | 0.8 | PST-type exopolysaccharide | Carbohydrates | *Streptococcus thermophilus* | Firmicutes | Bacteria |
| 8.A.21.2.1 | Stomatin | Q1DE78 | MXAN_0779 | O59180 | 4.21E-61 | 1 | 3 | 0.85 | Stomatin homologue | Proteins | *Pyrococcus horikoshii* | Euryarchaeota | Archaea |
| 8.A.21.2.1 | Stomatin | Q1D7J9 | MXAN_3171 | O59180 | 4.94E-06 | 2 | 3 | 0.85 | Stomatin homologue | Proteins | *Pyrococcus horikoshii* | Euryarchaeota | Archaea |
| 8.A.21.2.1 | Stomatin | Q1D5A9 | MXAN_3984 | O59180 | 9.76E-30 | 1 | 3 | 0.9 | Stomatin homologue | Proteins | *Pyrococcus horikoshii* | Euryarchaeota | Archaea |
| 8.A.21.2.1 | Stomatin | Q1DE77 | MXAN_0780 | O59179 | 8.88E-39 | 4 | 7 | 3.9 | Stomatin homologue | Proteins | *Pyrococcus horikoshii* | Euryarchaeota | Archaea |
| 9.A.10.2.1 | ILT | Q1CZU0 | MXAN_5946 | Q58AJ4 | 2.89E-69 | 6 | 7 | 2.5 | Pb^2+^ | Cations | *Ralstonia metallidurans* | Proteobacteria | Bacteria |
| 9.A.19.1.2 | Arv1 | Q1DFC1 | MXAN_0378 | Q5SMG8 | 5.26E-80 | 5 | 5 | 4.05 | Mg^2+^ | Cations | *Thermus thermophilus* | Deinococcus | Bacteria |
| 9.A.19.1.2 | Arv1 | Q1CVM5 | MXAN_7443 | Q5SMG8 | 5.31E-78 | 5 | 5 | 4.25 | Mg^2+^ | Cations | *Thermus thermophilus* | Deinococcus | Bacteria |
| 9.A.30.1.2 | TerC | Q1DC36 | MXAN_1535 | F0N2E7 | 1.24E-43 | 9 | 9 | 5.3 | Unknown | Unknown | *Escherichia coli* | Proteobacteria | Bacteria |
| 9.A.30.1.2 | TerC | Q1D6Q0 | MXAN_3478 | F0N2E7 | 1.23E-41 | 9 | 9 | 6.5 | Unknown | Unknown | *Escherichia coli* | Proteobacteria | Bacteria |
| 9.A.31.1.1 | SdpAB | Q1CXZ0 | MXAN_6615 | O34616 | 4.85E-53 | 6 | 6 | 5.1 | Peptide antibiotic-like killing factor | Toxic peptides | *Bacillus subtilis* | Firmicutes | Bacteria |
| 9.A.34.1.1 | VISP | Q1D309 | MXAN_4803 | Q9KN50 | 6.18E-11 | 1 | 2 | 0.8 | Type VI secretion system (antimicrobial) | Virulence factors | *Vibrio cholerae* | Proteobacteria | Bacteria |
| 9.A.34.1.1 | VISP | Q1D308 | MXAN_4804 | Q9KN45 | 7.50E-52 | 3 | 3 | 1.9 | Type VI secretion system (antimicrobial) | Virulence factors | *Vibrio cholerae* | Proteobacteria | Bacteria |
| 9.A.40.2.1 | HCC | Q1D316 | MXAN_4795 | P54428 | 9.35E-47 | 2 | 4 | 1.5 | Unknown | Unknown | *Bacillus subtilis* | Firmicutes | Bacteria |
| 9.A.40.2.1 | HCC | Q1D2R3 | MXAN_4903 | P54428 | 9.02E-70 | 3 | 4 | 2 | Unknown | Unknown | *Bacillus subtilis* | Firmicutes | Bacteria |
