# Supplementary material for: Comparative genomics of transport proteins in developmental bacteria: Myxococcus xanthus and Streptomyces coelicolor
Source: BMC Microbiol. 2013 Dec 5;13:279. doi: 10.1186/1471-2180-13-279 (PMC3924187; doi:10.1186/1471-2180-13-279)
Supplement: Additional file 3: Table S3 — Chromosomal distribution of Sco transporters. Sco transport proteins distributed by chromosomal arms and core. [file 1471-2180-13-279-S3.docx]

| **Family** | **Arm1** | **Arm2** | **Core** | **Total** |
| --- | --- | --- | --- | --- |
| 2.A.1.1 | 0 | 1 | 2 | 3 |
| 2.A.1.2 | 2 | 3 | 6 | 11 |
| 2.A.1.3 | 4 | 20 | 21 | 45 |
| 2.A.1.6 | 2 | 2 | 2 | 6 |
| 2.A.1.11 | 0 | 2 | 1 | 3 |
| 2.A.1.14 | 2 | 0 | 1 | 3 |
| 2.A.1.15 | 1 | 1 | 1 | 3 |
| 2.A.1.17 | 0 | 2 | 1 | 3 |
| 2.A.1.21 | 1 | 2 | 3 | 6 |
| 2.A.1.30 | 0 | 2 | 2 | 4 |
| 2.A.1.35 | 0 | 1 | 0 | 1 |
| 2.A.1.54 | 0 | 0 | 1 | 1 |
| 2.A.1.60 | 1 | 3 | 3 | 7 |
| 2.A.1.67 | 0 | 1 | 4 | 5 |
| 2.A.3 | 1 | 8 | 8 | 17 |
| 2.A.15 | 0 | 1 | 0 | 1 |
| 2.A.21 | 1 | 2 | 5 | 8 |
| 2.A.25 | 0 | 1 | 0 | 1 |
| 2.A.39 | 1 | 2 | 2 | 5 |
| 3.A.1.1 | 21 | 24 | 29 | 74 |
| 3.A.1.2 | 2 | 4 | 3 | 9 |
| 3.A.1.3 | 0 | 0 | 5 | 5 |
| 3.A.1.4 | 2 | 2 | 2 | 6 |
| 3.A.1.5 | 0 | 10 | 7 | 17 |
| 3.A.1.11 | 0 | 0 | 3 | 3 |
| 3.A.1.12 | 0 | 2 | 3 | 5 |
| 3.A.1.14 | 3 | 2 | 3 | 8 |
| 3.A.1.18 | 0 | 2 | 0 | 2 |
| 3.A.1.20 | 0 | 1 | 0 | 1 |
| 3.A.1.23 | 0 | 0 | 2 | 2 |
| 3.A.1.32 | 0 | 0 | 2 | 2 |
| 3.A.1.105 | 0 | 0 | 10 | 10 |
| 3.A.1.106 | 2 | 1 | 3 | 6 |
| 3.A.1.112 | 1 | 0 | 0 | 1 |
| 3.A.1.119 | 3 | 1 | 2 | 6 |
| 3.A.1.122 | 0 | 0 | 1 | 1 |
| 3.A.1.125 | 2 | 2 | 5 | 9 |
| 3.A.1.127 | 0 | 2 | 0 | 2 |
| 3.A.1.129 | 0 | 0 | 1 | 1 |
| 3.A.1.134 | 1 | 0 | 0 | 1 |
| 3.A.1.135 | 0 | 0 | 1 | 1 |
| 3.A.1.204 | 0 | 0 | 1 | 1 |
| **Summary** | 53 | 107 | 146 | 306 |
